# Screening and surveillance practices for Multiple Endocrine Neoplasia type 1‐related Neuroendocrine Tumours in European Neuroendocrine Tumor Society Centers of Excellence (ENETS CoE)—An ENETS MEN1 task force questionnaire study

**DOI:** 10.1111/jne.13468

**Published:** 2024-11-26

**Authors:** Carolina R. C. Pieterman, Simona Grozinsky‐Glasberg, Dermot O'Toole, James R. Howe, Valentina Ambrosini, Susana H. Belli, Mikkel Andreassen, Nehara Begum, Timm Denecke, Antongiulio Faggiano, Massimo Falconi, Jo Grey, Ulrich P. Knigge, Teodora Kolarova, Bruno Niederle, Els Nieveen van Dijkum, Stefano Partelli, Andreas Pascher, Guido Rindi, Philippe Ruszniewski, Stefan Stättner, Timon Vandamme, Juan W. Valle, Marie‐Pierre Vullierme, Staffan Welin, Aurel Perren, Detlef K. Bartsch, Gregory K. Kaltsas, Gerlof D. Valk

**Affiliations:** ^1^ Department of Endocrine Oncology University Medical Center Utrecht Utrecht the Netherlands; ^2^ Neuroendocrine Unit, ENETS Center of Excellence, Division of Medicine, Hadassah Medical Center and Faculty of Medicine The Hebrew University Jerusalem Israel; ^3^ Department of Clinical Medicine, St James Hospital, Trinity College Dublin & National Centre for Neuroendocrine Tumours St Vincent's University Hospital Dublin Ireland; ^4^ University of Iowa Carver College of Medicine Iowa City Iowa USA; ^5^ Nuclear Medicine, Alma Mater Studiorum University of Bologna Bologna Italy; ^6^ Nuclear Medicine IRCCS Azienda Ospedaliero‐Universitaria di Bologna Bologna Italy; ^7^ Endocrinology Instituto Alexander Fleming Buenos Aires Argentina; ^8^ Department of Endocrinologt Copenhagen University Hospital Rigshospitalet Copenhagen Denmark; ^9^ Department of General‐, Visceral‐, Thoracic‐ and Endocrine Surgery, Johannes Wesling Klinikum Minden University Hospital of the Ruhr‐University Bochum (RUB) Minden Germany; ^10^ Klinik für Diagnostische und Interventionelle Radiologie Universitätsklinikum Leipzig Leipzig Germany; ^11^ Endocrinology Unit, Department of Clinical and Molecular Medicine, Sant'Andrea Hospital, ENETS Center of Excellence Sapienza University of Rome Rome Italy; ^12^ Pancreatic and Transplantation Surgical Unit IRCCS San Raffaele Hospital, Università Vita‐Salute Milan Italy; ^13^ AMEND (Association for Multiple Endocrine Neoplasia Disorder) Tornbridge UK; ^14^ Department of Endocrinology and Department of Surgery and Transplantation, Rigshospitalet University of Copenhagen Copenhagen Denmark; ^15^ Executive Director International Neuroendocrine Cancer Alliance (INCA) Boston Massachusetts USA; ^16^ Division of Visceral Surgery, Department of General Surgery Medical University Vienna Austria; ^17^ Department of Surgery, Cancer Center Amsterdam University of Amsterdam Amsterdam The Netherlands; ^18^ Vita‐Salute San Raffaele University, IRCCS San Raffaele Scientific Institute Milan Italy; ^19^ Klinik für Allgemein‐, Viszeral‐ und Transplantationschirurgie, Universitätsklinikum Münster Münster Germany; ^20^ Department of Life Sciences and Public Health Università Cattolica del Sacro Cuore Rome Italy; ^21^ Department of Woman and Child Health Sciences and Public Health Fondazione Policlinico Universitario A. Gemelli IRCCS Rome Italy; ^22^ European NeuroEndocrine Tumor Society (ENETS) Center of Excellence Roma‐Gemelli Roma Italy; ^23^ Université de Paris, Beaujon Hospital Clichy France; ^24^ Department of General, Visceral and Vascular Surgery, Salzkammergut Klinikum Vöcklabruck, Oberösterreichische Gesundheitsholding OÖG Vöcklabruck Austria; ^25^ NETwerk, Department of Oncology Antwerp University Hospital Antwerp Belgium; ^26^ Integrated Personalized and Precision Oncology Network (IPPON), Center for Oncological Research (CORE) University of Antwerp Antwerp Belgium; ^27^ Division of Cancer Sciences University of Manchester Manchester UK; ^28^ Cholangiocarcinoma Foundation Herriman Utah USA; ^29^ Université Paris Diderot Paris France; ^30^ Service de Radiologie AP‐HP Hôpital Beaujon Clichy France; ^31^ Endocrine Oncology Department of Medical sciences Uppsala University Uppsala Sweden; ^32^ Institute of Tissue Medicine and Pathology University of Bern Bern Switzerland; ^33^ Department of Visceral‐, Thoracic and Vascular Surgery Philipps‐University Marburg Marburg Germany; ^34^ 1st Propaedeutic Department of Internal Medicine National and Kapodistrian University of Athens Athens Greece

**Keywords:** multiple endocrine neoplasia type 1, neuroendocrine tumour, screening, surveillance, survey

## Abstract

Multiple Endocrine Neoplasia type 1 (MEN1) Clinical Practice Guidelines (2012) are predominantly based on expert opinion due to limited available evidence at the time, leaving room for interpretation and variation in practices. Evidence on the natural course of MEN1‐related neuroendocrine tumours (NET) and the value of screening programs has increased and new imaging techniques have emerged. The aim of this study is to provide insight in the current practices of screening and surveillance for MEN1‐related NETs in ENETS Centers of Excellence (CoEs). A clinical practice questionnaire was distributed among all 65 ENETS CoEs. Response rate was 91% (59/65). In 14% of CoEs <10 patients, in 50% 10–49, in 31% 50–100 and in 3 centres (5%) >100 patients with MEN1 are seen. Practices with regard to screening and surveillance of NETs were markedly heterogeneous. Differences between countries were noted in the use of gut hormones for biochemical screening and the choice for imaging modality for screening/surveillance of pancreatic NETs (PanNETs). Magnetic resonance imaging (MRI) is the preferred modality for screening and surveillance of PanNETs, whereas this is computed tomography (CT) for thoracic NETs. Practices regarding screening for thoracic NETs were more homogeneous among larger volume CoEs, with longer screening intervals. The majority of CoEs tailored the surveillance of small pancreatic and lung NETs to observed growth rate. 68% of CoEs advise patients with clinical MEN1 with negative genetic testing to undergo periodic screening like mutation‐positive patients. In conclusion, there is still marked heterogeneity in practice, although there are also common trends. Differences were sometimes associated with volume or country, but often no association was found. This underscores the need for clear and evidence‐based practice recommendations.

## 1 INTRODUCTION

Patients with the autosomal dominant inherited tumour syndrome Multiple Endocrine Neoplasia type 1 (MEN1) are at high risk of developing neuroendocrine tumours (NETs) of the pancreas and duodenum with a cumulative incidence of 80% by the age of 80,^1^, ^2^ followed by lung NETs with a point prevalence up to 30% based on radiological diagnosis, or ~5% only counting histologically confirmed cases.^3^ Thymic NETs are rare with a point prevalence of 2–8% in different cohorts and occur almost exclusively in males.^3^ Finally, gastric NETs occur in patients with the Zollinger‐Ellison syndrome (ZES).^4^ Patients with MEN1 have a decreased life expectancy, with the majority of deaths being MEN1‐related, most often due to metastatic duodenopancreatic and thymic NETs.^1^, ^5^

The identification of the *MEN1* gene in 1997^6^, ^7^ enabled a genetic diagnosis of the syndrome and pre‐symptomatic identification of gene carriers. This led to the first recommendations for screening and surveillance of MEN1‐related tumours to prevent tumour‐related morbidity and mortality in 2001.^8^ Recommendations were updated in 2012.^9^ With limited available evidence at the time, most of the recommendations were based on expert opinion, leaving room for interpretation and variation in practice. Since that time, large single‐centre and multi‐centre (population‐based) cohorts have provided new evidence regarding the natural history of MEN1‐related NETs and the value of screening protocols. Novel imaging techniques, such as somatostatin receptor positron emission–computed tomography (SST‐PET/CT), are now widely available. Additionally, awareness has increased regarding the negative effects of a comprehensive screening program such as high cumulative exposure to ionising radiation, identification of small tumours without clinical consequences, psychological effects of frequent investigations and cost.^10^ For duodenopancreatic NETs, this new evidence has been incorporated in an international consensus statement in 2021.^11^ With the widespread use of genetic testing, an important group of patients are those who meet clinical diagnostic criteria for MEN1 (2 of the 3 main manifestations) but have negative genetic testing. Novel data show that these patients do not seem to have the same risk of tumour development^1^, ^12^ and it is therefore debated whether they would derive similar benefit from screening and surveillance as patients with a confirmed *MEN1* mutation.

The European Neuroendocrine Tumor Society (ENETS) has established several task forces to clarify existing standards of care amongst Centers of Excellence (CoEs) and address areas of uncertainty according to current guidelines and/or any existing unmet needs. As a starting point and to identify significant gaps in knowledge, the ENETS MEN1 Task Force aimed to provide insight in the current practices of screening and surveillance for MEN1‐related NETs in ENETS CoEs through the development, distribution and analysis of a clinical practice questionnaire (CPQ) among all CoEs.

## 2 METHODS

Members of the Task Force developed a questionnaire consisting of questions regarding: (1) organisation of care, (2) screening and surveillance of adults with MEN1 and (3) follow‐up of genotype negative patients with a clinical MEN1 diagnosis. Outcomes of screening and surveillance in children will be reported separately. After several iterations, a final version was agreed upon (Supplemental Data 1). Questions either were formulated as multiple‐choice options with the possibility of a single answer or ‘select all that apply’. When appropriate, an option of ‘other’ was added with a free text field to elaborate. The questionnaire was built as a survey in Castor Electronic Data Capture^13^ and send out electronically between January and March 2022 to the 65 heads and/or contacts of all accredited CoEs up to March 2022. It was requested to forward the questionnaire to the lead provider coordinating MEN1 patient care at the CoE to fill out this questionnaire on behalf of the CoE. In cases of initial non‐response, the members of the Task Force approached CoEs.

### 2.1 Analysis

Categorical variables were reported as numbers and percentages. When practices were heterogeneous, we looked for associations with patient number and country. Patient number was dichotomised to *n* < 50 vs. *n* ≥ 50 to determine if practices differed by MEN1 patient volume at CoEs. Differences were tested using the chi‐square test or Fisher's exact test as appropriate. To determine whether there was a country‐specific pattern in practices, we only considered countries from which ≥3 CoEs had answered a particular question, and proportions were compared. No formal statistical tests were performed because not all CoEs could be included in this analysis and numbers were low. The analyses were performed using IBM SPSS statistics for windows (IBM Corp. Released 2022. IBM SPSS Statistics for Windows, Version 29.0.1 Armonk, NY: IBM Corp).

## 3 RESULTS

**FIGURE 1:**
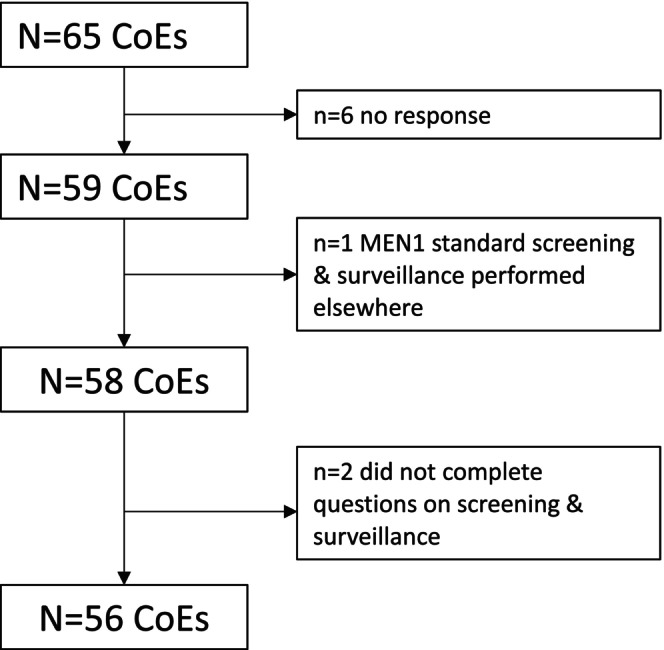
Flow chart of survey responses. CoE Center of Excellence, MEN1 Multiple Endocrine Neoplaisa Type 1.

The response rate was 91% (Figure 1). Of the 59 responding CoEs, 58 indicated seeing patients with MEN1 for regular screening and surveillance and 56 answered the questions pertaining to screening and surveillance practices (Figure 1). The two CoEs that did not answer questions had a MEN1 patient volume of <10 in Q1‐Q2 2022. Thus, results regarding screening and surveillance of patients with MEN1 reflected 86% of CoEs. Endocrinologists (Table 1) answered most of the questionnaires (61%).

**TABLE 1:** Patient population and organisation of care.

	*N* (%)
*N* = 59 CoEs	
Discipline filling out the questionnaire	
Endocrinologist^a^	36 (61)
Medical Oncologist	9 (15)
Gastroenterologist	7 (12)
Surgeon	5 (9)
Internist	1 (2)
Nuclear medicine physician	1 (2)
*N* = 58 CoEs	
Adult MEN1 Patient Volume	
<10	8 (14)
10–49	29 (50)
50–100	18 (31)
>100	3 (5)
Lead provider for MEN1 screening/surveillance in adults	
Endocrinologist	36 (61)
Endocrinologist and medical oncologist	7 (12)
Endocrinologist and gastroenterologist	5 (9)
Medical oncologist	3 (5)
Endocrinologist and surgeon	3 (5)
Endocrinologist, gastroenterologist, medical oncologist	1 (2)
Endocrinologist, medical oncologist, internist	1 (2)
Endocrinologist, gastroenterologist, medical oncologist, and surgeon	1 (2)
Referral physician	1 (2)
Genetic counselling provided by	
Geneticist/genetic counsellor at the institution	37 (64)
Geneticist/genetic counsellor outside the institution	11 (19)
The provider of the periodic screening/regular follow‐up	9 (16)
All of the above may be used	1 (2)
Institutional protocol for management of patients with MEN1	37 (64)
Patient volume <10	2/8 (25)
Patient volume 10–49	20/29 (69)
Patient volume 50–100	12/18 (67)
Patient volume >100	3/3 (100)
Research^b^	
Database (clinical data)	30 (52)
Patient volume <10	2/8 (25)
Patient volume 10–49	13/29 (45)
Patient volume 50–100	12/18 (67)
Patient volume >100	3/3 (100)
Biobank (specimen)	30 (52)
Patient volume <10	4/8 (50)
Patient volume 10–49	14/29 (48)
Patient volume 50–100	9/18 (50)
Patient volume >100	3/3 (100)
Both research database and biobank	21 (36)
Patient volume <10	1/8 (13)
Patient volume 10–49	11/29 (38)
Patient volume 50–100	6/18 (33)
Patient volume >100	3/3 (100)

Abbreviations: CoE, Center of Excellence; MEN1, Multiple Endocrine Neoplasia Type 1.

^a^ Including n = 1 both gastroenterologist and endocrinologist, *n* = 1 endocrine oncologist.

^b^ *n* = 5 unknown.

### 3.1 Organisation of care

In Q1‐Q2 2022, the volume of patients with MEN1 was <10 at eight (14%) CoEs, 10–49 at 29 (50%) CoEs, 50–100 at 18 (31%) CoEs and >100 at three (5%) CoEs (Table 1). In most CoEs, a single discipline provides the regular periodic screening and follow‐up of patients with MEN1, in most cases being the endocrinologist (Table 1). Endocrinology as the sole lead discipline for the regular screening and follow‐up was more often seen in CoEs with a patient volume of ≥50, namely 76% versus 54% in CoEs with a patient volume of <50 (*p* = 0.16). Larger centres more often have an institutional protocol for the management of patients with MEN1, and more often maintain a research database and/or biobank (Table 1).

### 3.2 Screening and surveillance of MEN1‐related NETs

#### 3.2.1 Biochemical screening

All CoEs screen patients for primary hyperparathyroidism (pHPT) with serum calcium measurement and, with the exception of three of the four Dutch CoEs, also with parathyroid hormone (PTH) (Table 2, Figure S1). Practices with regard to biochemical screening for duodenopancreatic NETs are heterogeneous, as can be seen in Table 2. Larger centres more often screen with fasting serum gastrin (FSG), glucagon and pancreatic polypeptide (PP). Additionally, some important variations among countries could be detected (Figure S1). Chromogranin A (CgA), glucagon, PP and vasoactive intestinal peptide (VIP) are more often determined in the United Kingdom (UK) (Figure S1) compared to other countries. Swiss CoEs also measure CgA, glucagon and PP more often. The majority of German CoEs also determine CgA. With regard to pituitary screening (Table 2), 93% of CoE screen with prolactin and 80% with insulin‐like growth factor 1 (IGF‐1). Additionally thyroid hormones are measured frequently. Sex hormones (including gonadotropins) and cortisol are measured less frequently as part of screening, CoEs with ≥50 patients less often than CoEs with <50 patients. No country‐specific pattern regarding pituitary biochemical screening was observed (Figure S1).

**TABLE 2:** Biochemical screening in asymptomatic adults with MEN1.

		Volume			Volume			
	Adults (*n* = 55 CoEs)	<50 (*n* = 34)	≥50 (*n* = 21)	*p*‐value	<10 (*n* = 5)	10–49 (*n* = 29)	50–100 (*n* = 18)	>100 (*n* = 3)
Primary hyperparathyroidism								
Calcium	55/55 (100%)	34/34 (100%)	21/21 (100%)	‐	5/5 (100%)	29/29 (100%)	18/18 (100%)	3/3 (100%)
PTH	52/55 (95%)	33/34 (97%)	19/21 (91%)	.55	5/5 (100%)	28/29 (97%)	17/18 (94%)	2/3 (67%)
Duodeno‐pancreatic Neuroendocrine Tumours								
FSG	45/55 (82%)	**24/34 (71%)**	**21/21 (100%)**	**<.01**	3/5 (60%)	21/29 (72%)	18/18 (100%)	3/3 (100%)
Insulin	26/55 (47%)	15/34 (44%)	11/21 (52%)	.59	3/5 (60%)	12/29 (41%)	9/18 (50%)	2/3 (67%)
Glucose	44/55 (80%)	27/34 (80%)	17/21 (81%)	1.00	3/5 (60%)	24/29 (83%)	14/18 (78%)	3/3 (100%)
CgA	38/55 (69%)	25/34 (74%)	13/21 (62%)	.39	3/5 (60%)	22/29 (76%)	11/18 (61%)	2/3 (67%)
Glucagon	24/55 (44%)	*11/34* (*32%*)	*13/21* (*62%*)	.*05*	2/5 (40%)	9/29 (31%)	11/18 (61%)	2/3 (67%)
PP	21/55 (38%)	**9/34 (27%)**	**12/21 (57%)**	.**04**	2/5 (40%)	7/29 (24%)	10/18 (56%)	2/3 (67%)
VIP	22/55 (40%)	11/34 (32%)	11/21 (52%)	.17	1/5 (20%)	10/29 (35%)	10/18 (56%)	1/3 (33%)
Pituitary								
Prolactin	51/55 (93%)	31/34 (91%)	20/21 (95%)	1.00	4/5 (80%)	27/29 (93%)	17/18 (94%)	3/3 (100%)
IGF‐1	44/55 (80%)	27/34 (79%)	17/21 (81%)	1.00	4/5 (80%)	23/29 (79%)	14/18 (78%)	3/3 (100%)
Sex hormones^a^	34/55 (62%)	24/34 (71%)	13/21 (48%)	.15	4/5 (80%)	20/29 (69%)	9/18 (50%)	1/3 (33%)
Thyroid Hormones	45/55 (82%)	28/34 (82%)	17/21 (81%)	1.00	4/5 (80%)	24/29 (83%)	15/18 (83%)	2/3 (67%)
Cortisol	30/55 (55%)	*22/34* (*65%*)	*8/21* (*38%*)	.*09*	4/5 (80%)	18/29 (62%)	8/18 (44%)	0/3 (0%)

Abbreviations: CgA, chromogranin A; CoE, Center of Excellence, FSG, fasting serum gastrin; IGF‐1, insulin‐like growth factor 1; PP, pancreatic polypeptide; PTH, parathyroid hormone; VIP, vasoactive intestinal peptide.

^a^ Sex hormones: Males: testosterone, in 94% combined with gonadotropins Females: E2, in 97% combined with gonadotropins.

#### 3.2.2 Gastrinoma diagnosis and esophagogastroduodenoscopy (EGD)

Nearly all CoEs use FSG for diagnosing gastrinoma, in 55% of cases combined with gastric pH and less often other tests (Table 3). The secretin stimulation test is used by 10 CoEs from 8 different countries (Figure S2H). No differences are seen based on MEN1 patient volume (Table 3) or country (Figure S2). Most reported use of EGD is for regular screening/surveillance in patients with hypergastrinemia or for specific indications (Table 3). Larger volume CoEs perform routine EGD in patients with hypergastrinemia more often, but this was not significant (Table 3). Gastric pH is more often determined in some countries including the UK and Germany (Figure S2). In Italy, many CoEs use the secretin stimulation test in contrast to other countries (Figure S2).

**TABLE 3:** Screening and Surveillance Practices in adults with MEN1.

	*N* (%)	<50	≥50	*p*‐value	<10	10–49	50–100	>100
Biochemical modalities used for gastrinoma diagnosis (*n* = 55)								
FSG alone	20/55 (36)	12/34 (35)	8/21 (38)	.83	2/5 (40)	10/29 (35)	6/18 (33)	2/3 (67)
FSG + gastric pH +/− other tests^a^	30/55 (55)	18/34 (53)	12/21 (57)	.76	3/5 (60)	15/29 (52)	11/18 (61)	1/3 (33)
FSG + calcium or secretin stimulation test	4/55 (7)	3/34 (9%)	1/21 (5)	‐	0/5	3/29 (10)	1/18 (6)	0/3
Secretin stimulation test only	1/55 (2)	1/34	0/21	‐	0/5	1/29	0/18	0/3
Multiple answers possible								
FSG	54/55 (98%)	33/34 (97)	21/21 (100)	‐	5/5	28/29 (96)	18/18	3/3
Gastric pH	30/55 (24%)	18/34 (53)	12/21 (57)	.76	3/5 (60)	15/29 (52)	11/18 (61)	1/3 (33)
Secretin stimulation test	17/55 (31%)	12/34 (35)	5/21 (24)	.37	2/5 (40)	10/29 (35)	5/18 (28)	0/3
Basal Acid Output	4/55 (7%)	3/34 (9)	1/21 (5)	‐	0/5	3/29 (10)	1/18 (6)	0/3
Calcium stimulation test	10/55 (18%)	7/34 (21)	3/21 (14)	.73	1/5 (20)	6/29 (21)	3/18 (17)	0/3
EGD routinely performed (*n* = 54)				.33				
No	18/54 (32)	13/34 (38)	5/20 (25)		3/6 (50)	10/28 (36)	4/17 (24)	1/3 (33)
Yes, in all MEN1 pts	8/54 (14)	6/64 (18)	2/20 (10)		1/6 (17)	5/28 (18)	2/17 (12)	0/3
To screen for dNETs	7							
								
To screen for gNETs	7							
To screen for PUD	5							
Yes, in pts. with elevated FSG	2/54 (52)	15/34 (44)	13/20 (65)		2/6 (33)	13/28 (46)	11/17 (65)	2/3 (67)
To screen for dNETs	24							
Surveillance of dNETs	22							
To screen for gNETs	25							
Surveillance of gNETs	25							
To monitor PUD	4							
To fine‐tune PPI	4							
Use of EUS in screening/surveillance of PanNETs (*n* = 56)—multiple answers possible								
Not available	1/56 (2)	1/35 (3)	0/21	‐	0/6	1/29 (3)	0/18	0/3
Not used	1/56 (2)	1/35 (3)	0/21	‐	1/6 (17)	0/29	0/18	0/3
Regular screening in pt. w/o PanNET	15/56 (27)	11/35 (31)	4/21 (19)	.31	3/6 (50)	8/29 (28)	3/18 (17)	1/3 (33)
Regular surveillance in pt. w PanNET	29/56 (52)	19/35 (54)	10/21 (48)	.63	3/6 (50)	16/29 (55)	9/18 (50)	1/3 (33)
On specific indication	29/56 (52)	17/35 (49)	12/21 (57)	.53	2/6 (33)	15/29 (52)	10/18 (56)	3/3 (100)
Use of FNA/FNB (*n* = 54)				.13				
Generally at every EUS	8 (15%)	7/33 (21)	1/21 (5)		1/5 (20)	6/28 (21)	1/18 (6)	0/3
On specific indication only	45 (83%)	26/33 (79)	20/21 (95)		1/5 (80)	22/28 (79)	17/18 (94)	3/3 (100)
Use of ^68^Ga‐DOTA‐peptides‐PET/CT (*n* = 55)—multiple answers possible								
Not used	3/55 (5)	2/34 (6)	1/21 (5)	‐	0/6	2/28 (7)	1/18 (6)	0/3
Regular use in								
Surveillance metastatic NETs	37/55 (67)	24/34 (71)	13/21 (62)	.56	4/6 (67)	20/28 (71)	10/18 (56)	3/3 (100)
Surveillance non‐metastatic NETs	23/55 (42)	12/34 (35)	11/21 (52)	.21	3/6 (50)	9/28 (32)	10/18 (56)	1/3 (33)
Screening in pt. w/o NETs	13/55 (24)	8/34 (24)	5/21 (24)	.98	3/6 (50)	5/28 (18)	5/18 (28)	0/3 (100)
On specific indication	19/55 (35)	11/34 (32)	8/21 (38)	.77	2/6 (33)	9/28 (32)	6/18 (33)	2/3 (67)
Preferred modality for surveillance of small PanNETs (*n* = 56)—multiple answers possible								
MRI	52/56 (93)	31/35 (87)	21/21 (100)	.29	4/6 (67)	27/29 (93)	18/18 (100)	3/3 (100)
EUS	20/56 (36)	12/35 (34)	8/21 (38)	.77	1/6 (17)	11/29 (38)	7/18 (33)	1/2 (33)
CT	20/56 (36)	11/35 (31)	9/21 (43)	.39	2/6 (33)	9/29 (31)	7/18 (39)	2/3 (67)
^68^Ga‐DOTA‐peptides‐PET/CT	19/56 (34)	12/35 (34)	7/21 (33)	.94	2/6 (33)	10/29 (35)	7/18 (39)	0/3
Surveillance interval in small PanNETs (*n* = 56)								
Every six months	11/56 (20)	6/35 (17)	5/21 (24)		2/6 (33)	4/29 (14)	5/18 (28)	0/3
Initially 6 months, then longer if continuously stable	31/56 (55)	19/35 (54)	12/21 (57)		4/6 (67)	15/29 (52)	10/18 (56)	2/3 (67)
*6 months, then yearly*	*15/56* (*27*)	*10/35* (*29*)	*5/21* (*24*)		*1/6* (*17*)	*9/29* (*31*)	*3/18* (*17*)	*2/3* (*67*)
*6 months, then yearly or longer*	*16/56* (*28*)	*9/35* (*26*)	*7/21* (*33*)		*3/6* (*50*)	*6/29* (*21*)	*7/18* (*39*)	*0/3*
Yearly	13 (23)	9/35 (26)	4/21 (19)		0/6	9/29 (31)	3/18 (17)	1/3 (33)
Every 2 year	1 (2)	1/35 (3)	0/21		0/6	1/29 (3)	0/18	0/3
Preferred modality thoracic screening/surveillance (*n* = 56)—multiple answers possible								
CT	50/56 (89)	31/35 (89)	19/21 (91)	1	5/6 (83)	26/29 (90)	16/18 (89)	3/3 (100)
^68^Ga‐DOTA‐peptides‐PET/CT	18/56 (32)	9/35 (26)	9/21 (43)	.18	1/6 (17)	8/29 (28)	9/18 (50)	0/3
MRI	13/56 (23)	10/35 (29)	3/21 (14)	.33	2/6 (33)	8/29 (28)	3/18 (17)	0/3
Screening interval for thoracic NETs								
Yearly	12/56 (21)	9/35 (26)	3/21 (14)		2/6 (17)	7/29 (24)	3/18 (17)	0
Every 2 years	13/56 (23)	10/35 (29)	3/21 (14)		3/6 (50)	7/29 (24)	3/18 (17)	0
Every 3 years	22/56 (39)	10/35 (29)	12/21 (57)		1/6 (17)	9/29 (31)	9/18 (50)	3/3 (100)
Every 5 years	5/56 (9)	4/35 (11)	1/21 (5)		0	4/29 (14)	1/18 (6)	0
Other^b^	3/56 (5)	1/35 (3)	2/21 (10)		0	1/29 (3)	2/18 (11)	0
Unknown	1/56 (2)	1/35 (3)	0		0	1/29 (3)	0	0
Surveillance interval for lung NETs								
Never observe lung NETs	7 (13%)	5/35 (14)	2/21 (10)		0	5/29 (17)	2/18 (11)	0
Every 6 months	6 (11%)	5/35 (14)	1/21 (5)		1/6 (17)	4/29 (14)	1/18 (6)	0
Initially 6 months, then longer if continuously stable	34 (61%)	18/35 (51)	16/21 (76)		3/6 (50)	15/29 (52)	13/18 (72)	3/3 (100)
*6 months, then yearly*	*15*	*7/35* (*20*)	*8/21* (*38*)		*0*	*7/29* (*24*)	*6/18* (*33*)	*2/3* (*67*)
*6 months, then yearly or longer*	*19*	*11/35* (*31*)	*8/21* (*38*)		*3/6* (*50*)	*8/29* (*28*)	*7/18* (*39*)	*1/3* (*33*)
Yearly	8 (14%)	6/35 (17)	2/21 (10)		2/6 (33)	4/29 (14)	2/18 (11)	0
Unknown	1 (2%)	1/35 (3)	0		0	1/29 (3)	0	0

*Note*: Bold indicates statistically significant difference. Italic indicates a trend (*p*‐value between 0.05 and 0.1).

Abbreviations: CT, computed tomography; dNETs, duodenal neuroendocrine tumours; EGD esophagogastroduodenoscopy; EUS, endoscopic ultrasound; FNA, fine‐needle aspiration; FNB, fine‐needle biopsy; FSG, fasting serum gastrin; gNETs, gastric neuroendocrine tumours; MRI magnetic resonance imaging; PanNETs, pancreatic neuroendocrine tumours; PET/CT, positron emission tomography/computed tomography; PPI, proton pump inhibitor; PUD, peptic ulcer disease; w, with; w/o, without.

^a^ Secretin stimulation test, calcium stimulation test or basal acid output.

^b^ Age‐dependent varying between 1 and 3 years (1), once around 30 and then 5–10 years later (1), dependent on clinical characteristics (1).

#### 3.2.3 EUS and EUS‐FNA in screening for pancreatic NETs

The most reported use of endoscopic ultrasonography (EUS) is for specific indications (Tables 3 and 4) and in the regular surveillance of existing pancreatic NETs (PanNETs) (Table 3). Only 28% of CoEs routinely use EUS to screen for PanNETs in patients without known tumours. No significant association with patient volume was observed. The routine use of EUS in screening and surveillance was much more common in Germany and Switzerland, mainly for surveillance, whereas the Netherlands and the UK more often use EUS for specific indications only (Figure S3). Indications for EUS mentioned by the CoEs were, in decreasing order of frequency, identification or monitoring of functional tumours/ZES, to perform Fine‐needle aspiration or biopsy (FNA/FNB), in case of diagnostic uncertainty, for establishing NET diagnosis, for suspicious lesions and pre‐operative assessment (Table 4).

**TABLE 4:** Reported indications for particular diagnostic imaging.

Indications for the use of endoscopic ultrasound (answers in order of decreasing frequency)
*Functional Tumours*: (1) Identify origin of functional duodenal/pancreatic NETs and/or (2) identify origin of elevated biochemical markers (e.g. CgA) and/or (3) ZES—monitoring of NETs difficult to seen on MRI/detection NETs not seen on MRI
In order to perform *FNA/B* (e.g. for diagnosis/grading)
*Diagnostic uncertainty*: Unclear pancreatic or duodenal lesion/doubt about diagnosis of PanNET/lesion visible on PET/CT but not on conventional imaging
*Diagnostic*: (1) for diagnosis (2) confirming diagnosis of a (new) pancreatic/duodenal or gastric NET on other imaging
*Suspicious Features*: Larger, growing or changing PanNETs
*Pre‐operatively*: (1) assessing pancreatic duct (2) define volume of disease/precise evaluation of disease (3) confirm PanNETs if not done by other means such as 68Gallium‐DOTA‐PET/CT
Indications to perform FNA/FNB (answers in order of decreasing frequency)
*Grading and classification*: (1) at initial diagnosis (2) in large lesions, changing lesions or lesions with (unusual) growth
*Diagnostic uncertainty*: e.g. negative on functional imaging, suspicious of PDAC
*Functional tumour*: Identifying origin of a specific hormone secretion
*Therapeutic choice*: if histology would change management/classification needed for therapy
*Metastases*: Abnormal lymph nodes or liver lesions in a patient with a PanNET
Indication to perform ^68^Gallium‐DOTA‐PET/CT (answers in order of decreasing frequency)
*Therapy planning*: (1) suitability for PRRT
*Staging*: (1) at initial diagnosis (2) at change or suspected progression (3) PanNETs 1–2 cm in size (4) evaluation before planned surgery to determine extend of disease/ occult metastases
*Confirmation of NET diagnosis*
*Functional syndrome* or elevated gut hormones w/o visible primary
Surveillance in patients with *disease only visible on* ^*68*^ *Gallium‐DOTA‐PET/CT*
Surveillance *after resection*
In patients with a family history of *thymic NETs*

Abbreviations: CgA chromogranin A; FNA/B fine‐needle aspiration/biopsy; MRI magnetic resonance imaging; NET neuroendocrine tumour; PanNETs pancreatic neuroendocrine tumours; PDAC pancreatic ductal adenocarcinoma; PET‐CT positron emission tomography‐ computed tomography; ZES Zollinger‐Ellison Syndrome.

FNA/FNB of a pancreatic lesion in patients with MEN1 is in most CoEs performed for specific reasons, such as grading/classification in lesions with unusual behaviour, clarifying diagnostic uncertainty and diagnosing metastases, but not done routinely (Tables 3 and 4). Seven of the eight CoEs that indicated performing FNA/FNB routinely had a patient volume of <50 (Table 2). No country‐specific differences were seen (Figure S3D).

#### 3.2.4 Use of PET/CT

The most reported use of ^68^Gallium‐DOTA‐peptides‐PET/CT was for regular follow‐up of metastatic NETs (67%), followed by regular surveillance of non‐metastatic NETs (42%) and for specific indications (35%) such as pre‐surgical evaluation, staging and therapy planning (Tables 3 and 4). In 24% of CoEs, ^68^Gallium‐DOTA‐peptides‐PET/CT was used as a screening tool. No patient volume‐ or country‐specific differences among the CoEs were observed (Table 3 and Figure S4). Of the 23 CoEs that use ^68^Gallium‐DOTA‐peptides‐PET/CT for regular surveillance in non‐metastatic NETs, 8/23 (35%) would perform the scan yearly, 12/23 (52%) every two years, 2/23 (9%) every three years and 1/23 (4%) indicated using it on an individualised basis.

Regarding the newly introduced but less readily available ^111^In‐Exendin‐PET/CT, 7/56 (12.5%) CoEs have the ability to perform it in‐house and use it specifically for MEN1‐related insulinoma. A significant number of CoE, 17/56 (30%), do not have the ability to perform this investigation in‐house, but refer to other centres if necessary, whereas 32/56 (57%) CoEs do not have access to ^111^In‐Exendin‐PET/CT.

#### 3.2.5 Pancreatic NETs surveillance

For the surveillance of small PanNETs, magnetic resonance imaging (MRI) is the backbone investigation with 52/56 CoEs (93%) indicating this as (one of) the preferred modality(ies) (Table 3). Seventeen CoEs (30%) use MRI alone, whereas the others use this in various combinations with EUS, CT and ^68^Gallium‐DOTA‐peptides‐PET/CT. No patient volume‐specific differences were observed among the CoEs. However, for the countries where there were data available from ≥3 CoEs, a country‐specific trend was observed (Figure S5). In the UK, in addition to MRI, CT was more often used, whereas German CoEs used EUS more often. CoEs in Italy and Germany more often used ^68^Gallium‐DOTA‐peptides‐PET/CT, compared to the Netherlands, Switzerland and the UK. The imaging frequency for small (≤20 mm) PanNETs under surveillance differed among CoEs; in particular, most (55%) would advise initial imaging after 6 months and then yearly or longer if the lesions remain stable on sequential imaging (Table 3). No differences in patient volume‐ or country‐specific practice were observed (Table 3 and Figure S5).

#### 3.2.6 Screening and surveillance for thoracic (lung and thymic) NETs

Regarding periodic screening for thoracic NETs, CT is the primary imaging modality, with 50/56 (89%) CoEs using it for thoracic screening either alone (50%) or combined (39%) with MRI and/or ^68^Gallium‐DOTA‐peptides‐PET/CT (Table 3). Larger patient volume centres seemed to use ^68^Gallium‐DOTA‐peptides‐PET/CT more often and MRI less, although this was not significant (Table 3). MRI was more often used in the UK and Switzerland, whereas in the Netherlands, Italy, and Germany, ^68^Gallium‐DOTA‐peptides‐PET/CT was more often used (Figure S6). There was a wide spectrum of advised screening intervals for patients without known thoracic NETs, generally ranging from every 1 to every 3 years (Table 3). Similarly, the recommended frequency of surveillance in small or suspected lung NETs differed, although most CoEs would recommend initial imaging after 6 months and then extend subsequent imaging intervals to yearly or longer if lesions remain continuously stable. In CoEs with a large patient volume, practices were more homogeneous and follow‐up intervals tended to be longer compared to low volume centres (Table 3); no country‐specific differences were observed (Figure S6).

### 3.3 Patients with genotype‐negative clinical MEN1

Genotype‐negative ‘clinical’ MEN1 (GN‐MEN1) are patients with 2 of the 3 main manifestations without a detectable (likely) pathogenic variant in the *MEN1* gene or other relevant genes (Table 5). For these patients, the majority of CoEs (39/57; 68%) would advise periodic screening similar to mutation‐positive patients. Four CoEs with a patient volume of <50 would advise no specific MEN1‐related follow‐up. For the other 14 CoEs (25%), the advice would depend on the clinical characteristics of the patients. The combination of organs involved, and age of the patients were the most important factors to guide further decisions, while the majority also considered the presence of a positive family history. Regarding combination of manifestations, almost all CoEs would advise periodic screening similar to mutation‐positive patients in GN‐MEN1 patients with duodenopancreatic NETs, while a minority would advise so in patients with pHPT and pituitary adenomas. Regarding age, most CoEs would advise periodic screening similar to mutation‐positive patients in GN‐MEN1 below 50 years of age (Table 5).

**TABLE 5:** Periodic screening in patients with genotype‐negative clinical MEN1.

		Volume		Volume			
	*N* (%)	<50	≥50	<10	10–49	50–100	>100
Advise for periodic screening for patients with 2/3 main manifestations, negative genetic testing and negative family history							
No specific MEN1‐related follow‐up	4/57 (7)	4/36 (11)	0/21	3/7 (43)	1/29 (3)	0/18	0/3
Similar to mutation positive	39/57 (68)	22/36 (61)	17/21 (81)	4/7 (57)	18/29 (62)	15/18 (83)	2/3 (67)
Depends on clinical characteristics	14/57 (25)	10/36 (28)	4/21 (19)	0/7	10/29 (34)	3/18 (17)	1/3 (33)
If dependent on clinical characteristics this depends on…—multiple answers possible							
Age	11/14 (78)	7/10 (70)	4/4 (100)		7/10 (70)	3/3 (100)	1/1
Combination of manifestations	12/14 (86)	9/10 (90)	3/4 (75)		9/10 (90)	2/3 (67)	1/1
Family History	8/14 (57)	5/10 (50)	3/4 (75)		5/40 (50)	2/3 (67)	1/1
Not specified	1/14 (7)	1/10 (10)	‐		1/10 (10)	‐	‐
For which combination of manifestations would periodic screening similar to mutation positive patients be advised?							
pHPT and PA	3/12 (25)	2/10 (20)	1/4 (25)		2/10 (20)	1/3 (33)	0/1
pHPT and duodeno‐pancreatic NET	11/12 (92)	8/10 (80)	3/4 (75)		8/10 (80)	2/3 (67)	1/1
PA and duodeno‐pancreatic NET	11/12 (92)	8/10 (80)	3/4 (75)		8/10 (80)	2/3 (67)	1/1
At which age cut‐off would periodic screening similar to mutation positive patients be advised?							
Below the age of 30	3/11 (27)	1/7 (14)	2/4 (50)		1/7 (14)	1/3 (33)	1/1
Below the age of 50	7/11 (64)	5/7 (71)	2/4 (50)		5/7 (71)	2/3 (67)	
Unable to answer	1/11 (9)	1/7 (14)	‐		1/7 (14)	‐	
Periodic screening advised for a *first‐degree‐relative* of a patient with 2/3 main manifestations, negative genetic testing and negative family history							
No	16/57 (28)	12/36 (33)	4/21 (19)	2/7 (29)	10/29 (35)	3/18 (17)	1/3 (33)
Yes	24/57 (42)	17/36 (15)	7/21 (33)	4/7 (57)	13/29 (45)	7/18 (39)	‐
Other	12/57 (21)	5/36 (14)	7/21 (33)	1/7 (14)	4/29 (14)	5/18 (28)	2/3 (67)
Unable to answer	5/57 (9)	2/36 (6)	3/21 (14)	‐	2/29 (7)	3/18 (17)	‐

Abbreviations: NET, neuroendocrine tumour; PA, pituitary adenoma; pHPT, primary hyperparathyroidism.

For a first‐degree relative of a GN‐MEN1 patient, 42% of CoEs would advise periodic screening, 28% would not, and 21% indicated giving other advice, ranging from education to periodic checking of calcium levels, to an initial full screening with follow‐up depending on the outcome. Most of these CoEs indicated this would depend on the characteristics of the index patient. Larger patient volume centres more often opted for the ‘other’ option, favouring a more individualised approach. When looking at countries from which data from ≥3 CoEs were available, the Netherlands was most limited in the periodic screening of GN‐MEN1 patients, with three out of four CoEs indicating periodic screening would depend on the clinical characteristics of the patient and no periodic follow‐up would be advised for 1st degree family members (Figure S7).

## 4 DISCUSSION

The results, based on responses from 86% of CoEs, show marked heterogeneity in screening and surveillance practices for MEN1, but also common trends. This heterogeneity is not surprising. Screening and surveillance for MEN1‐related tumours improves outcomes,^5^, ^14^, ^15^, ^16^ but the evidence of many individual elements is limited. The scarcity of validated risk factors precludes uniform risk‐based strategies,^17^ although screening and surveillance should ideally be individualized. Timing and type of intervention for many MEN1‐related NETs is still a manner of debate. There is limited evidence regarding costs and adverse effects of elements of screening. Additionally, resources and reimbursement are practical reasons for heterogeneity. Finally, as the knowledge of the natural history of MEN1‐related NETs increases and new therapies are being investigated,^18^ screening and surveillance practices should be continuously redefined and reevaluated. Mapping of current practices is an important starting point for these endeavours.

Presently, imaging is the cornerstone of PanNET screening in MEN1. The role of biomarkers in screening for NF‐PanNETs is questionable and reflected in the heterogeneous practices among CoEs. Well‐conducted cohort studies show that CgA, PP and glucagon have low diagnostic accuracy for the presence of a PanNET in MEN1, making them unsuitable as screening tools.^19^, ^20^ Local factors may contribute to the implementation of these findings. In Dutch CoEs—which participated in one of the aforementioned studies^19^—CgA, glucagon and PP are not used in screening, whereas all UK CoEs—where NET markers are available as one single laboratory order—measure these hormones.

For gastrinomas, usually small, multiple and duodenal in MEN1, conventional imaging has very low sensitivity,^21^ and biochemical screening is the cornerstone. Screening with FSG is a uniform practice in all CoEs with a patient volume >50. Diagnosing gastrinomas in MEN1 may be challenging with early detection through screening leading to milder phenotypes and widespread PPI use and risk of cessation make obtaining gastric pH off PPI difficult.^22^ This is reflected in the practices at the CoEs where 55% use FSG in combination with gastric pH for the biochemical diagnosis of gastrinoma.

EUS is the most sensitive imaging for small PanNET,^23^ however as small NF‐panNETs are generally indolent^23^ and intervention is not advised until they reach 20 mm or demonstrate rapid growth,^11^ its role in screening is debated. Presently, CoEs use EUS most often for routine surveillance of known PanNETs and for specific indications where EUS can have direct implications for management as can been seen in Table 4. International experts agree that FNA/FNB should only be performed in patients with MEN1 when the outcome of this invasive procedure has important management consequences, not routinely at EUS.^11^

SST‐PET/CT has a superior sensitivity for the detection of PanNETs and gastrinomas as well as locoregional and distant metastases in patients with MEN1 compared with conventional imaging.^24^, ^25^, ^26^, ^27^, ^28^ The debate therefore is not *if* SST‐PET/CT has a place in screening and surveillance of patients with MEN1, but *when*. As a consequence, current practice in CoEs is diverse. Outside the metastasized setting, SST‐PET/CT will most likely contribute most for pre‐operative staging of NETs, surveillance of progressive gastrinomas and PanNETs when outcome would have direct therapeutic consequences and in the diagnosis and staging of gastrinomas. The use in screening for duodenopancreatic NETs (i.e., in patients without suspicion of a functional NET and without lesions on conventional imaging) is highly questionable, as additionally detected lesions in this setting are almost always without clinical consequences.

Given the usually indolent behaviour (growth rate of 0.1–1.32 mm/yr.^23^) and very rare metastatic spread of small NF‐PanNETs, several groups advise a screening interval of 2–3 years if initial (conventional) imaging is negative and to individualise surveillance intervals based on tumour growth with initial repeat imaging after 6–12 months and then yearly or even longer if the PanNET(s) remain stable,^3^, ^10^, ^11^ a practice seen in over half of the CoEs in our study.

As lungNETs are more frequent in MEN1 than originally thought, but generally have an indolent course with infrequent metastases, active surveillance now seems a viable strategy for small sized tumours, as is also recommended in the European Society of Medical Oncology (ESMO) guidelines.^29^, ^30^, ^31^ In the present survey, 13% of CoEs never use a watchful waiting strategy, while in those that do so, surveillance intervals vary, with longer intervals seen in large patient‐volume centres.

With regards to GN‐MEN1, data from independent cohorts show that these patients have a different course of disease with higher age of onset of the first manifestation, rarely develop additional manifestations of MEN1 and have a life‐expectancy similar to the general population.^1^, ^12^ Most of these patients meet diagnostic criteria because of having pHPT with a pituitary adenoma and pHPT in this setting is often single gland disease.^1^, ^12^ The authors of these papers advocate for a more limited screening and surveillance strategy in this setting, based on clinical characteristics.^1^, ^12^, ^32^ Currently, this practice is still limited in CoEs.

This study is the first to systematically map screening and surveillance practices for MEN1‐related NETs among centres of expertise. The high response rates make the outcomes truly representative of ENETS CoE practices.

Even so, a significant number of European MEN1 patients are most likely followed outside ENETS CoEs, e.g. in other European (such as Endo‐ERN^33^) or national centres of expertise, or outside centres of expertise. Hence, in most centres only a limited number of patients with MEN1 is seen. Even in ENETS CoEs less than half see more than 50 patients with MEN1. The importance of volume lies therein that through large numbers, the multidisciplinary team gains experience with the full scope of the disease allowing tailored management. Care for MEN1 is by nature complex, individualized, multidisciplinary and multimodal. This cannot be fully captured in a survey like the present. Firm conclusions regarding the association of practices with volume or country were also difficult due to the sample size, the limited number of countries with ≥3 CoEs responding and the inability to adjust for country when looking at volume and vice‐versa. Finally, this study did not investigate outcomes related to different screening and surveillance strategies. However, within these limitations, the present study provides an important overview of practices for screening and surveillance in NET centres of expertise in Europe as a baseline and reference for further studies.

As this study highlights, there remain many open questions regarding screening and surveillance of MEN1‐related NETs. For example, there is an urgent need for improved risk stratification of pan‐ and lung NETs to individualize screening, surveillance and interventions, for novel biomarkers for early detection of thymicNETs, for improved diagnosis and risk assessment in gastrinomas and for more clarity on the place of SST‐PET/CT. These are but some of the open questions and challenges. Answers are going to be found only by multicentre and multidisciplinary collaboration. Retrospective multicentre studies remain valuable for example to develop dynamic risk prediction models using all currently available clinical, imaging, genetic and molecular data, which can then be prospectively validated. As the MEN1 phenotype changes, natural history studies in large, unselected populations remain necessary. For specific questions, prospective studies should be set up, including pragmatic trials where feasible, for example for the role of SST‐PET/CT. Challenges for setting up these studies include the low event rates and the long time to outcome. New minimal invasive biomarkers for early detection and risk stratification should be developed through collaboration between basic and translational scientists and clinicians. Paramount to all these endeavours is the ability to study well‐characterized patient cohorts with availability of biospecimens at key points in the history of the disease.

## AUTHOR CONTRIBUTIONS

**Carolina R. C. Pieterman:** Conceptualization; writing – original draft; writing – review and editing; formal analysis; data curation; investigation; methodology; project administration; resources. **Simona Grozinsky‐Glasberg:** Conceptualization; project administration; data curation; resources; writing – review and editing. **Dermot O'Toole:** Conceptualization; project administration; data curation; resources; writing – review and editing. **James R. Howe:** Conceptualization; project administration; data curation; resources; writing – review and editing. **Valentina Ambrosini:** Conceptualization; resources; writing – review and editing. **Susana H. Belli:** Conceptualization; resources; writing – review and editing. **Mikkel Andreassen:** Resources; writing – review and editing. **Nehara Begum:** Conceptualization; writing – review and editing; resources. **Timm Denecke:** Conceptualization; writing – review and editing; resources. **Antongiulio Faggiano:** Conceptualization; resources; writing – review and editing. **Massimo Falconi:** Conceptualization; writing – review and editing; resources. **Jo Grey:** Writing – review and editing; conceptualization. **Ulrich P. Knigge:** Conceptualization; writing – review and editing; resources. **Teodora Kolarova:** Conceptualization; writing – review and editing. **Bruno Niederle:** Conceptualization; writing – review and editing; resources. **Els Nieveen van Dijkum:** Conceptualization; writing – review and editing; resources. **Stefano Partelli:** Conceptualization; writing – review and editing; resources. **Andreas Pascher:** Conceptualization; writing – review and editing; resources. **Guido Rindi:** Conceptualization; writing – review and editing; resources. **Philippe Ruszniewski:** Conceptualization; writing – review and editing; resources. **Stefan Stättner:** Conceptualization; resources; writing – review and editing. **Timon Vandamme:** Conceptualization; writing – review and editing; resources. **Juan W. Valle:** Conceptualization; writing – review and editing; resources. **Marie‐Pierre Vullierme:** Writing – review and editing; resources; conceptualization. **Staffan Welin:** Conceptualization; writing – review and editing; resources. **Aurel Perren:** Conceptualization; project administration; data curation; resources; writing – review and editing. **Detlef K. Bartsch:** Conceptualization; resources; project administration; data curation; writing – review and editing. **Gregory K. Kaltsas:** Conceptualization; project administration; resources; supervision; writing – review and editing. **Gerlof D. Valk:** Conceptualization; project administration; supervision; resources; writing – review and editing; writing – original draft; formal analysis; methodology.

## CONFLICT OF INTEREST STATEMENT

Dermot O'Toole reports previous educational grants from Novartis, Ipsen, Wyeth Leferle and AstraZenca. Valentina Ambrosini reports personal fees from AAA outside the submitted work. Guido Rindi reports past activities for AAA speakers bureau and Bracco Imaging Suisse, consultant. The other authors have no conflict of interest to declare.

### PEER REVIEW

The peer review history for this article is available at https://www.webofscience.com/api/gateway/wos/peer-review/10.1111/jne.13468.

## Supporting information


**Data S1.** Supporting Information Figures.

**Data S2.** Supporting Information.

## Data Availability

The data that support the findings of this study are available on request from the corresponding author. The data are not publicly available due to privacy or ethical restrictions.
