# Supplementary material for: Screening and surveillance practices for Multiple Endocrine Neoplasia type 1‐related Neuroendocrine Tumours in European Neuroendocrine Tumor Society Centers of Excellence (ENETS CoE)—An ENETS MEN1 task force questionnaire study
Source: J Neuroendocrinol. 2024 Nov 26;37(1):e13468. doi: 10.1111/jne.13468 (PMC11750319; doi:10.1111/jne.13468)

# Supplemental Figures

# Supplemental Figure 1

Biochemical screening of asymptomatic adults with MEN1 by country – for countries from which 3 or more CoEs provided answers

Supplemental Figure 1A. Adult - PTH included in biochemical screening of asymptomatic adults with MEN1

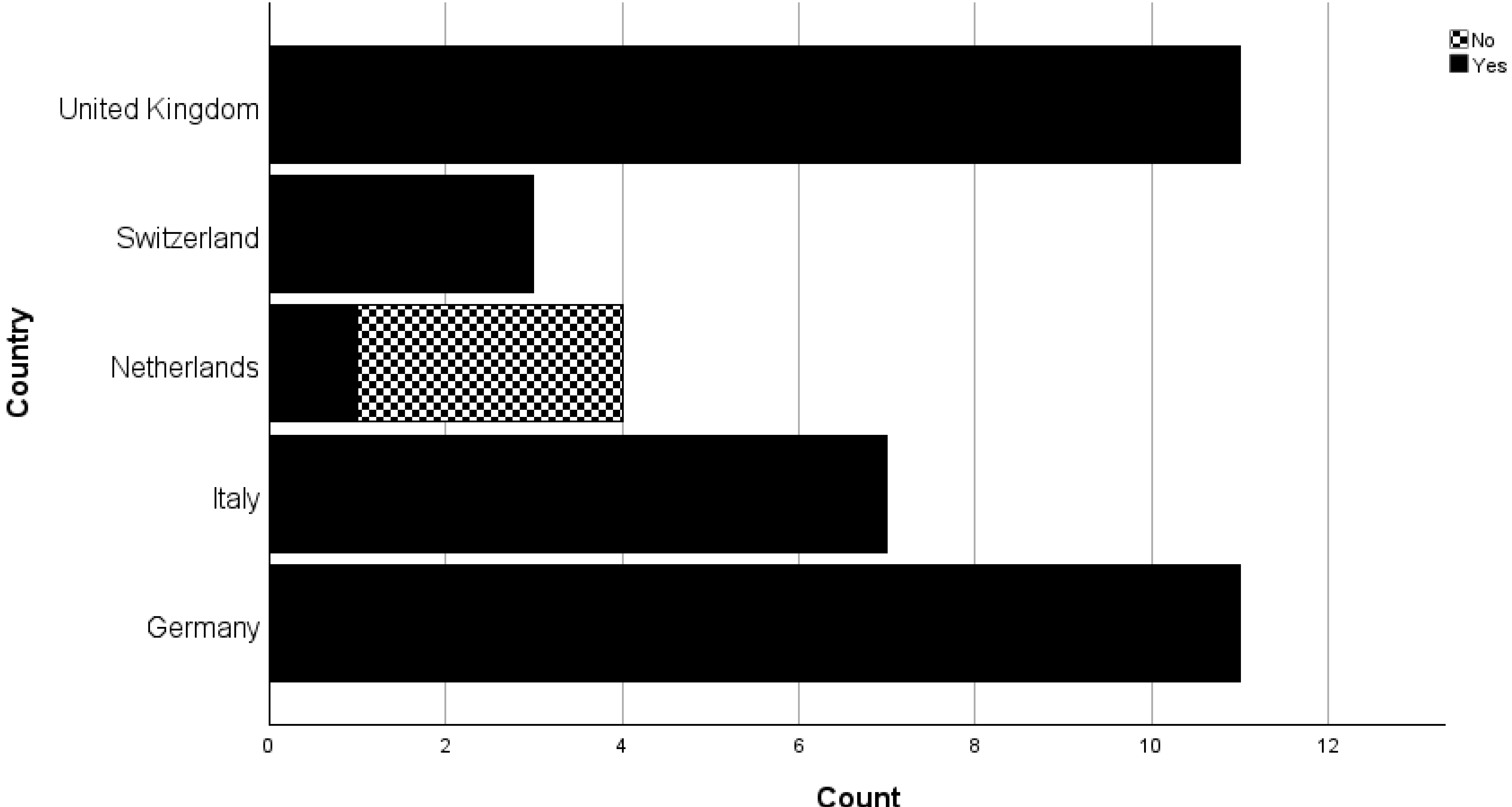

## Supplemental Figure 1B - Adult - Fasting Serum Gastrin included in biochemical screening of asymptomatic adults with MEN1

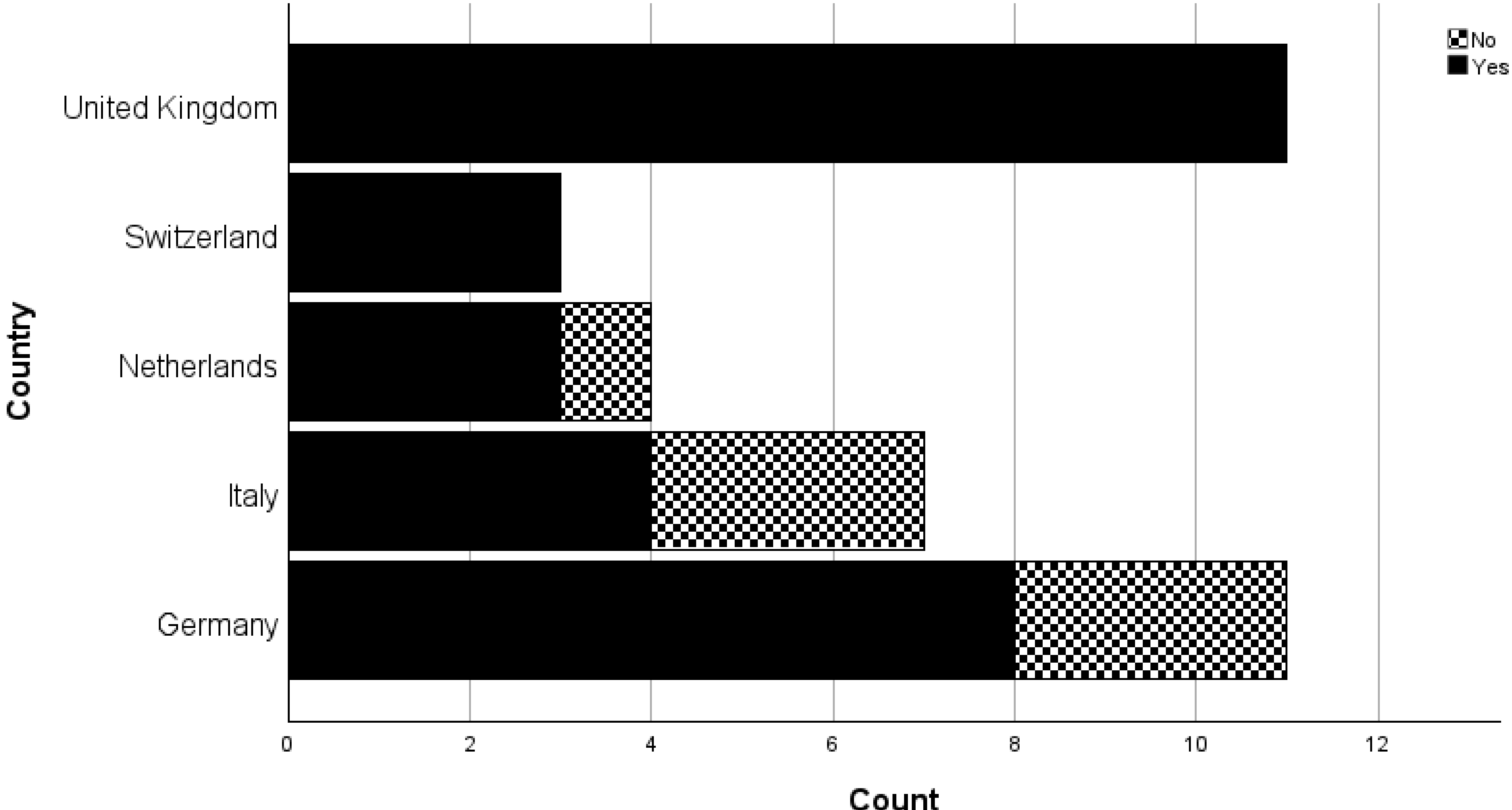

Supplemental Figure 1D - Adult - Fasting Glucose included in biochemical screening of asymptomatic adults

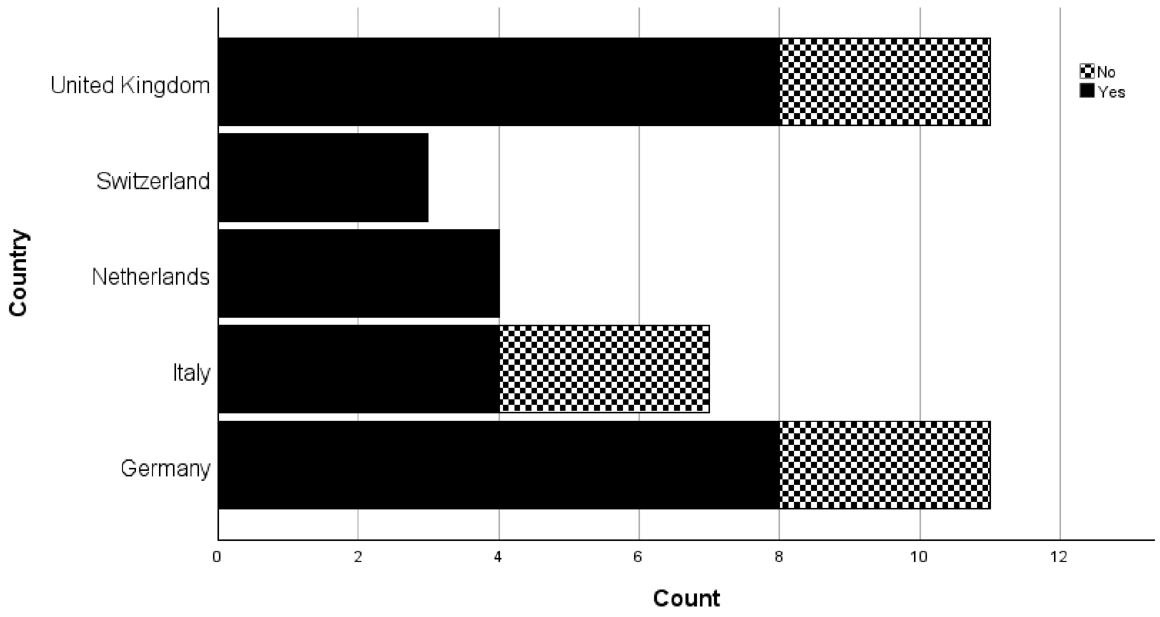


Supplemental Figure 1C - Adult - Fasting insulin included in biochemical screening of asymptomatic adults with MEN1

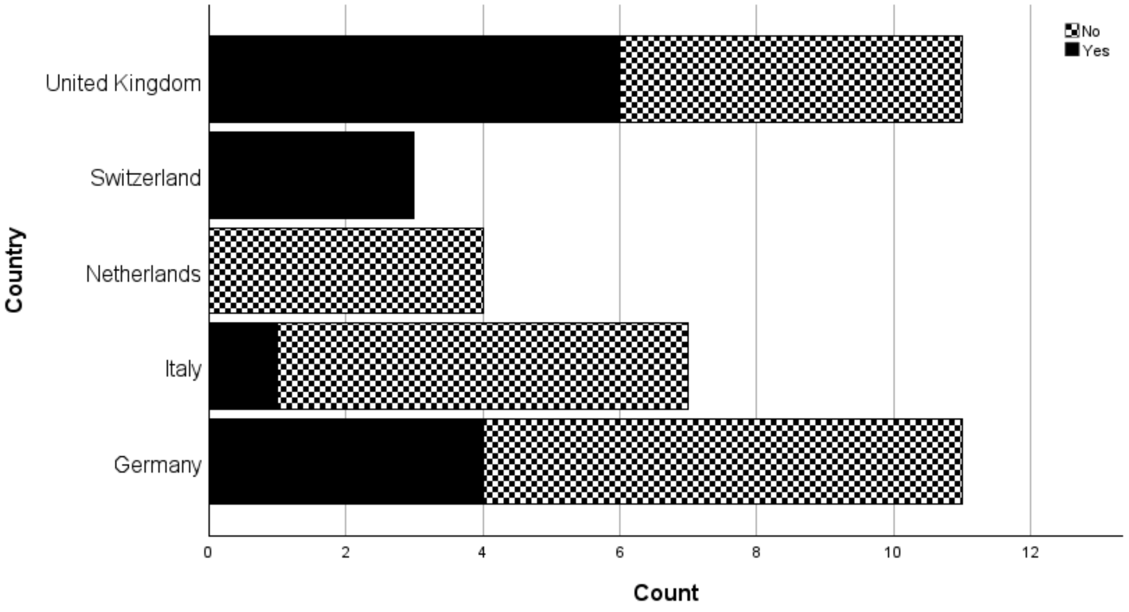


Supplemental Figure 1E - Adult - Biochemical screening for insulinoma

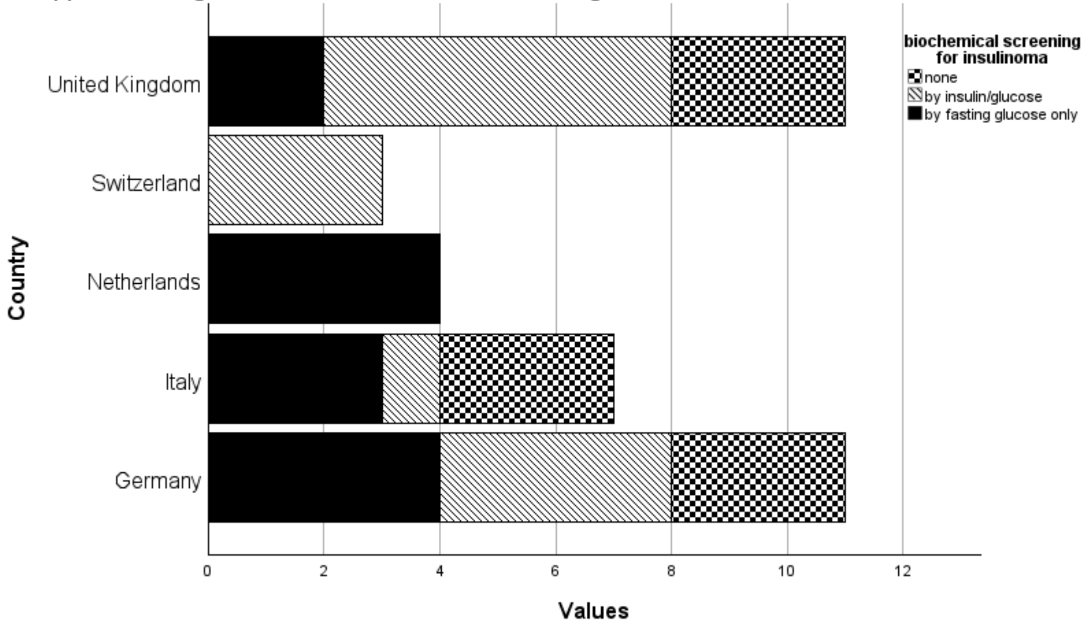

## Supplemental Figure 1F - Adult - CgA included in biochemical screening of asymptomatic adults with MEN1

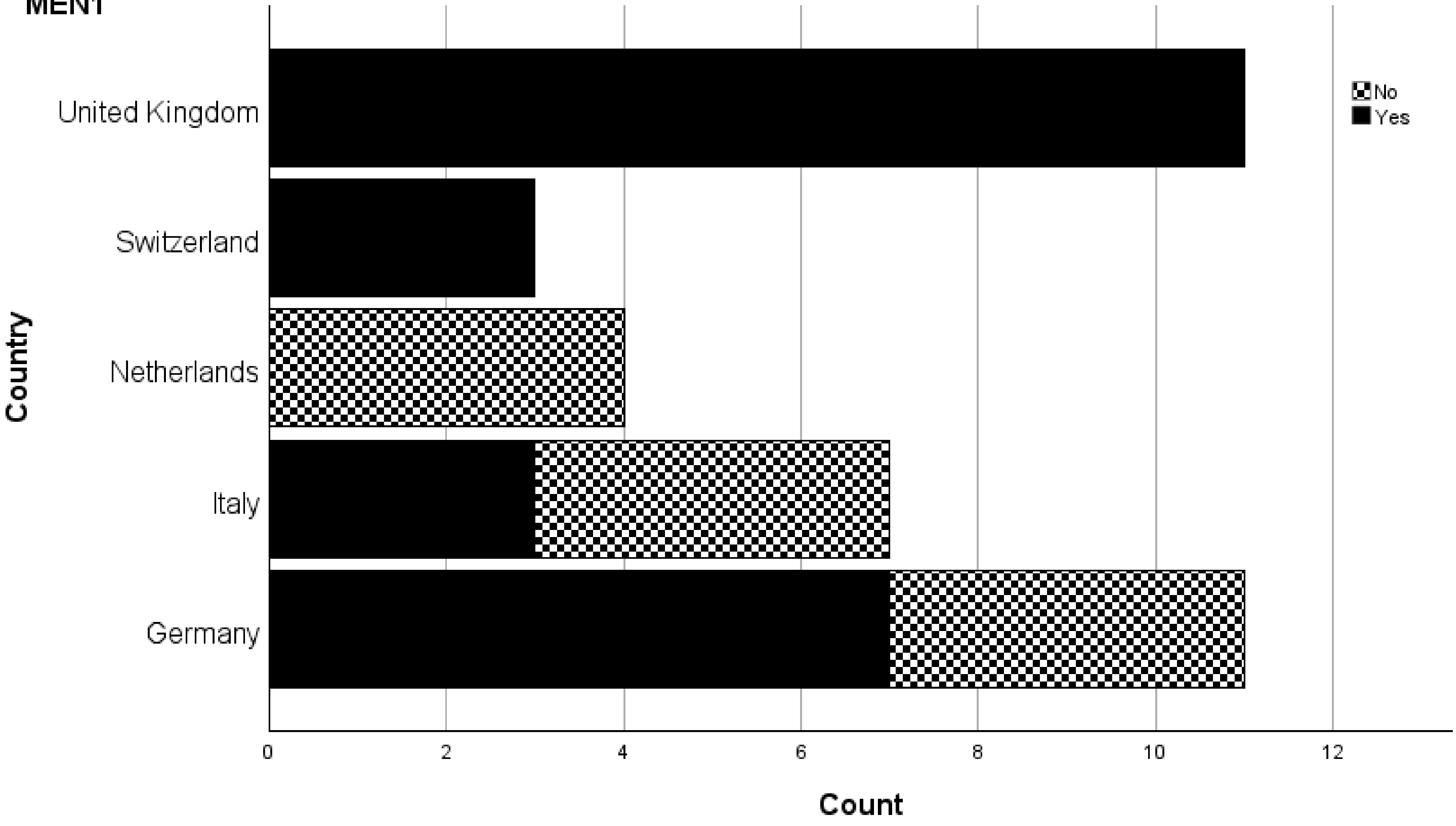

Supplemental Figure 1G - Adult - Fasting Glucagon included in biochemical screening of asymptomatic adults with MEN1

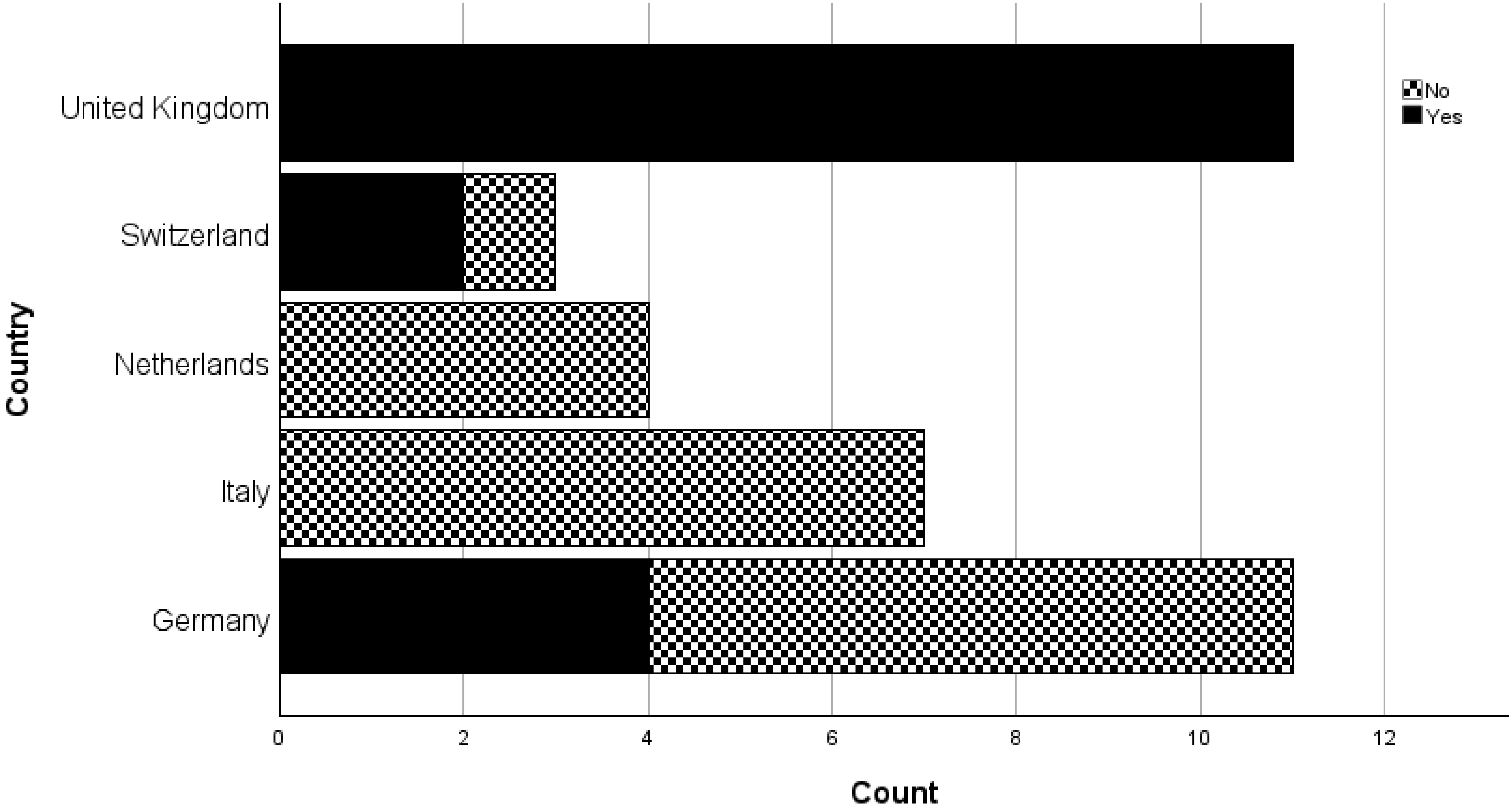

Supplemental Figure 1H - Adult - Fasting Pancreatic Polypeptide included in biochemical screening of asymptomatic adults with MEN1

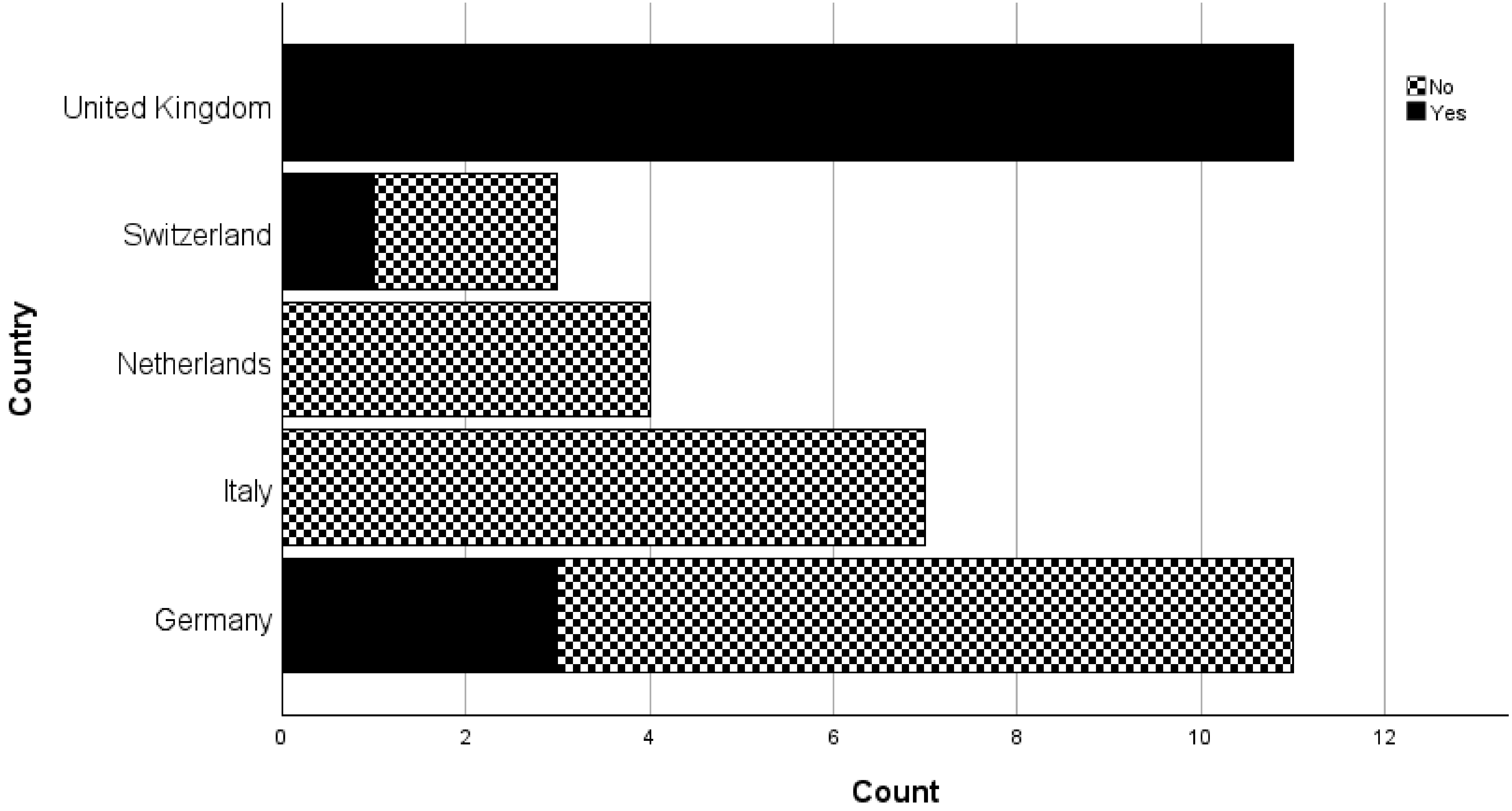

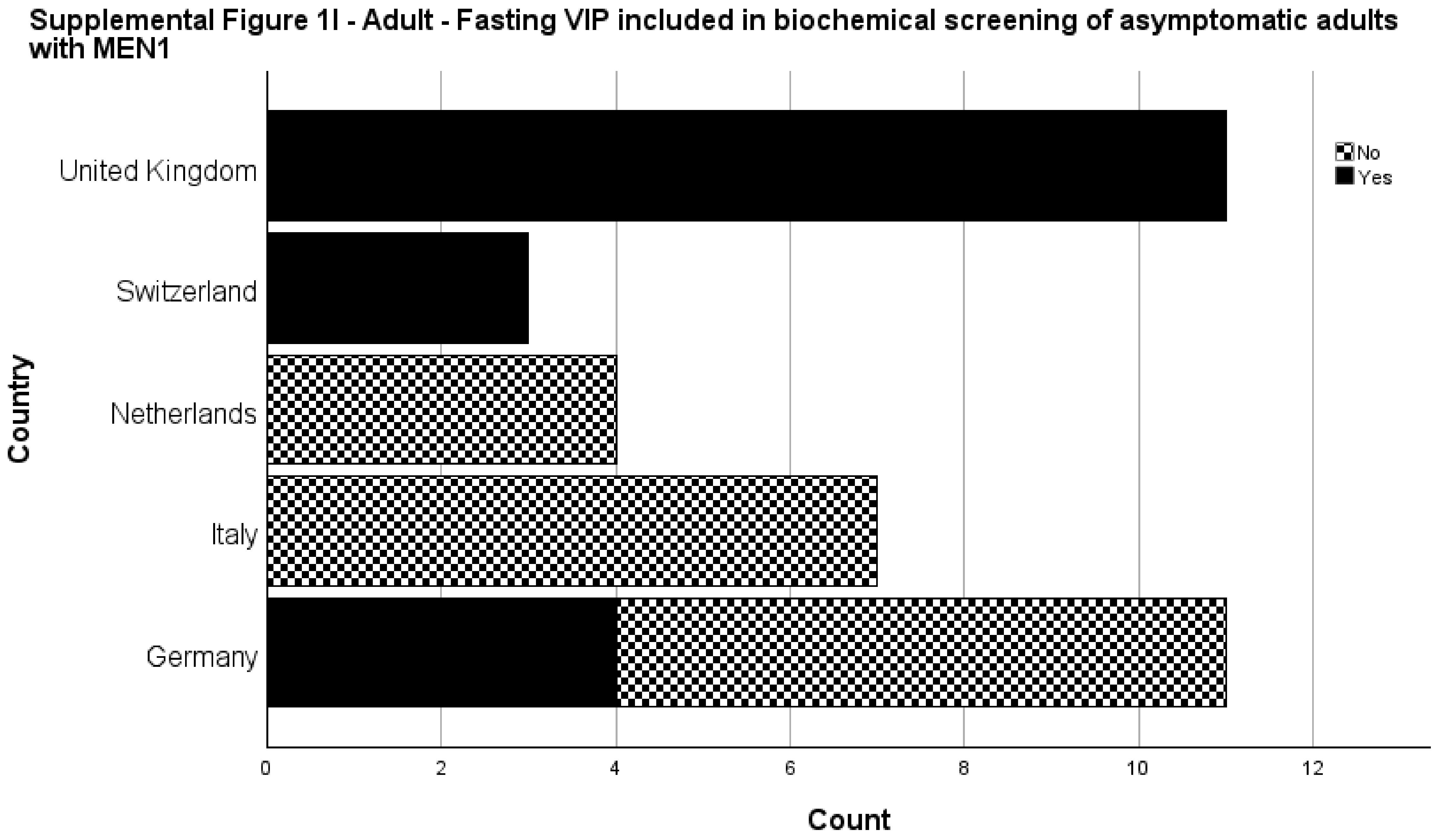
Supplemental Figure 1I - Adult - Fasting VIP included in biochemical screening of asymptomatic adults with MEN1
No
Yes
United Kingdom
Switzerland
Netherlands
Italy
Germany
Country
0
2
4
6
8
10
12
Count

## Supplemental Figure 1J - Adult - Prolactin included in biochemical screening of asymptomatic adults in MEN1

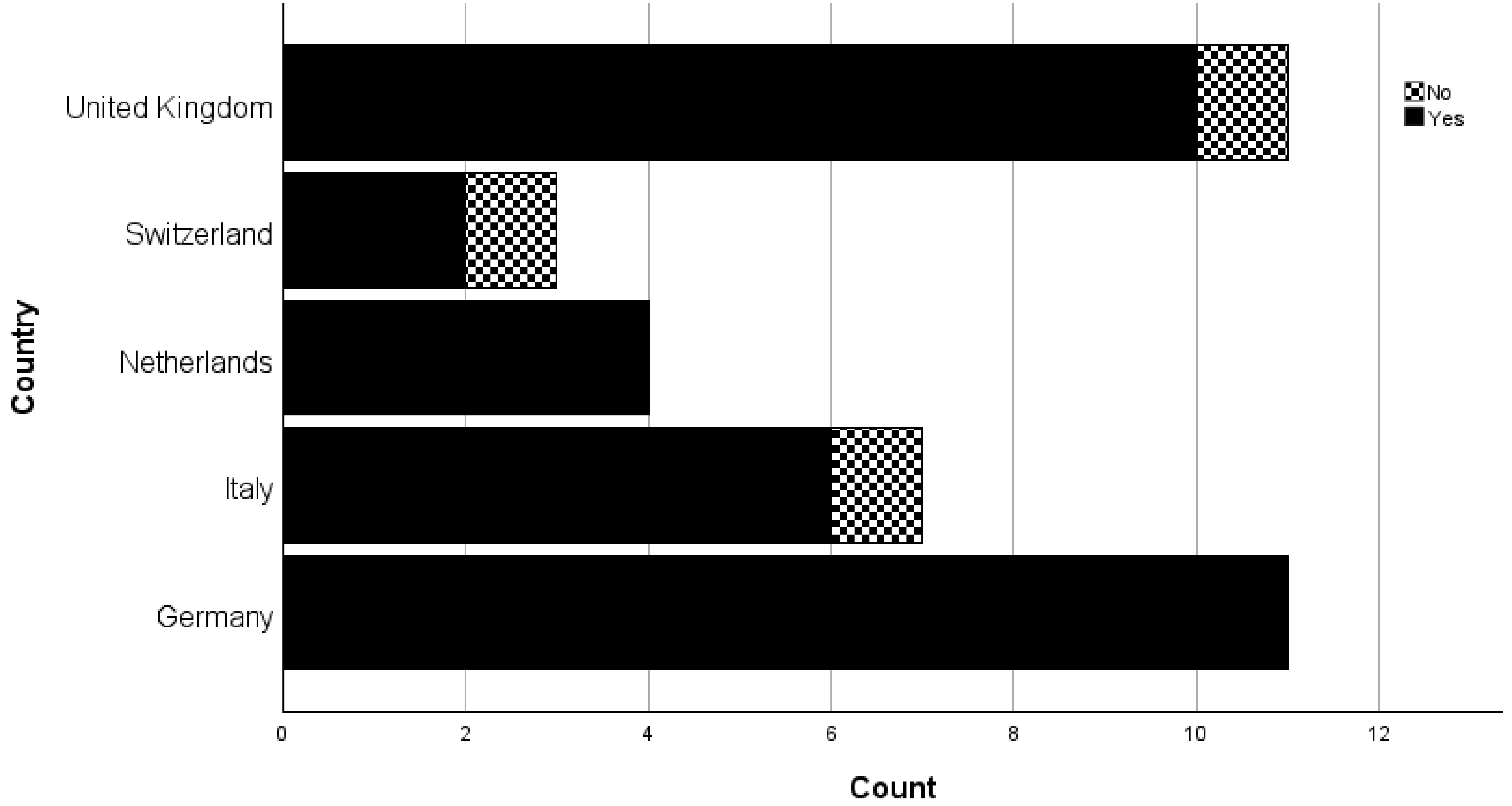

## Supplemental Figure 1K - Adult - IGF1 included in biochemical screening of asymptomatic adults with MEN1

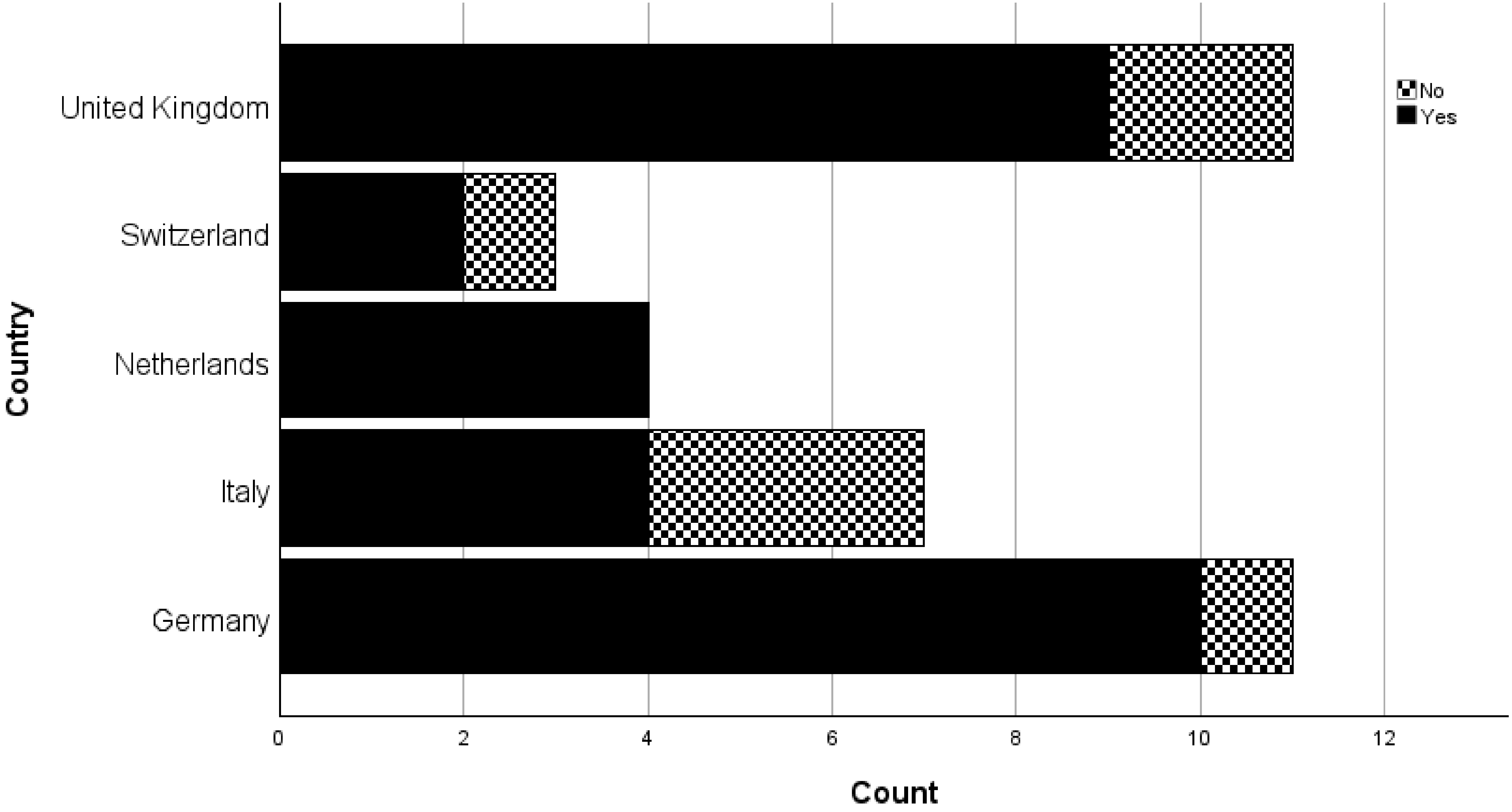

## Supplemental Figure 1L - Adult - Sex hormones measured in biochemical screening of adults with MEN1

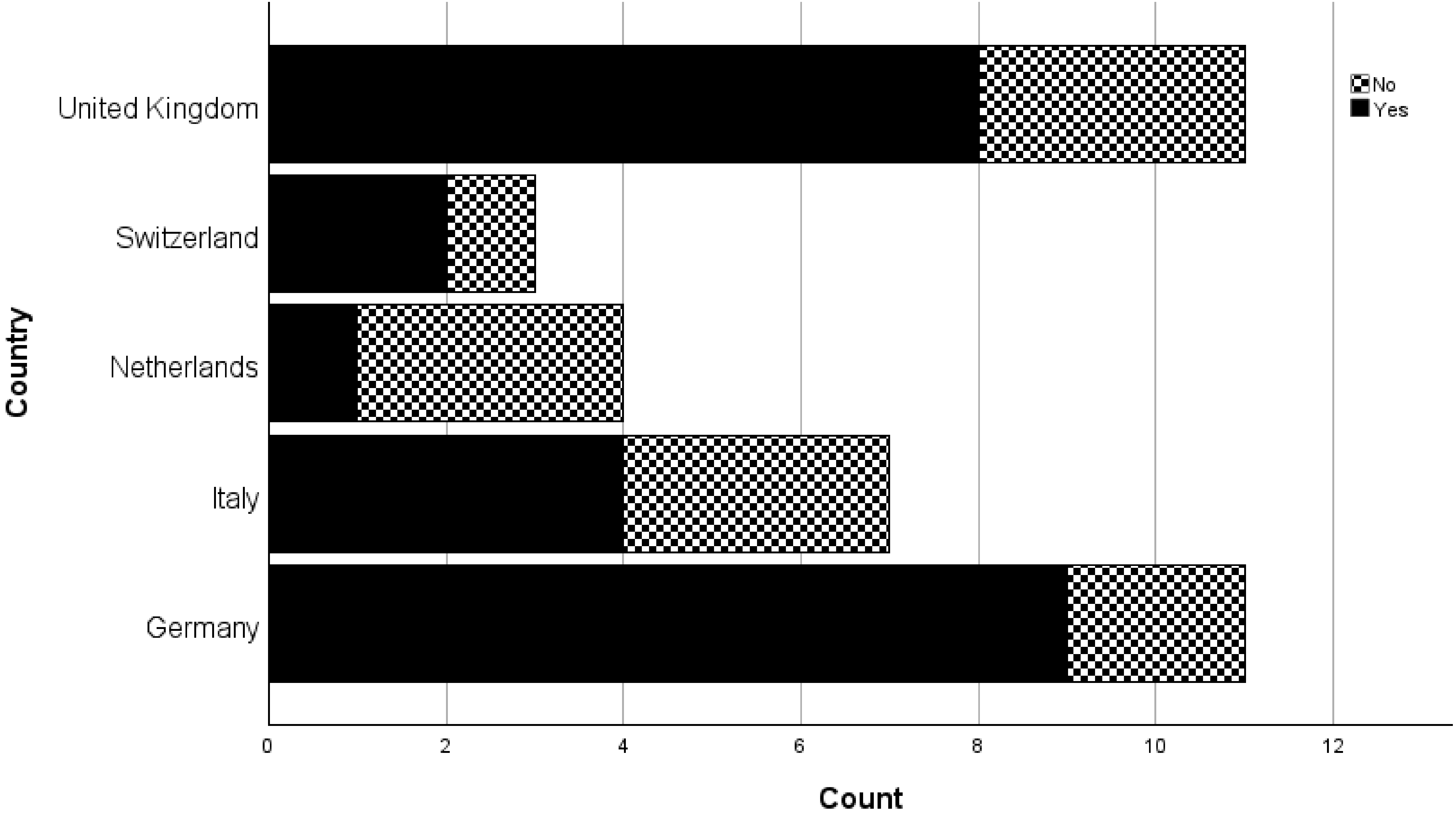

**Suppelmental Figure 1M - Adult - Thyroid hormones included in biochemical screening of adults with MEN1**

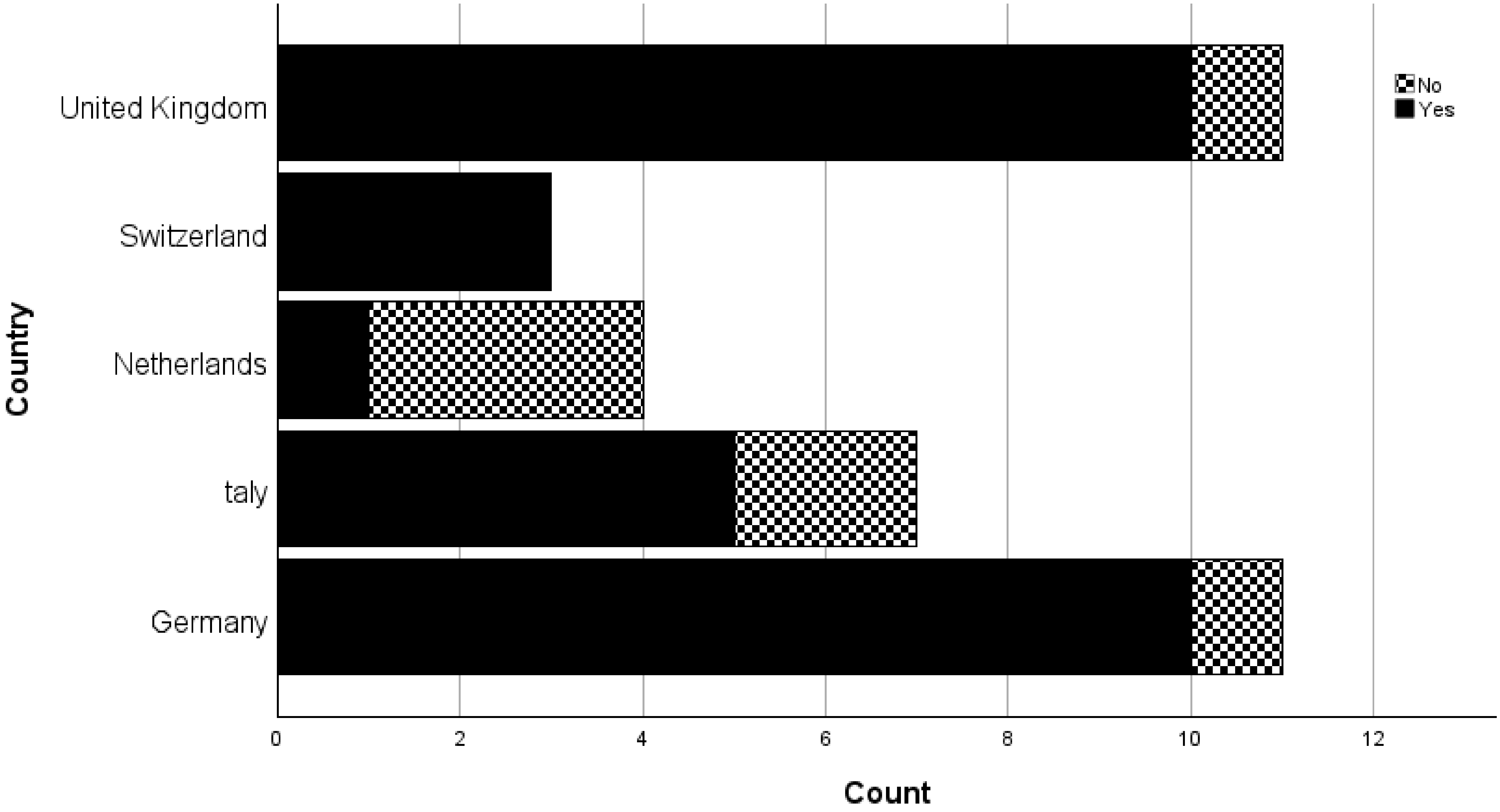

Supplemental Figure 1N - Adult - Cortisol included in biochemical screening of asymptomatic adults with MEN1

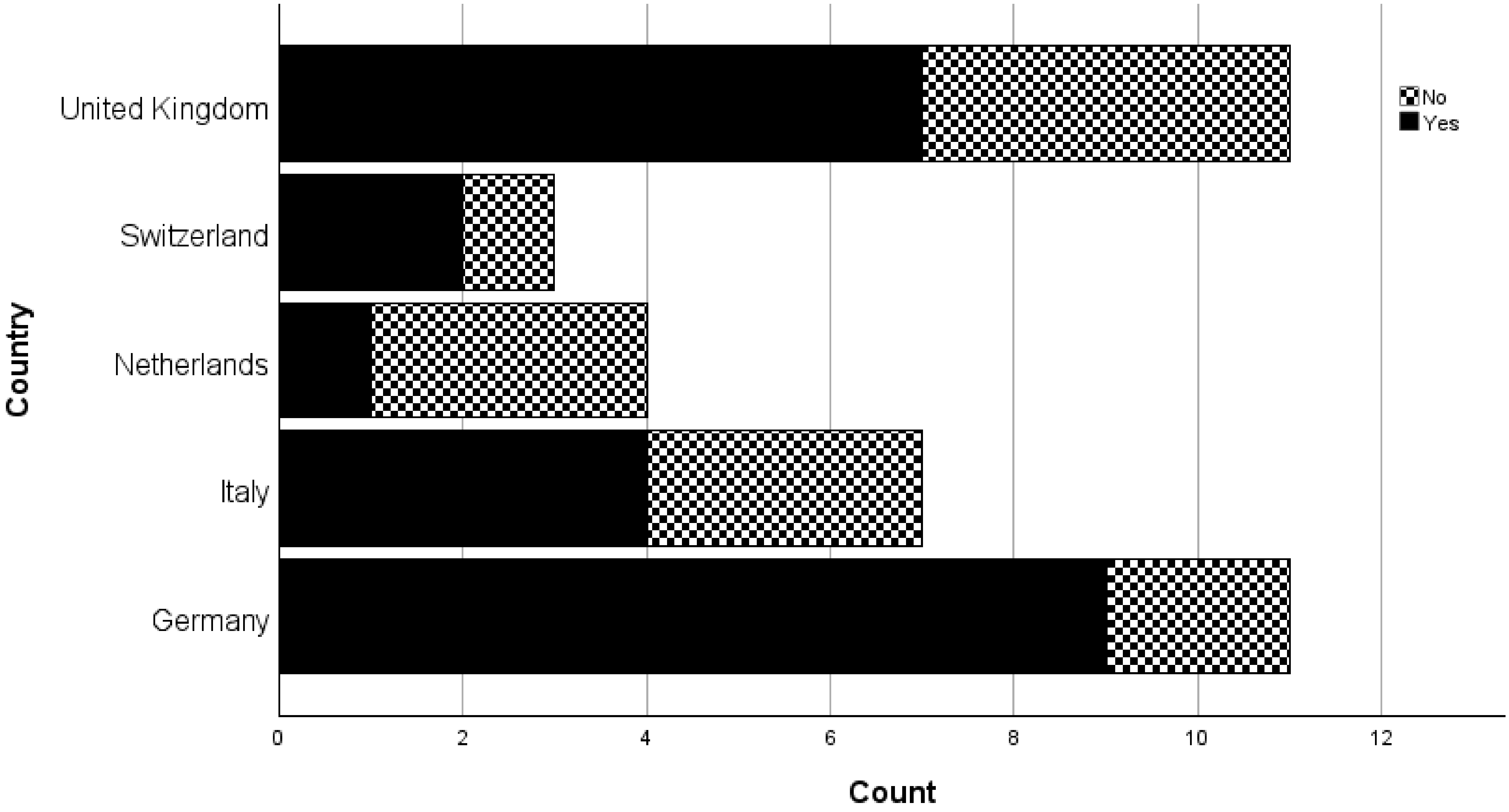

# Supplemental Figure 2

Biochemical gastrinoma diagnosis and EGD in adults with MEN1 by country – for countries from which 3 or more CoEs provided answers

Supplemental Figure 2A - Biochemical modalities used for gastrinoma diagnosis - FSG alone

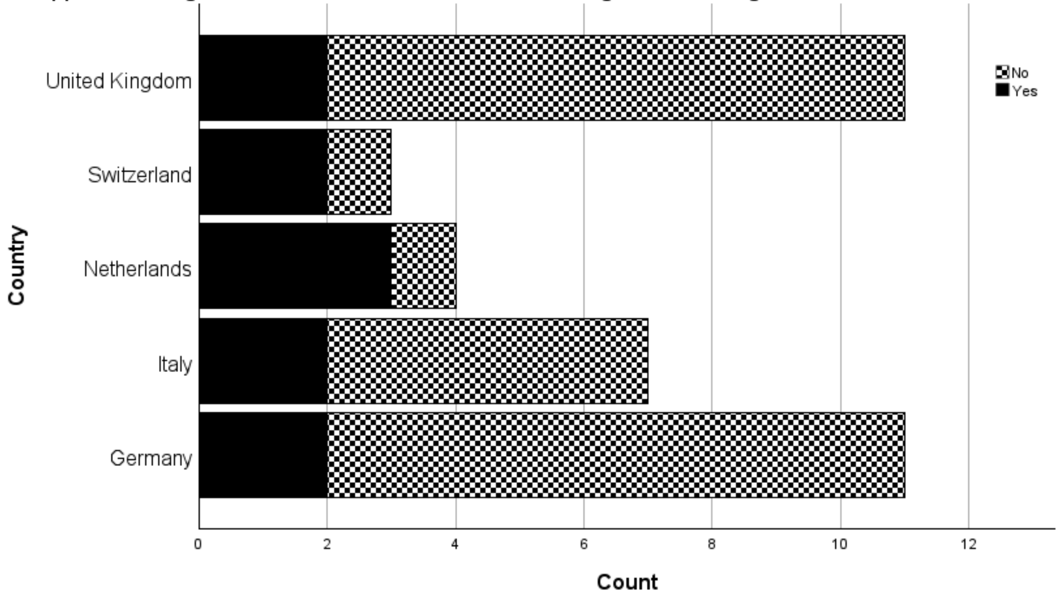


Supplemental Figure 2B - Biochemical modalities used for gastrinoma diagnosis include both FSG and gastric pH

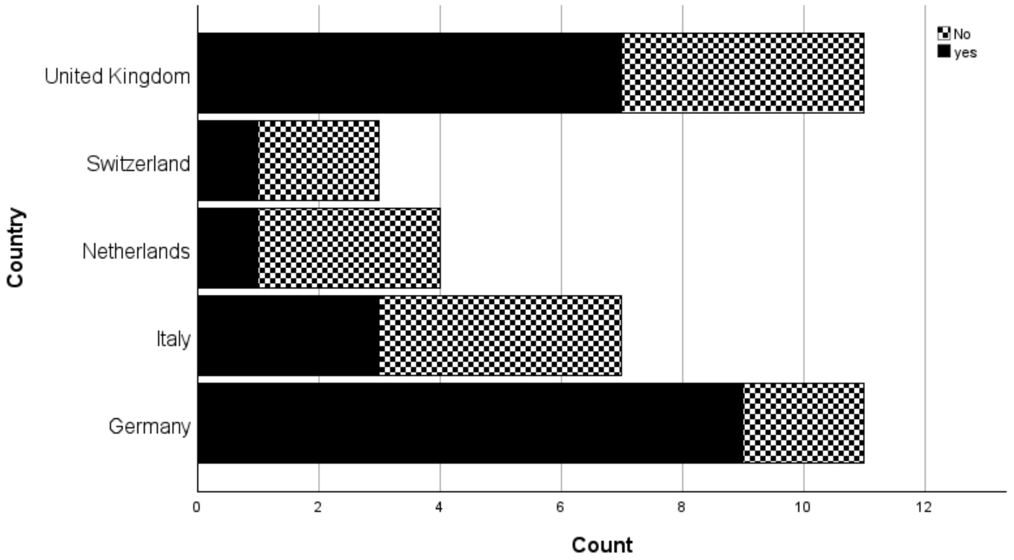

**Supplemental Figure 2C - Biochemical modalities generally used for the diagnosis in patients with MEN1 - Fasting Serum Gastrin**

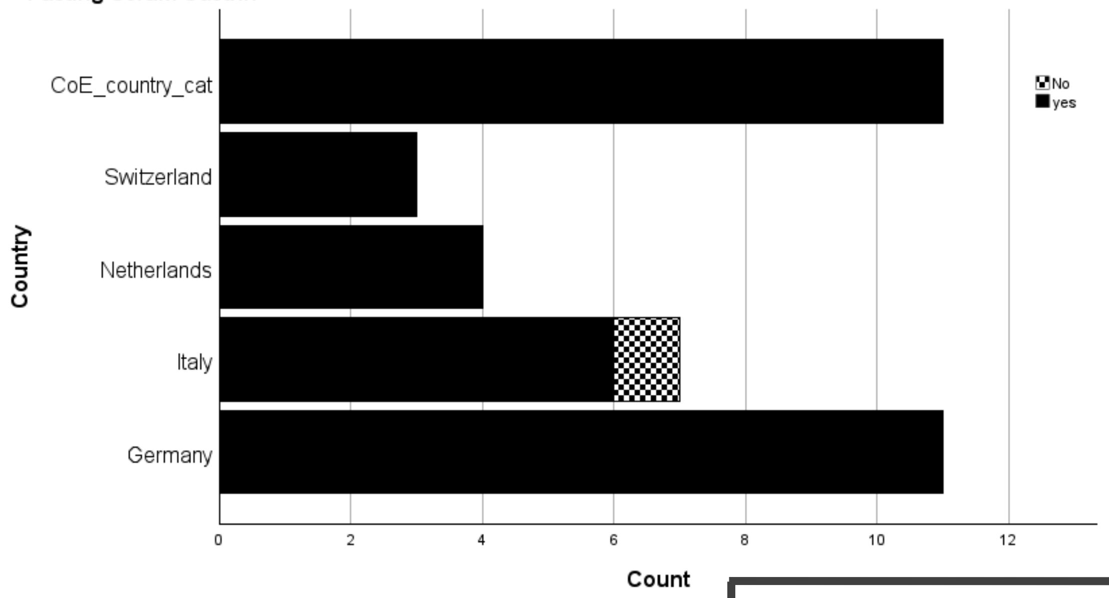


**Supplemental Figure 2D - Biochemical modalities generally used for gastrinoma diagnosis in patients with MEN1 - Gastric pH**

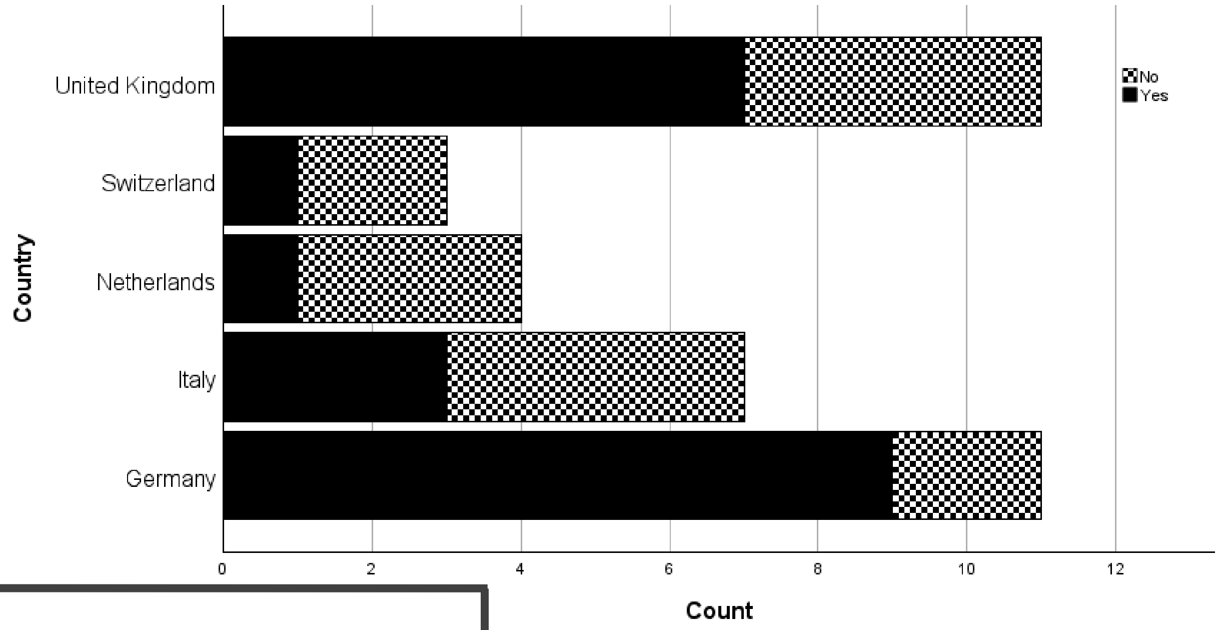


**Supplemental Figure 2F - Biochemical modalitites used for gastrinoma diagnosis in patients with MEN1 - Basal Acid Output**

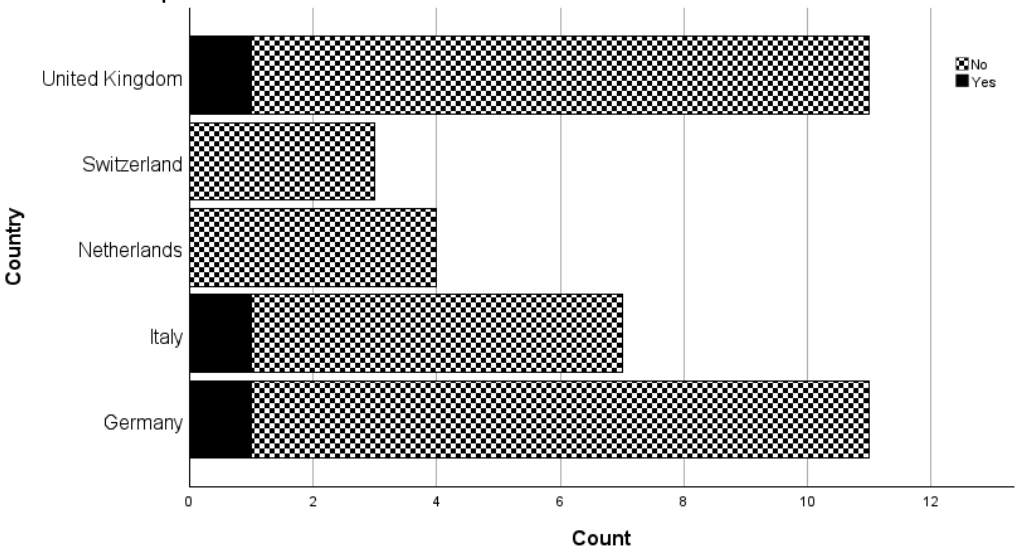


**Supplemental Figure 2E - Biochemical modalities generally used for gastrinoma diagnosis in patients with MEN1 - Secretin Test**

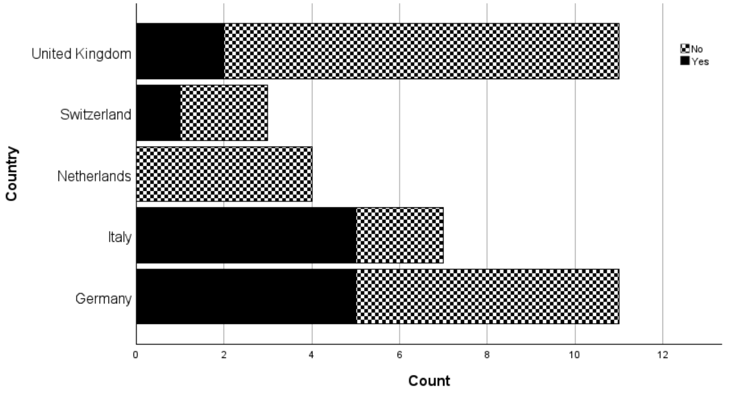


**Supplemental Figure 2G - Biochemical modalities generally used for gastrinoma diagnosis in patients with MEN1 - Calcium test**

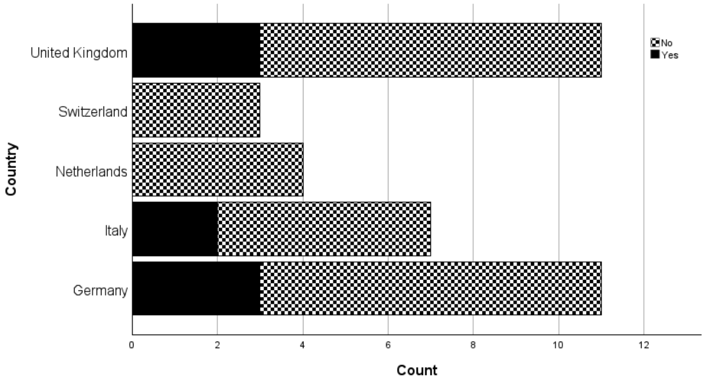

## Supplemental Figure 2H - Secretin test used - all entries by country

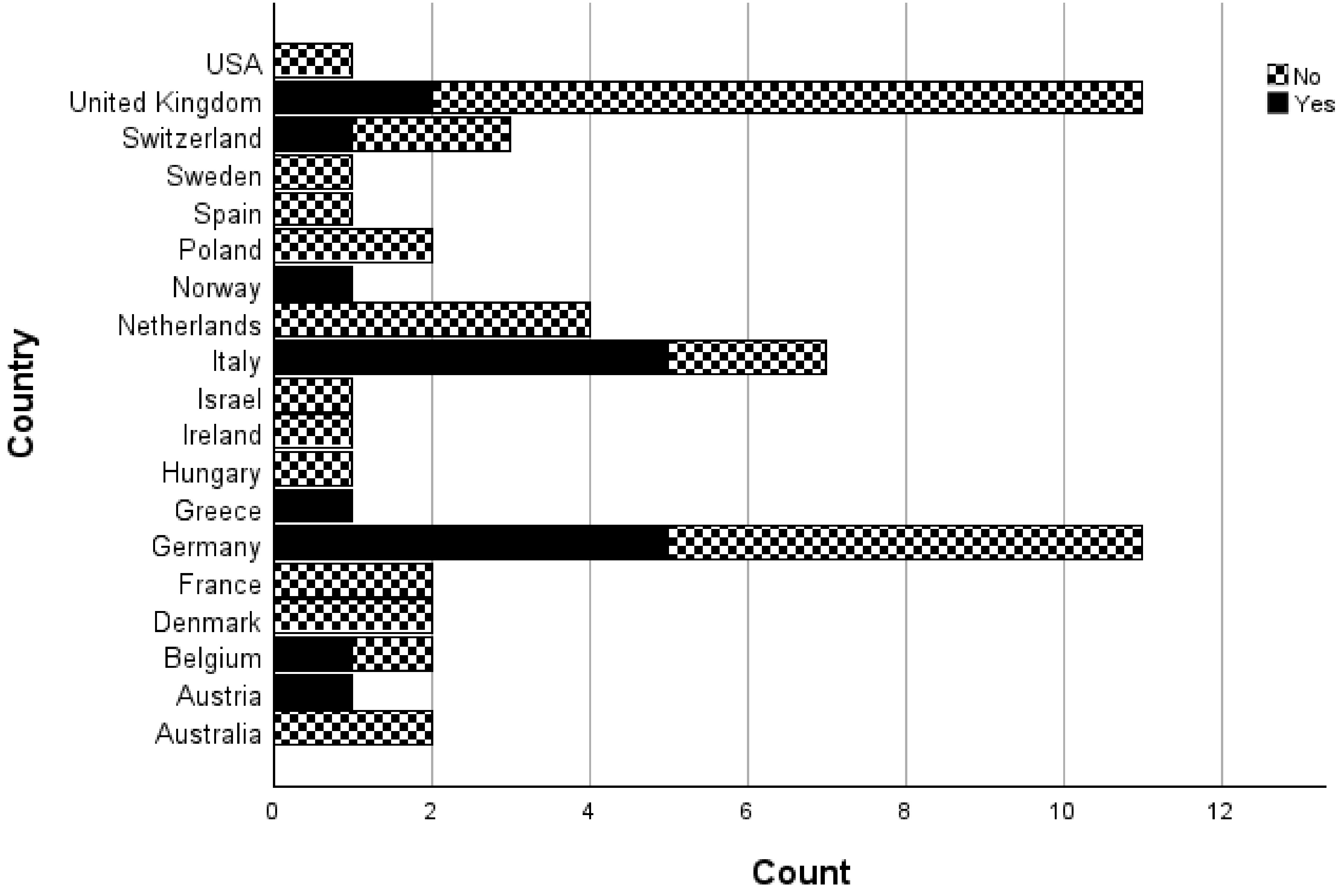

## Supplemental Figure 2I - Is EGD routinely performed in patients with MEN1?

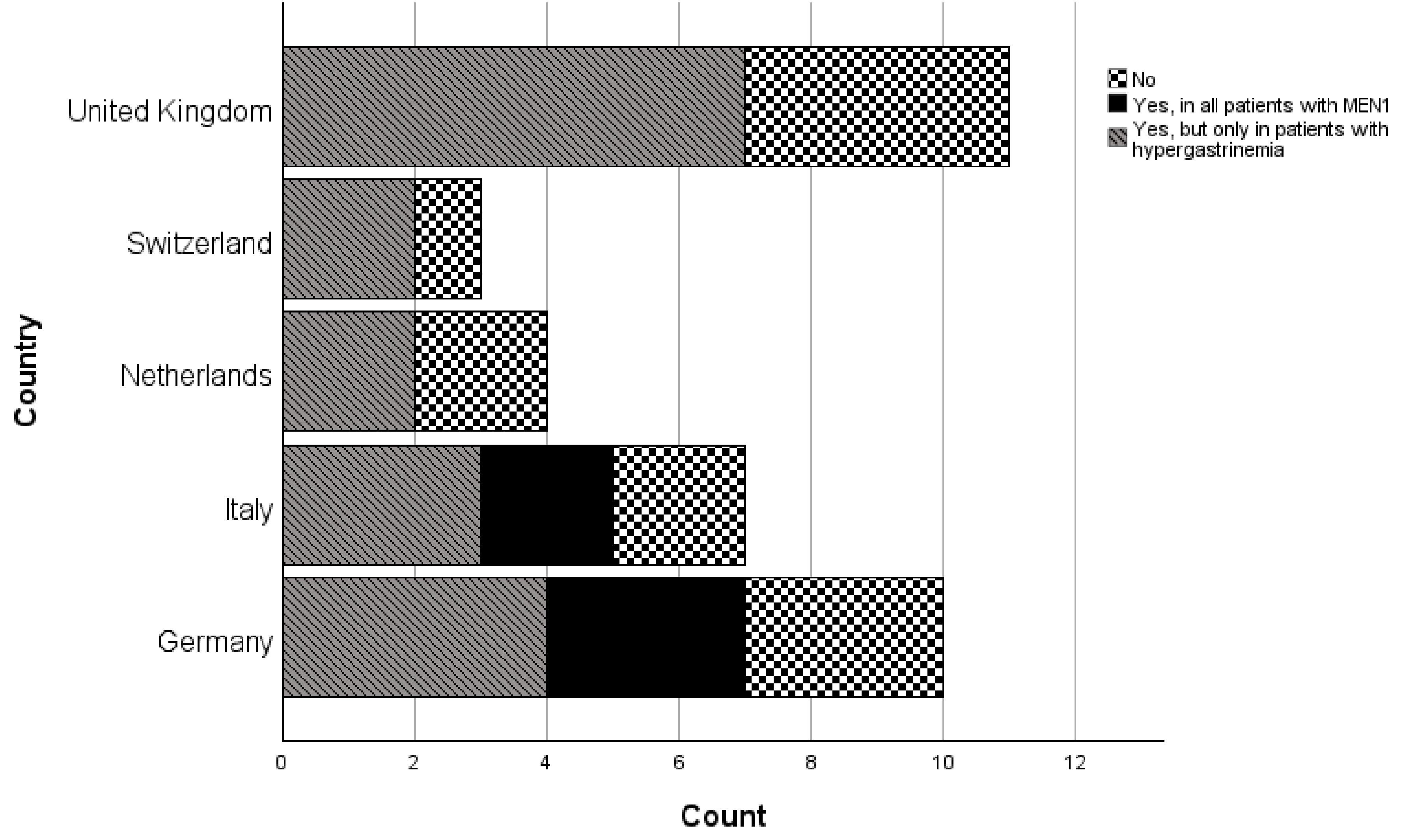

# Supplemental Figure 3

EUS in adults with MEN1 by country – for countries from which 3 or more CoEs provided answers

Supplemental Figure 3A - EUS used routinely for screening in patients without PanNETs

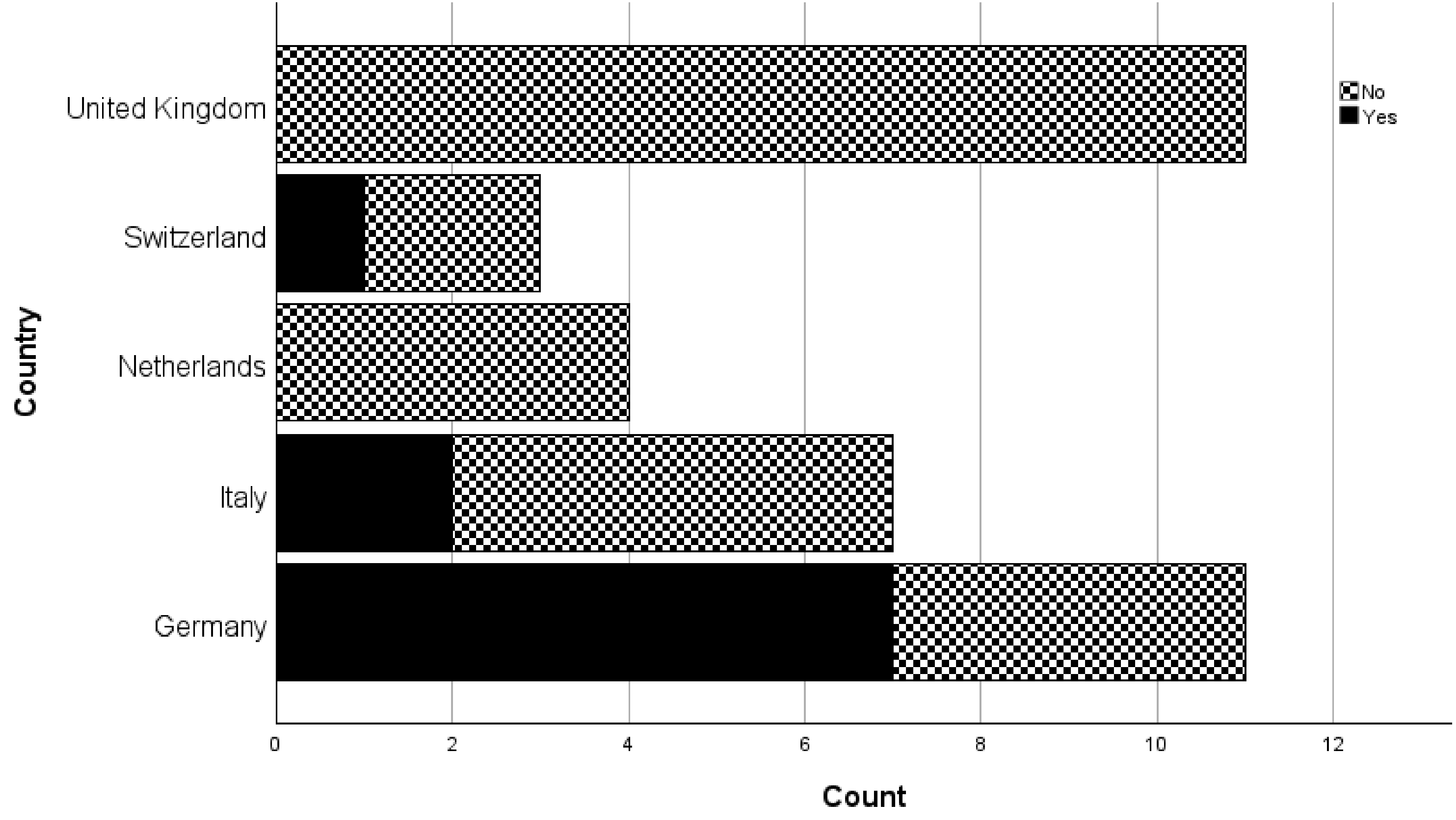

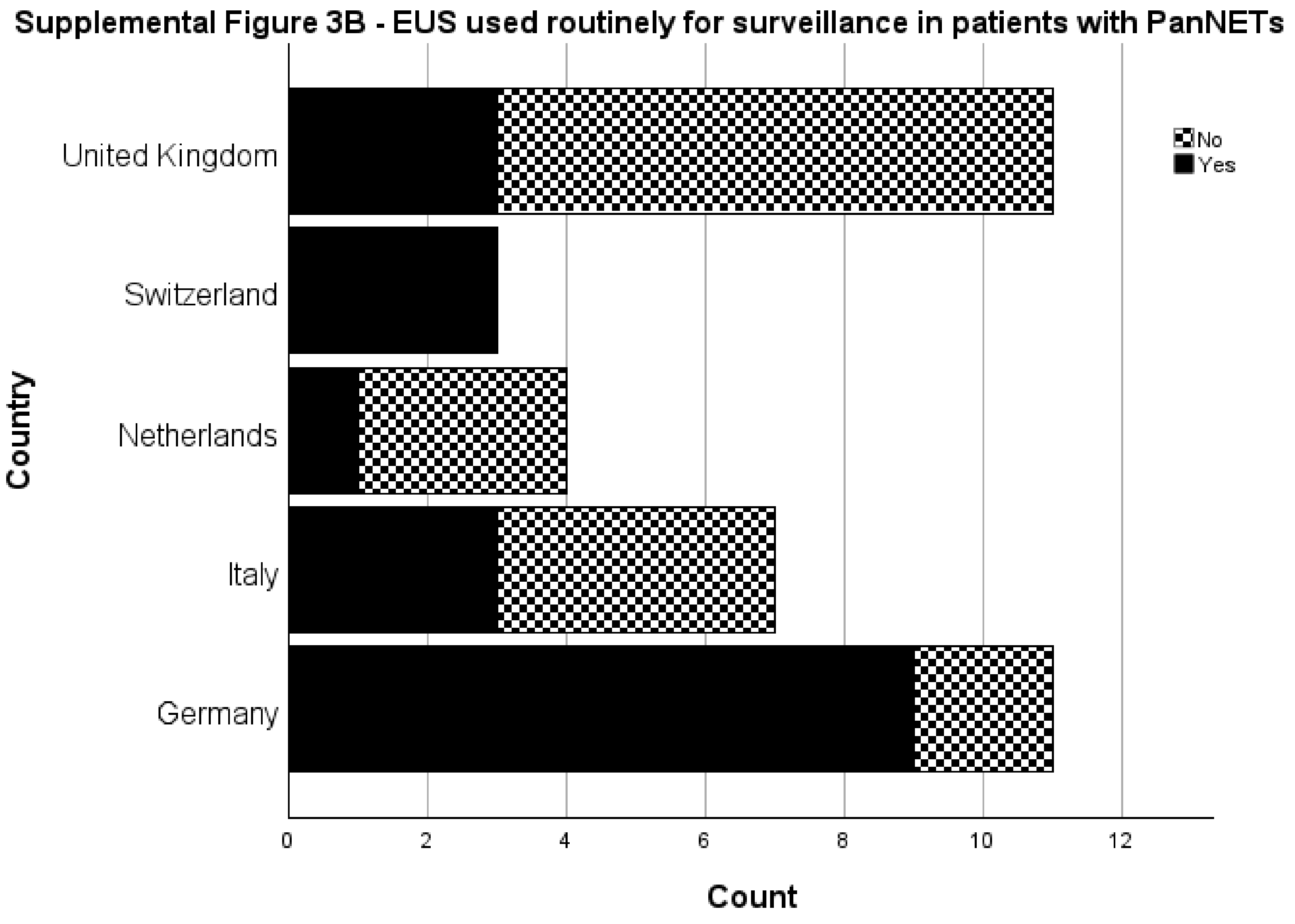
Supplemental Figure 3B - EUS used routinely for surveillance in patients with PanNETs
No
Yes
United Kingdom
Switzerland
Netherlands
Italy
Germany
Country
0
2
4
6
8
10
12
Count

Supplemental Figure 3C - EUS used on specific indication

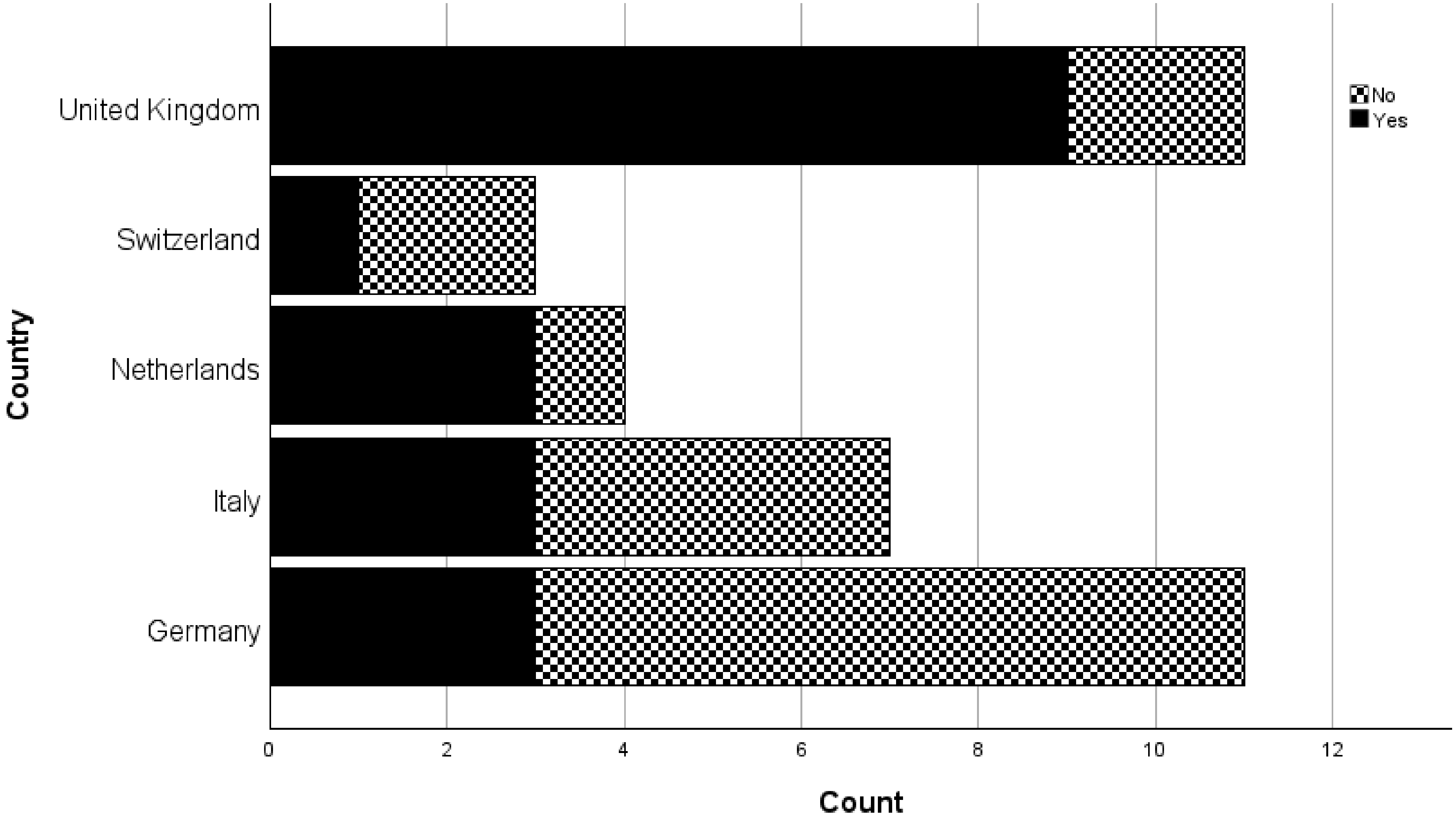

Supplemental Figure 3D - Use of EUS-FNA/FNB

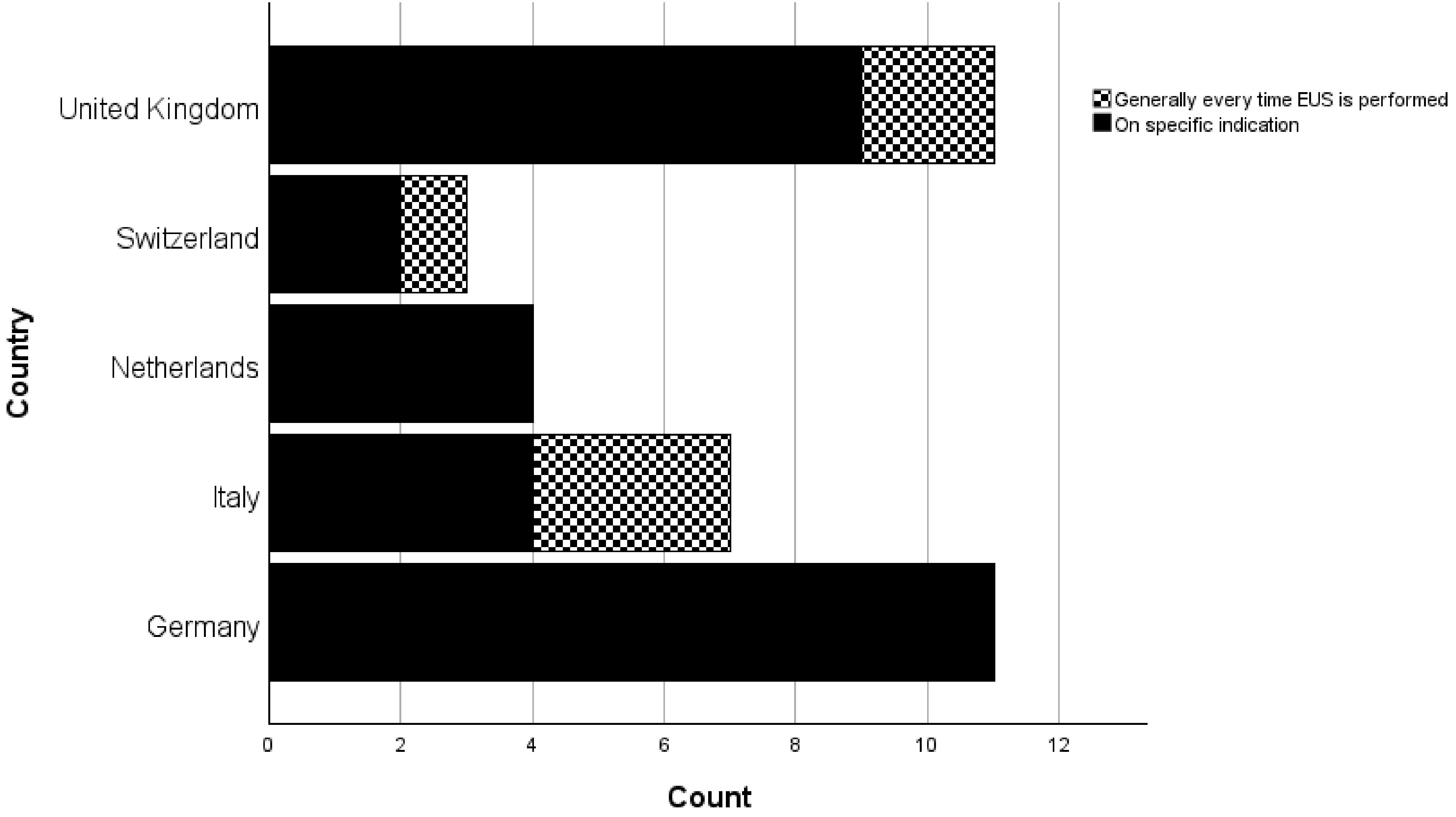

# Supplemental Figure 4

Gallium-DOTA-peptides-PET/CT in adults with MEN1 by country – for countries from which 3 or more CoEs provided answers

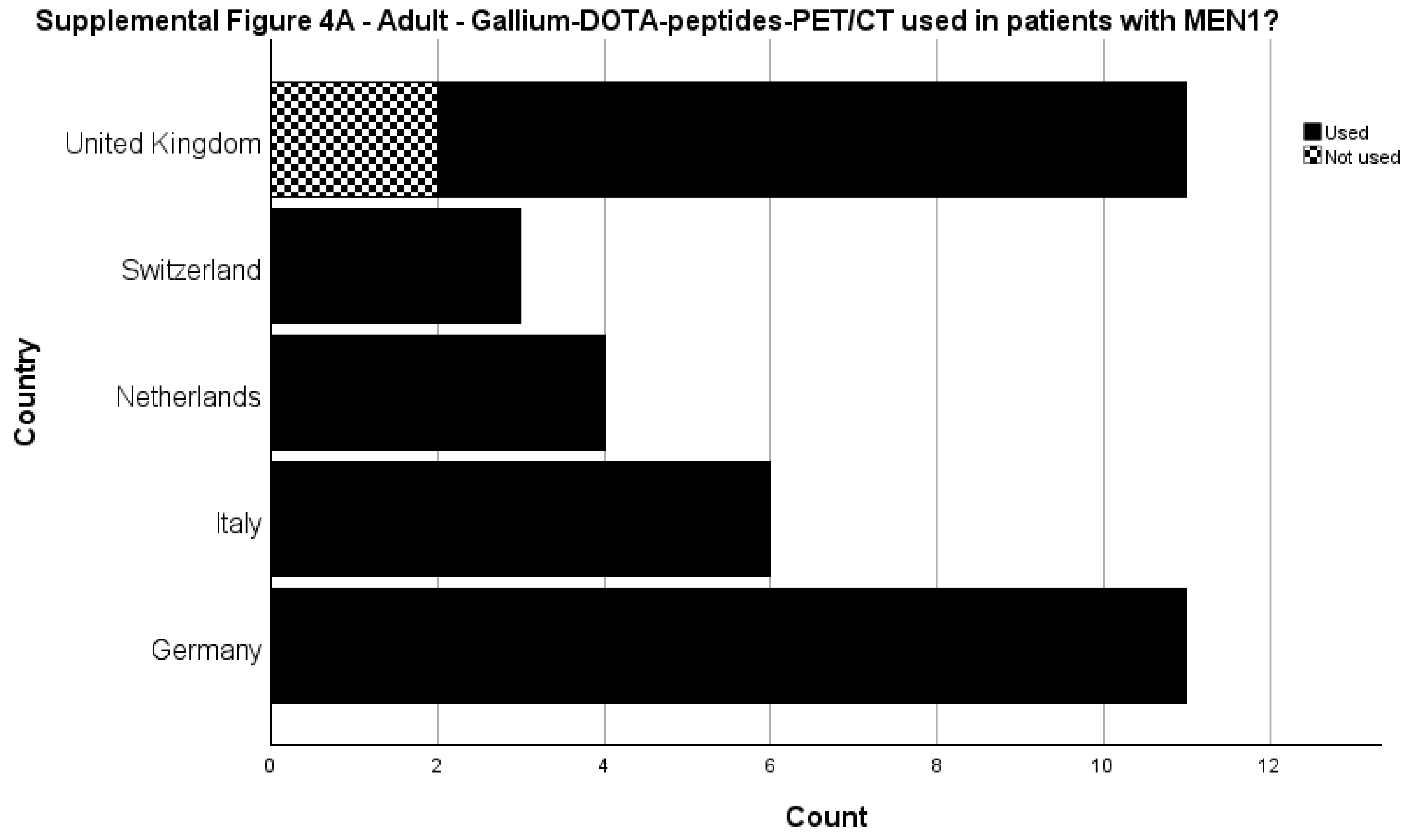
Supplemental Figure 4A - Adult - Gallium-DOTA-peptides-PET/CT used in patients with MEN1?
Country
United Kingdom
Switzerland
Netherlands
Italy
Germany
0
2
4
6
8
10
12
Count
Used
Not used

**Supplemental Figure 4B - Adult - Gallium-DOTA-peptides-PET/CT - regular use in surveillance of metastatic NETs**

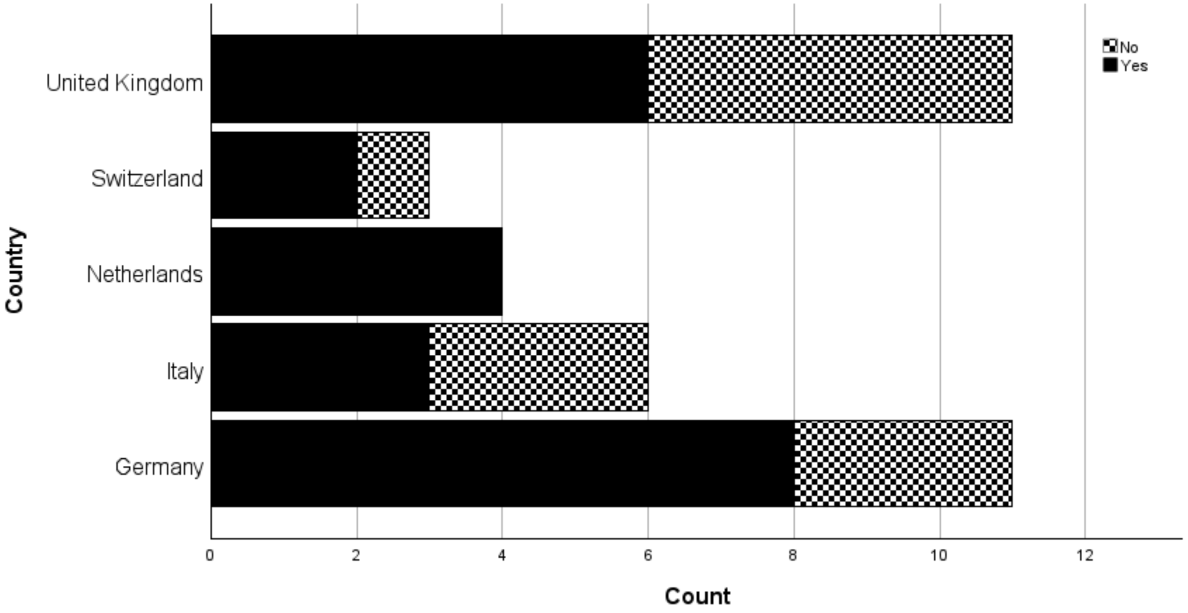


**Supplemental Figure 4C - Adult - Gallium-DOTA-peptides-PET/CT - regular use in the surveillance of non-metastatic NETs**

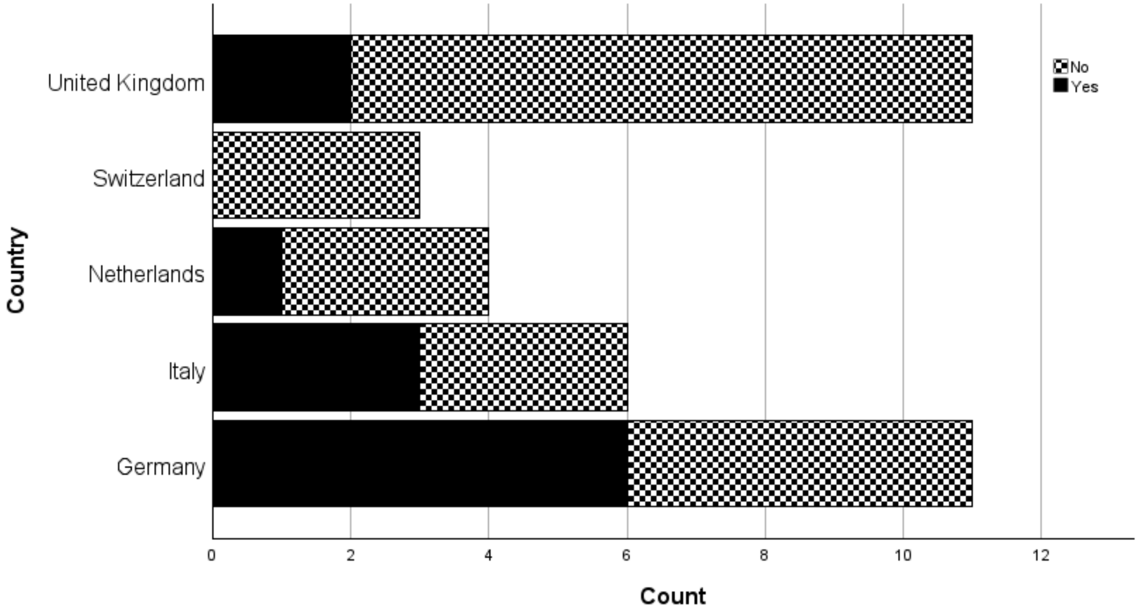


**Supplemental Figure 4D - Adult - Gallium-DOTA-peptides-PET/CT - regular use in screening of patients without known NETs**

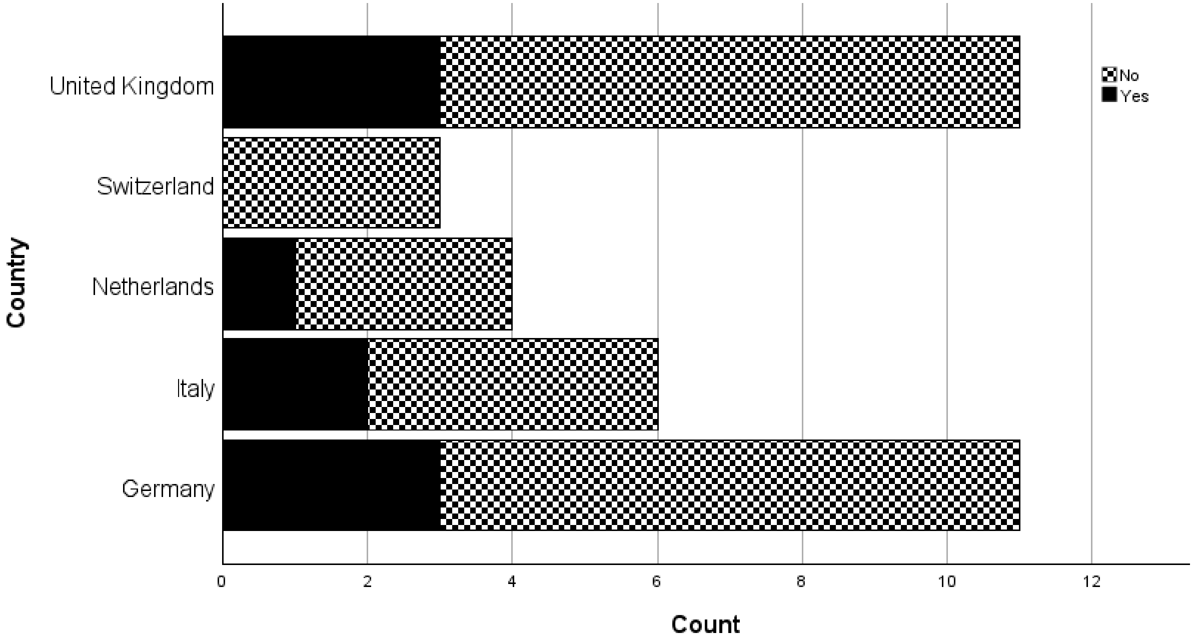


**Supplemental Figure 4E - Adult - Gallium-DOTA-peptides-PET/CT used for specific indication**

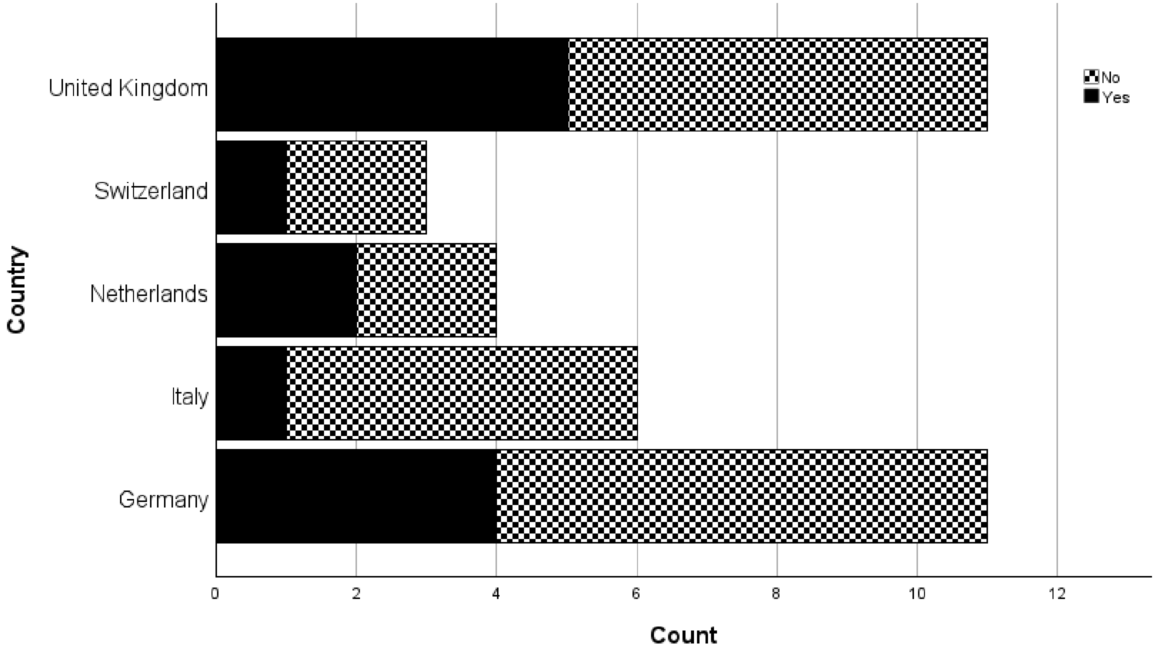

# Supplemental Figure 5

Surveillance of small PanNETs in adults with MEN1 by country – for countries from which 3 or more CoEs provided answers

**Supplemental Figure 5A - Adult - MRI (one of the) preferred imaging modality(ies) for surveillance of PanNETs**

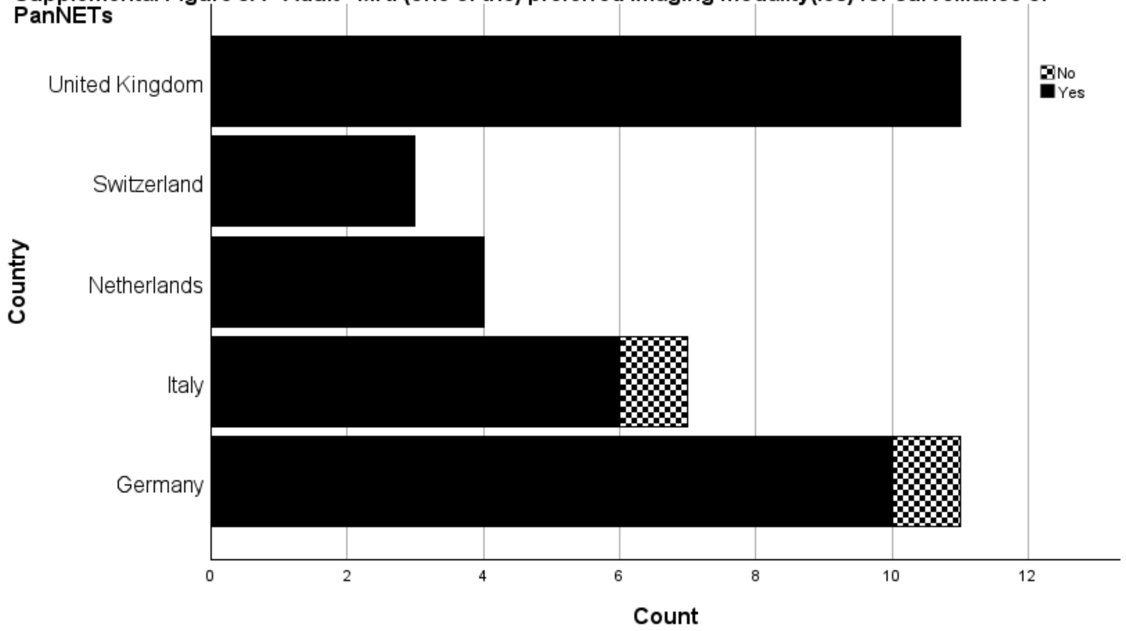


**Supplemental Figure 5B - Adult - CT (one of the) preferred imaging modality(ies) for surveillance of PanNETs**

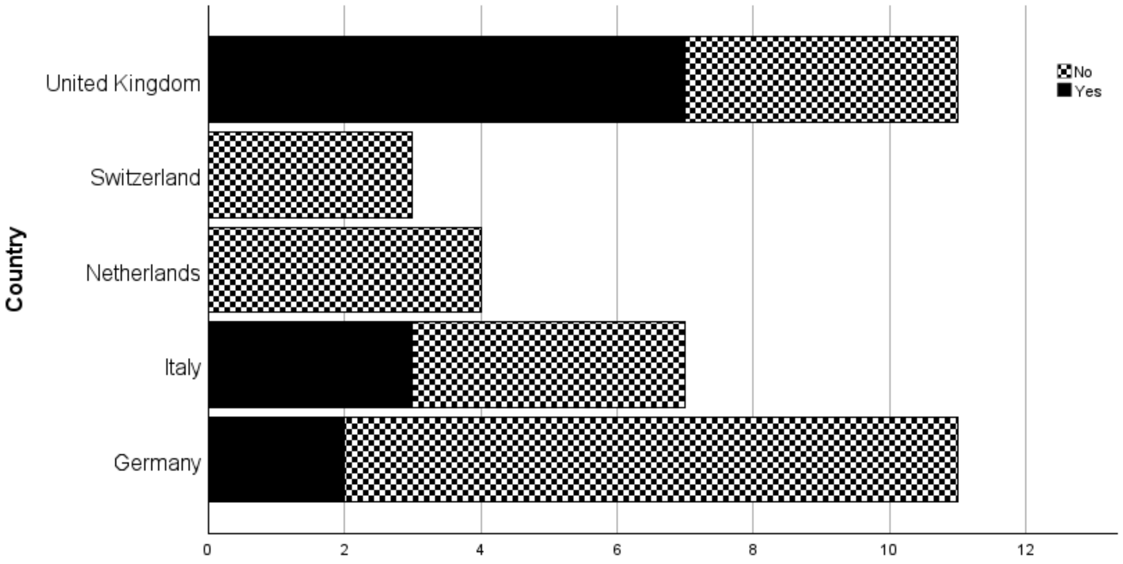


**Supplemental Figure 5C - Adult - EUS (on of the) preferred imaging modality(ies) for surveillance of PanNETs**

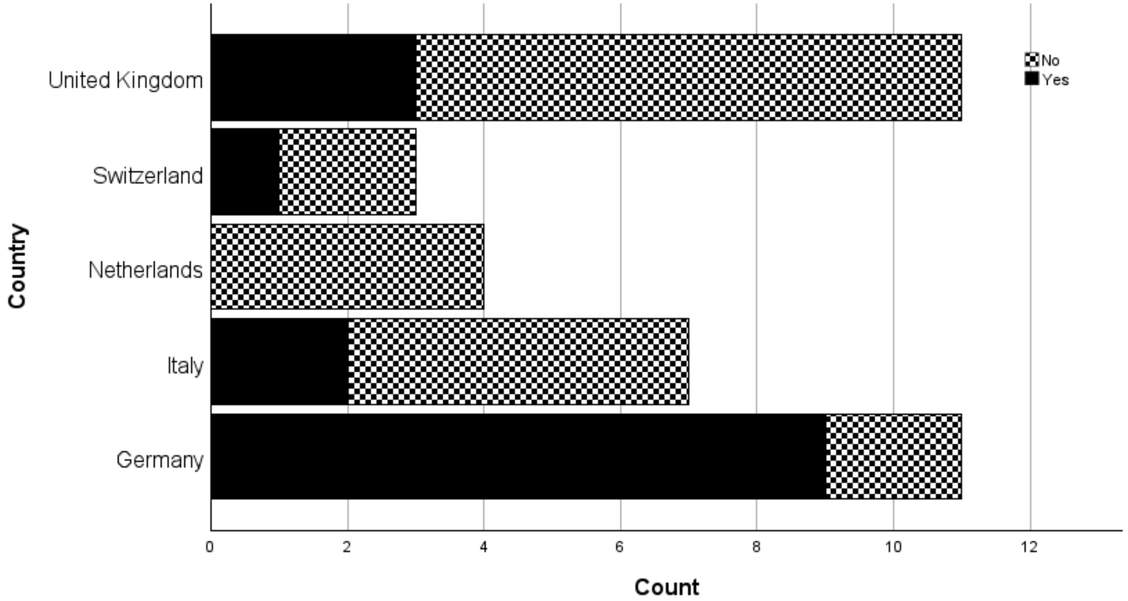


**Supplemental Figure 5D - Adult - Gallium-DOTA-peptides-PET/CT (one of the) preferred imaging modality (ies) for surveillance of PanNETs**

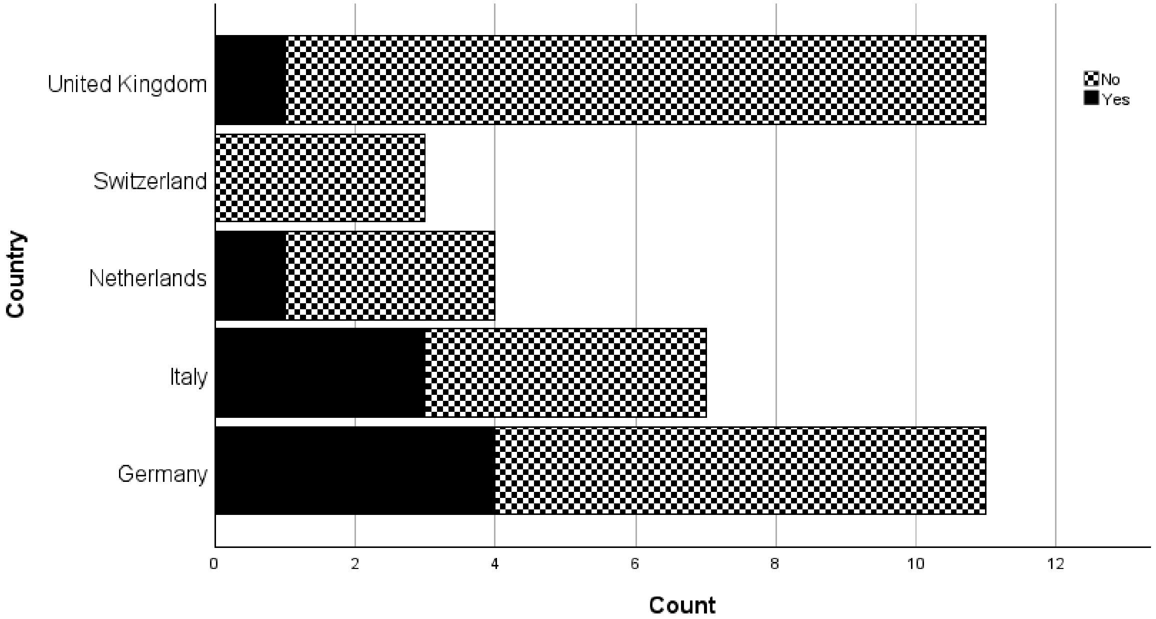

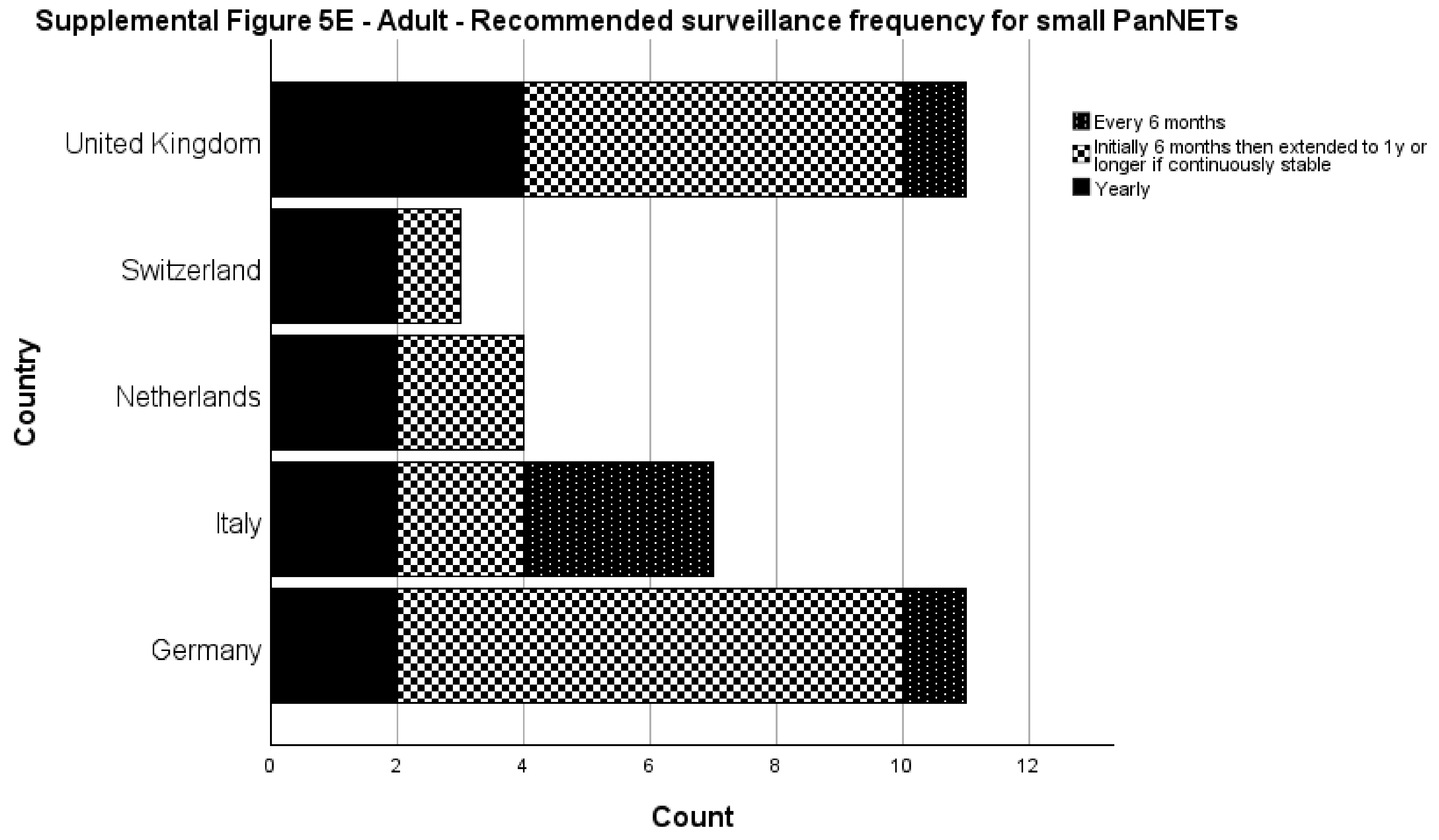

Supplemental Figure 5E - Adult - Recommended surveillance frequency for small PanNETs
United Kingdom
Switzerland
Netherlands
Italy
Germany
Country
0
2
4
6
8
10
12
Count
Every 6 months
Initially 6 months then extended to 1y or longer if continuously stable
Yearly

# Supplemental Figure 6

Thoracic screening and surveillance in adults with MEN1 by country – for countries from which 3 or more CoEs provided answers

Supplemental Figure 6A - Adult - CT (one of the) preferred imaging modality(ies) for screening/surveillance of thoracic NETs

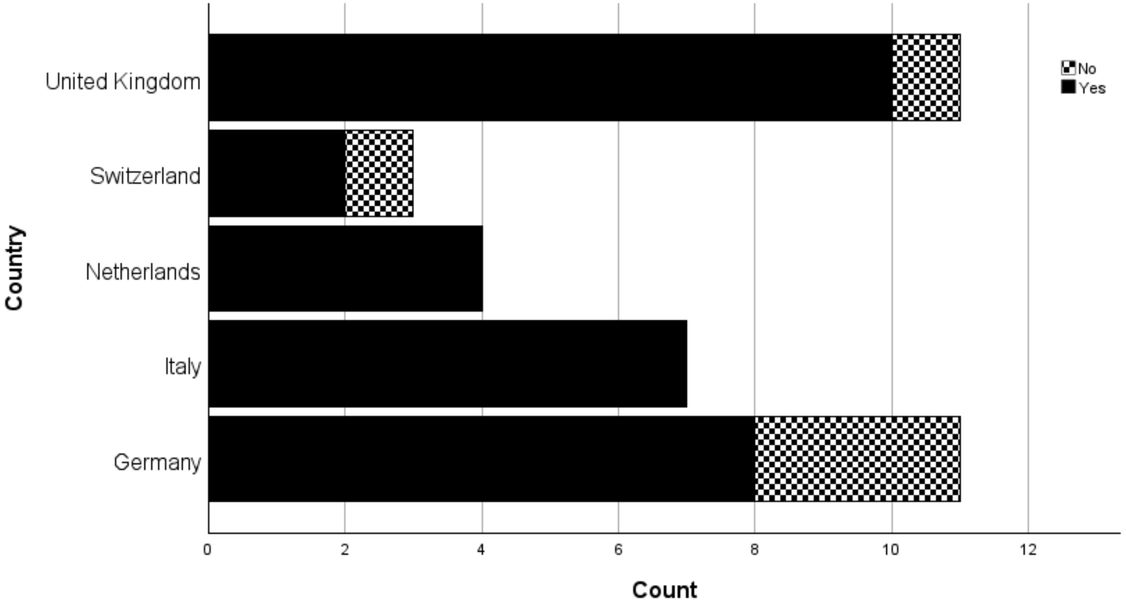


Supplemental Figure 6B - Adult - Gallium-DOTA-peptides-PET/CT (one of the) preferred imaging modality (ies) for screening/surveillance of thoracic NETs

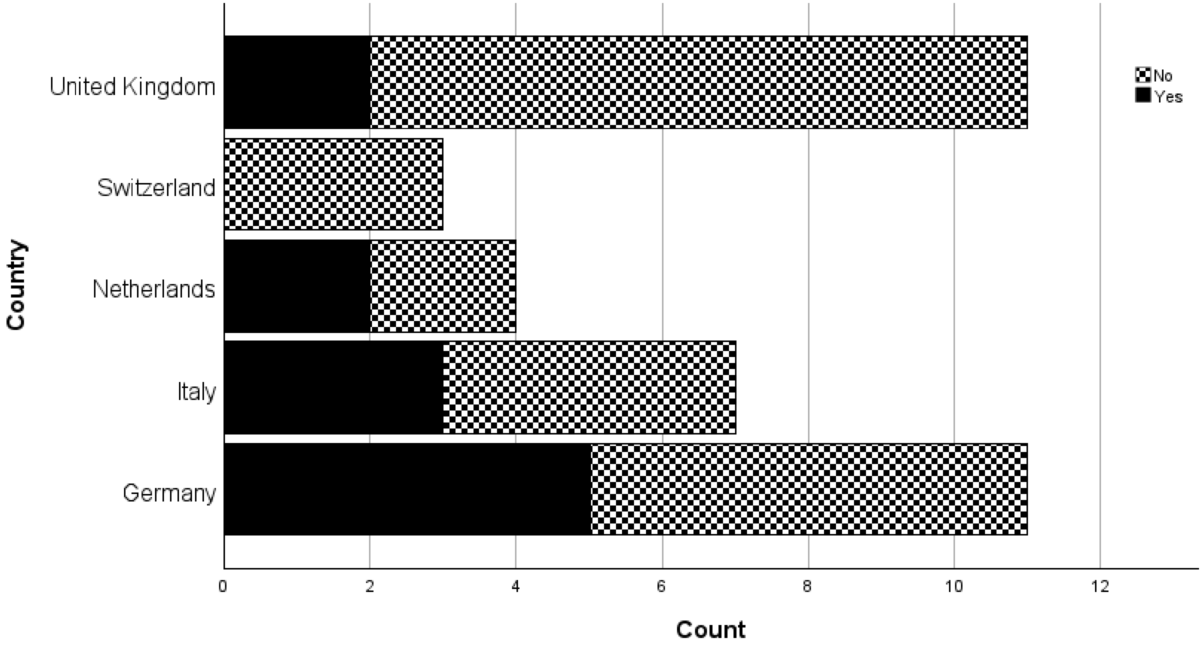


Supplemental Figure 6C - Adult - MRI (one of the) preferred imaging modality(ies) for screening/surveillance of thoracic NETs

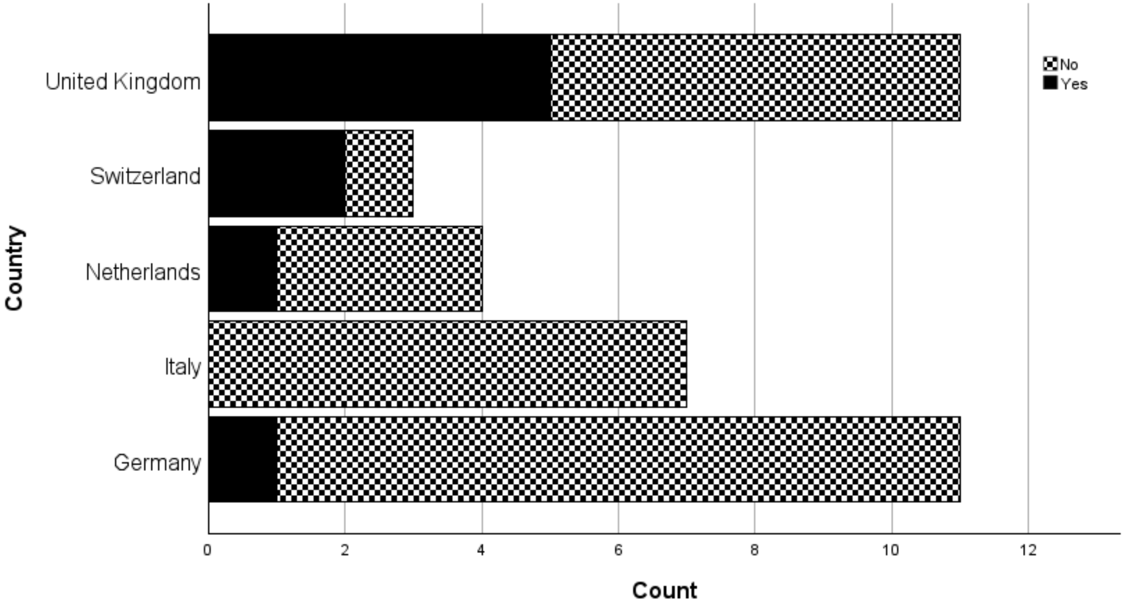

Supplemental Figure 6D - Recommended interval for thoracic screening

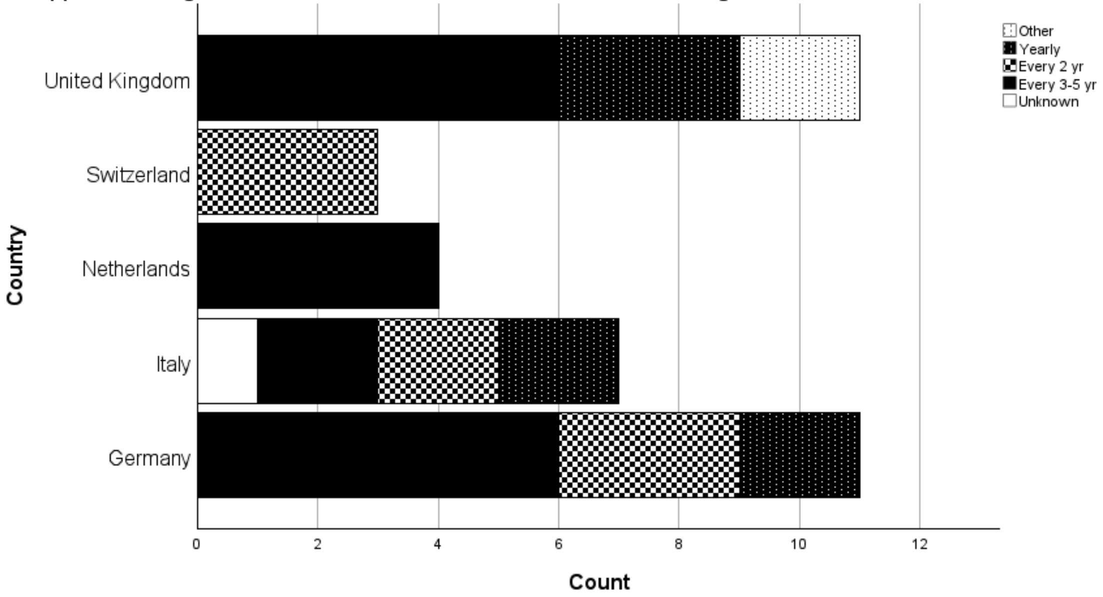


Supplemental Figure 6E - Adult - Recommended frequency for surveillance of small lungNETs

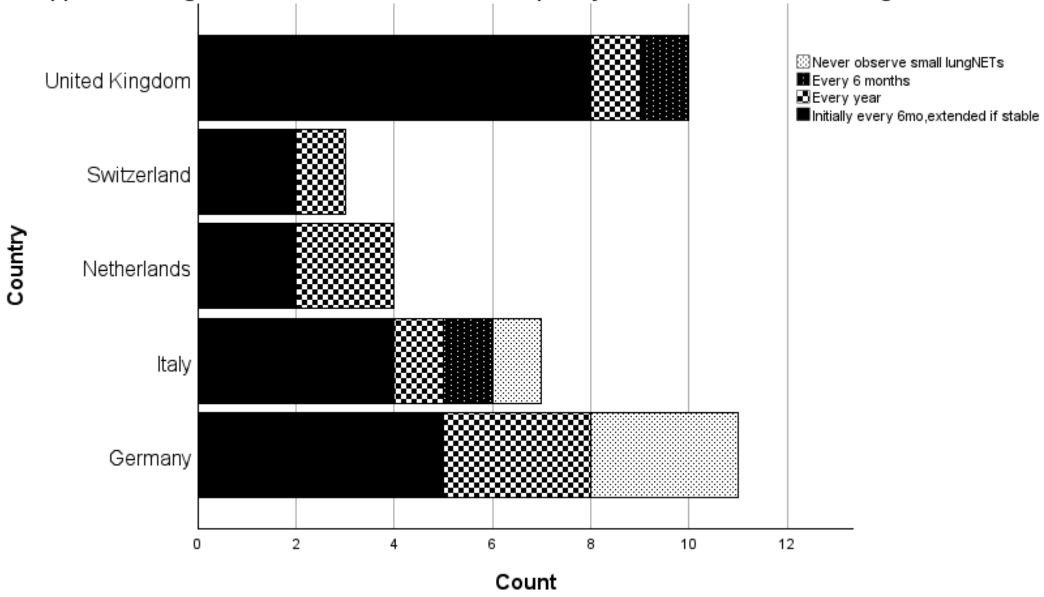

# Supplemental Figure 7

Advice for periodical follow-up in GN-MEN1 by country – for countries from which 3 or more CoEs provided answers

Supplemental Figure 7A - Recommended periodic follow-up in patients with GN-MEN1

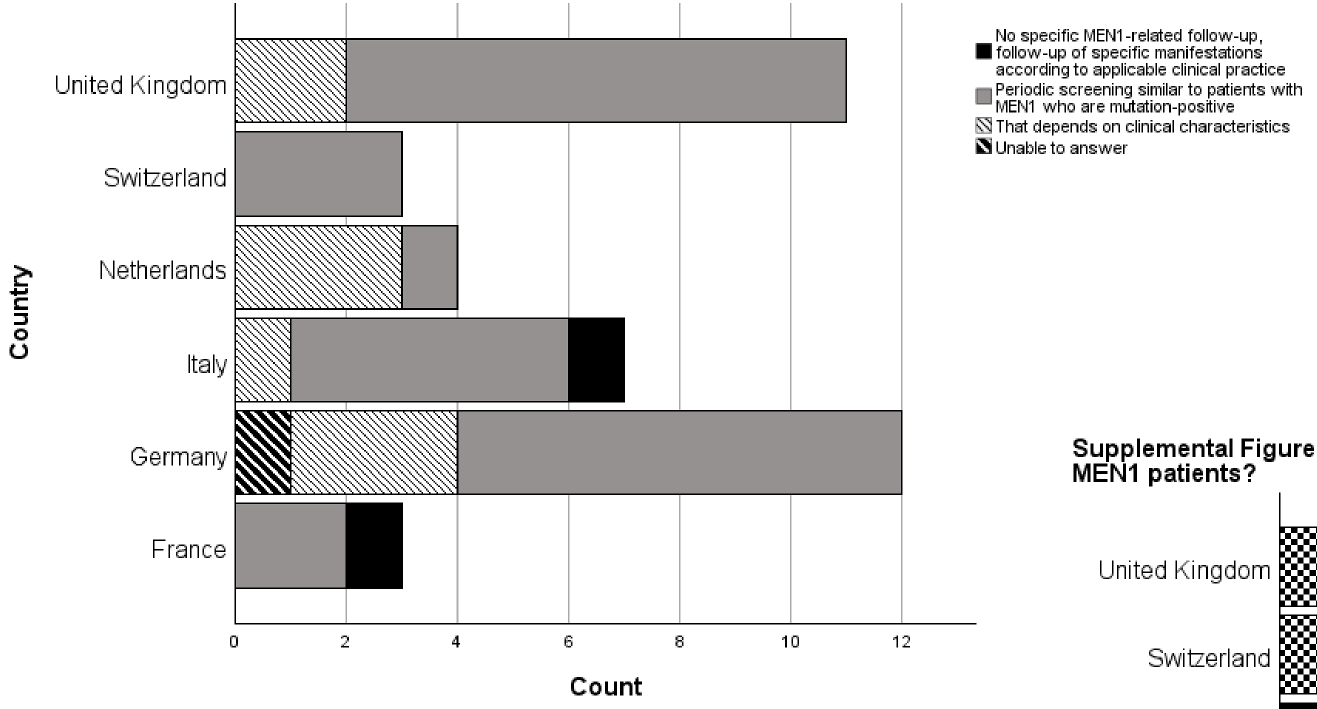


Supplemental Figure 7B - Would you recommend periodic follow-up for 1st degree family members of GN-MEN1 patients?

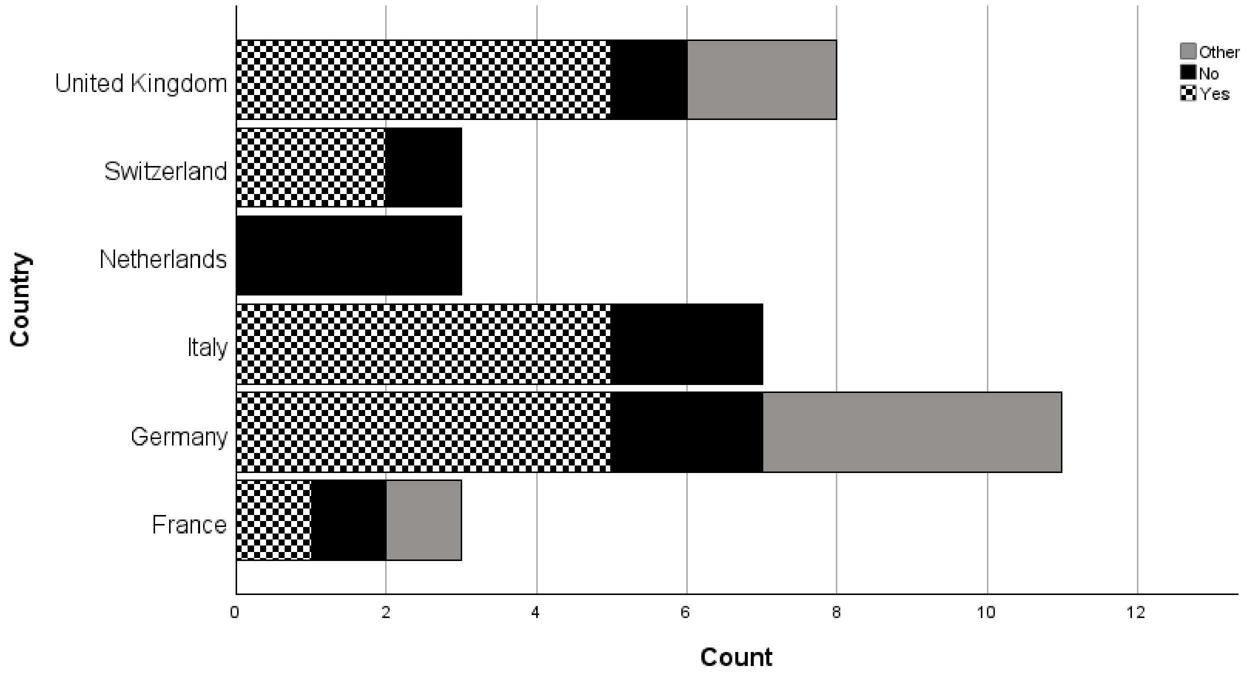

Supplement: Supplementary file 1 — Data S1. Supporting Information Figures. [file JNE-37-e13468-s001.pdf]
