# Supplementary material for: Screening and surveillance practices for Multiple Endocrine Neoplasia type 1‐related Neuroendocrine Tumours in European Neuroendocrine Tumor Society Centers of Excellence (ENETS CoE)—An ENETS MEN1 task force questionnaire study
Source: J Neuroendocrinol. 2024 Nov 26;37(1):e13468. doi: 10.1111/jne.13468 (PMC11750319; doi:10.1111/jne.13468)
Supplement: Supplementary file 2 — Data S2. Supporting Information. [file JNE-37-e13468-s002.pdf]

Surveys of ENETS MEN1 TF - Clinical Practice Questionnaire - version 43.21

Printed on 22-12-2022 12:37:16 by Carolina Rebecca Christina Pieterman

Survey 'MEN1 - Clinical Practice Questionnaire - I'

MEN1 - Clinical Practice Questionnaire - I - Introduction

DEAR COLLEAGUE,

Welcome and thank you for participating in the Clinical Practice Questionnaire of the ENETS Multiple Endocrine Neoplasia Type I (MEN1) Task Force.

We request that this questionnaire is filled out by the lead provider coordinating MEN1 care in each institute, on behalf of the entire team and institute.

We encourage the lead provider to consult with specific members of the team if necessary to answer the questions.

Logistics of the questionnaire

The questionnaire is composed of 4 steps and the questions of each step need to be completed before you can advance to the next step.

You can return to a previous step either by using the button “previous” in the lower right-hand corner of the screen, or by using the step navigator on the left.

You can use the cogwheel to the right of each question to clear your answer if necessary.

For multiple choice questions radio buttons (round) indicate only one answer is possible, while checkboxes (square) indicate that multiple options may be checked.

You do not need to save your answers, they are saved automatically upon entry.

Content of the questionnaire

- Step 1 deals with organization of MEN1 care in your institution.
- Step 2 deals with the follow-up of mutation-negative patients with clinical MEN1. This step starts with a brief case vignette to which the questions apply.
- Step 3 deals with practices regarding periodical screening in children.
- Step 4 deals with practices regarding periodical screening in adults.

Time indication

If you indicate in the first step that no children and/or adults are seen some of the next steps may not be applicable anymore and you can click through these steps with next and finish the questionnaire.

The entire questionnaire takes about 15 minutes to fill out.

Thanks again for your participation, and please contact us if you have any questions,

On behalf of the ENETS MEN1 Task Force,

Carla Pieterman | email: c.r.c.pieterman@umcutrecht.nl | Phone: +31 88 75 603 90

Gregory Kaltsas

Gerlof Valk

MEN1 - Clinical Practice Questionnaire - I - 1. Organization of MEN1 Care

Number	Question	Answers
	Who is filling out the Questionnaire	
1.2	Please select your institute from the dropdown list	◯ Peter MacCALLUM CANCER CENTER<br>◯ Sydney Theranostics (North)<br>◯ Medical University Vienna<br>◯ University Hospital Gasthuisberg-KU Leuven<br>◯ NETwerk<br>◯ Neuroendocrine Tumor Center of Excellence Rigshospitalet, University of Copenhagen, Depart. PE<br>◯ Aarhus University Hospital ENETS Center of Excellence<br>◯ Hôpital Edouard Herriot<br>◯ Dept of Gastroenterology-Pancreatology Beaujon Hospital and Paris Diderot University Clichy<br>◯ IPC Neuroendocrine Tumor Center<br>◯ Universitary Center of Excellence for Neuroendocrine Tumors Toulouse<br>◯ ENETS CoE - Charité, Universitätsmedizin Berlin, Campus Virchow-Klinikum<br>◯ GEPNET-KUM - Interdisziplinäres Zentrum für Neuroendokrine Tumoren des GastroEnteroPankreatischen Systems des Klinikums der Universität München<br>◯ NET Zentrum Marburg<br>◯ Zentralklinik Bad Berka GmbH<br>◯ Zentrum für Neuroendokrine Tumore, Westdeutsches Tumorzentrum Essen, Klinik für Endokrinologie, Diabetologie und Stoffwechsel, Universitätsklinikum Essen<br>◯ IENET am UCT Universitätsmedizin der Johannes Gutenberg Universität Mainz<br>◯ Zentrum für Neuroendokrine Tumore, Klinikum rechts der Isar<br>◯ Interdisziplinäres Zentrum für Neuroendokrine Tumoren Würzburg<br>◯ NET Centrum Hamburg<br>◯ University hospital of cologne<br>◯ NET-Zentrum UKSH<br>◯ NET Center Tübingen<br>◯ EKPA-LAIKO CENTER<br>◯ Department of Internal Medicine and Oncology Semmelweis University

◯ Neuroendocrine Tumour Centre - St Vincent's Universtiy Hospital
◯ Hadassah-Hebrew University Medical Center
◯ National Cancer Institute of Milan
◯ Humanitas Research Hospital
◯ Multidisciplinary Group for Neuroendocrine Tumors of Naples
◯ NET Center Roma Policlinico Agostino Gemelli - Policlinico Sant'Andrea
◯ Multidisciplinary Group of Verona for Neuroendocrine Tumors
◯ European Institute of Oncology
◯ NET BOLOGNA Multidisciplinary Team
◯ San Raffaele Hospital Neuroendocrine Tumor Group
◯ ENETS centre of excellence Erasmus MC Rotterdam
◯ Antoni van Leeuwenhoek/UMC Utrecht, centrum voor neuro-endocriene tumoren
◯ Amsterdam University Medical Centers
◯ University Medical Center Groningen
◯ Center for neuroendocrine tumors, Oslo
◯ ENETS Center of Excellence, Department of Endocrinology and Neuroendocrine Tumor, Medical University of Silesia, Katowice
◯ Maria Sklodowska-Curie Memorial Cancer Centre and Institute of Oncology
◯ Vall Hebron Institute of Hospital Universitari Oncoloy
◯ Department of Endocrine Oncology Uppsala University Hospital
◯ University Hospital of Zurich
◯ Centre des tumeurs neuroendocrines, Lausanne
◯ Inselspital, Bern
◯ Universitätsspital Basel - TZ NET
◯ The Christie Neuroendocrine Centre of Excellence, Manchester
◯ Liverpool Regional NET Service
◯ Royal Free Hospital, London
◯ Imperial College Healthcare NHS Trust, London
◯ Oxford University Hospitals NHS Trust
◯ King's Health Partners (Kings College Hospital (lead site), Guys, St Thomas, Kent, North Hampshire Hospital)
◯ Birmingham Neuroendocrine Tumour Centre
◯ Sheffield Teaching Hospitals ENETS Centre of Excellence
◯ Wessex Neuroendocrine Tumour Group

		◯ The ARDEN NET Centre, University Hospitals Coventry & Warwickshire NHS Trust, WISDEM centre<br>◯ West of Scotland NET Centre<br>◯ Newcastle Freeman Hospital NET Centre of Excellence<br>◯ Belfast NET Centre<br>◯ University of Iowa Neuroendocrine Tumor Center of Excellence<br>◯ Other<br>◯ South Wales Neuroendocrine Cancer Service<br>◯ Universitätsklinikum Erlangen; Medizinische Klinik 1, M1 NET-Ambulanz<br>◯ Hospital General Universitario Gregorio Marañón / Hospital Universitario Infanta Sofia Madrid
1.3	***If 'Please select your institute from the dropdown list' is equal to 'Other' answer this question:***<br>Other institute	
1.4	Please select the Specialty of the person filling out the questionnaire on behalf of the Institute	◯ Endocrinologist<br>◯ Gastroenterologist<br>◯ Medical Oncologist<br>◯ Surgeon<br>◯ Other
1.5	***If 'Please select the Specialty of the person filling out the questionnaire on behalf of the Institute' is equal to 'Surgeon' answer this question:***<br>For Surgeons, please enter your subspecialty	
1.6	***If 'Please select the Specialty of the person filling out the questionnaire on behalf of the Institute' is equal to 'Other' answer this question:***<br>Please enter your specialty in the text field	
	Organization of Care - Adult patients	
1.8	Are adults with MEN1 managed at your institution?	◯ No<br>◯ Yes, we see patients for diagnosis, screening, surveillance, treatment and follow-up<br>◯ Yes, but only for specific diagnostic tests/ treatments. Screening, surveillance and follow-up is done elsewhere<br>◯ Other
1.9	***If 'Are adults with MEN1 managed at your institution?' is equal to 'Yes, but only for specific diagnostic tests/ treatments. Screening, surveillance and follow-up is done elsewhere' answer this question:***<br>Please specify for which diagnostic tests and/or treatments _adults_ with MEN1 are seen at your insitution	

1.10	***If 'Are adults with MEN1 managed at your institution?' is equal to 'Other' answer this question:***<br>Please specify what you mean by "other"	
1.11	***If 'Are adults with MEN1 managed at your institution?' is equal to 'Yes, we see patients for diagnosis, screening, surveillance, treatment and follow-up' answer this question:***<br>Which specialty provides the periodical screening and regular follow-up of adults with MEN1 at your institution? That is, which specialty is the coordinating provider for patients with MEN1. Please select all that apply.	☐ Endocrinologist<br>☐ Gastroenterologist<br>☐ Medical Oncologist<br>☐ Surgeon<br>☐ Other
1.12	***If 'Which specialty provides the periodical screening and regular follow-up of adults with MEN1 at your institution? That is, which specialty is the coordinating provider for patients with MEN1. Please select all that apply.' is equal to 'Surgeon' answer this question:***<br>For Surgeons, please enter the subspecialt(y)/(ies)	
1.13	***If 'Which specialty provides the periodical screening and regular follow-up of adults with MEN1 at your institution? That is, which specialty is the coordinating provider for patients with MEN1. Please select all that apply.' is equal to 'Other' answer this question:***<br>Please enter other specialt(y)/(ies) in the text field	
1.14	***If 'Are adults with MEN1 managed at your institution?' is equal to 'Yes, we see patients for diagnosis, screening, surveillance, treatment and follow-up' answer this question:***<br>How many adults currently receive their regular MEN1 screening and follow-up at your institution? That is, the number of adults with MEN1 for whom your institution is the lead provider in their MEN1 care at the time of filling out the questionnaire.	◯ <10<br>◯ 10-49<br>◯ 50-100<br>◯ >100<br>◯ Unknown/Unable to Answer
	Organization of Care - Children	
1.16	Are children with MEN1 managed at your institution?	◯ No<br>◯ Yes, we see children for diagnosis, screening, surveillance, treatment and follow-up<br>◯ Yes, but only for specific diagnostic tests/ treatments. Screening, surveillance and follow-up is done elsewhere<br>◯ Other
1.17	***If 'Are children with MEN1 managed at your institution?' is equal to 'Yes, but only for specific diagnostic tests/ treatments. Screening, surveillance and follow-up is done elsewhere' answer this question:***<br>Please specify for which diagnostic tests and/or treatments *children* with MEN1 are seen at your insitution	

1.18	***If 'Are children with MEN1 managed at your institution?' is equal to 'Other' answer this question:***<br>Please specify what you mean by "other"	
1.19	***If 'Are children with MEN1 managed at your institution?' is equal to 'Yes, we see children for diagnosis, screening, surveillance, treatment and follow-up' answer this question:***<br>Which specialty provides the periodical screening and regular follow-up of children with MEN1 at your institution? That is, which specialty is the coordinating provider for children with MEN1. Please select all that apply.	☐ Pediatric Endocrinologist<br>☐ Pediatric Oncologist<br>☐ General Pediatrician<br>☐ Adult Endocrinologist<br>☐ Adult Oncologist<br>☐ Other
1.20	***If 'Which specialty provides the periodical screening and regular follow-up of children with MEN1 at your institution? That is, which specialty is the coordinating provider for children with MEN1. Please select all that apply.' is equal to 'Other' answer this question:***<br>Please enter other specialt(y)/(ies) in the text field	
1.21	***If 'Are children with MEN1 managed at your institution?' is equal to 'Yes, we see children for diagnosis, screening, surveillance, treatment and follow-up' answer this question:***<br>How many children with MEN1 receive their regular MEN1 screening and follow-up at your institution? That is, the number of children with MEN1 for whom your institution is the lead provider in their MEN1 care at the time of filling out the questionnaire.	◯ <10<br>◯ 10-25<br>◯ >25<br>◯ Unknown/Unable to answer
1.22	Seeing MEN1 pts (either adults or children) for regular surveillance	
	Institutional MEN1 protocol	
1.24	***If 'Seeing MEN1 pts (either adults or children) for regular surveillance' is equal to 'Yes' answer this question:***<br>Do you have an institutional protocol for management and follow-up of patients with MEN1 used by all providers of MEN1 patient care at your institution?	◯ No<br>◯ Yes
	Genetic Counseling	
1.26	***If 'Seeing MEN1 pts (either adults or children) for regular surveillance' is equal to 'Yes' answer this question:***<br>Who provides genetics counseling for patients with or suspected of MEN1?	◯ A clinical geneticist / genetic counselor at our institution<br>◯ A clinical geneticist / genetic counselor outside the institution<br>◯ The provider of the periodical screening and regular follow-up<br>◯ Other
1.27	***If 'Who provides genetics counseling for patients with or suspected of MEN1?' is equal to 'Other' answer this question:***<br>Please specify who provides genetic counseling	

	Research	
1.29	***If 'Seeing MEN1 pts (either adults or children) for regular surveillance' is equal to 'Yes' answer this question:***<br>Does your institution have a clinical research database for MEN1?	◯ No<br>◯ Yes<br>◯ Unknown/Unable to answer
1.30	***If 'Seeing MEN1 pts (either adults or children) for regular surveillance' is equal to 'Yes' answer this question:***<br>Are blood and/or tissue from patients with MEN1 included in a research biobank at your institution?	◯ No<br>◯ Yes<br>◯ Unknown/Unable to answer

MEN1 - Clinical Practice Questionnaire - I - 2. Clinical MEN1/Mutation-Negative

Number	Question	Answers
2.1	***If 'Seeing MEN1 pts (either adults or children) for regular surveillance' is not equal to 'Yes' answer this question:*** Previous answer to: Are adults with MEN1 managed at your institution?	
2.2	***If 'Seeing MEN1 pts (either adults or children) for regular surveillance' is not equal to 'Yes' answer this question:*** Previous answer to: Are children with MEN1 managed at your institution?	
	The following questions are only applicable if either adults or children with MEN1 are seen at your insitution for regular surveillance and follow-up. If you have indicated this is not the case, the questions will not be visible and you can select "next" to go to the next step	
	The following questions pertain to the case-vignette described below. **PLEASE READ IT CAREFULLY BEFORE ANSWERING THE QUESTIONS**	
	___Case-Vignette___	
	A patient is diagnosed with **clinical MEN1** based on a personal history of **two of the three main manifestations** of MEN1 (main manifestations are: Primary Hyperparathyroidism (pHPT), Pituitary Adenoma (PA), and neuroendocrine tumors (NETs) of the pancreas and/or gastrinoma). The patient has **no other manifestations of the syndrome** and there are **no family members with MEN1**. **Comprehensive, state-of-the-art genetic testing is negative** for MEN1 or any other relevant germline mutation.	
2.7	***If 'Seeing MEN1 pts (either adults or children) for regular surveillance' is equal to 'Yes' answer this question:*** What would the advice of your institution be for follow up of this patient?	○ No specific MEN1-related follow-up, follow-up for his/her specific manifestations according to applicable clinical practice<br>○ Periodical screening similar to patients with MEN1 who are mutation-positive<br>○ That depends on clinical characteristics<br>○ Unable to answer
2.8	***If 'What would the advice of your institution be for follow up of this patient?' is equal to 'That depends on clinical characteristics' answer this question:*** Is the decision for follow-up of the patient dependent on the age of the patient?	○ No<br>○ Yes<br>○ Unknown/Unable to answer
2.9	***If 'Is the decision for follow-up of the patient dependent on the age of the patient?' is equal to 'Yes' answer this question:*** If the decision is age-depended, at which age cut-off would your institution advice periodical screening similar to mutation-positive patients?	○ Below the age of 30<br>○ Below the age of 50<br>○ Other<br>○ Unable to answer
2.10	***If 'If the decision is age-depended, at which age cut-off would your institution advice periodical screening similar to mutation-positive patients?' is equal to 'Other' answer this question:*** Please specify the age cut-off	

2.11	***If 'What would the advice of your institution be for follow up of this patient?' is equal to 'That depends on clinical characteristics' answer this question:***<br>Is the decision for follow-up of the patient dependent on the combination of manifestations that make up the diagnosis of clinical MEN1?	○ No<br>○ Yes<br>○ Unknown/Unable to answer
2.12	***If 'Is the decision for follow-up of the patient dependent on the combination of manifestations that make up the diagnosis of clinical MEN1?' is equal to 'Yes' answer this question:***<br>For which combination of manifestations would your institution advice periodical screening similar to mutation-positive patients? Please select all that apply	☐ Primary hyperparathyroidism and Pituitary Adenoma<br>☐ Primary hyperparathyroidism and PanNET or gastrinoma<br>☐ Pituitary Adenoma and PanNET or gastrinoma<br>☐ Unable to answer
2.13	***If 'What would the advice of your institution be for follow up of this patient?' is equal to 'That depends on clinical characteristics' answer this question:***<br>Is the decision for follow-up of the patient dependent on a family history of MEN1-related tumors?	○ No<br>○ Yes<br>○ Unknown/Unable to answer
2.14	***If 'Is the decision for follow-up of the patient dependent on a family history of MEN1-related tumors?' is equal to 'Yes' answer this question:***<br>To which degree would family history be considered in the decision for follow-up of the patient?	○ First-degree only (parents, children, full siblings)<br>○ Up to second-degree (aunts, uncles, grandparents, grandchildren, nieces, nephews, or half-siblings)<br>○ Further removed<br>○ Unable to answer
2.15	***If 'Seeing MEN1 pts (either adults or children) for regular surveillance' is equal to 'Yes' answer this question:***<br>Would your institution advice periodical screening for first-degree relatives of this patient?	○ No<br>○ Yes<br>○ Other<br>○ Unable to answer
2.16	***If 'Would your institution advice periodical screening for first-degree relatives of this patient?' is equal to 'Other' answer this question:***<br>Please specify your answer	

MEN1 - Clinical Practice Questionnaire - I - 3. Periodical Screening in Children

Number	Question	Answers
3.1	***If 'Are children with MEN1 managed at your institution?' is not equal to 'Yes, we see children for diagnosis, screening, surveillance, treatment and follow-up' answer this question:***<br>Previous answer to: Are children with MEN1 managed at your institution?	
	The following questions pertain to periodical screening at the pediatric age. If you have indicated earlier that children are not seen at your institution for regular screening, surveillance and follow-up, the questions will not be visible and you can select "next" to go to the next step	
	The following questions pertain to **periodical screening at the ___PEDIATRIC___ age**.	
3.4	***If 'Are children with MEN1 managed at your institution?' is equal to 'Yes, we see children for diagnosis, screening, surveillance, treatment and follow-up' answer this question:***<br>In a family with known MEN1, what is the advised age to perform predictive genetic testing (that is, testing the child for the familial MEN1 mutation) at your institution	◯ <5 years<br>◯ 5-10 years<br>◯ 11-18 years<br>◯ >18 years<br>◯ Other<br>◯ Unknown/ unable to answer
3.5	***If 'In a family with known MEN1, what is the advised age to perform predictive genetic testing (that is, testing the child for the familial MEN1 mutation) at your institution' is equal to 'Other' answer this question:***<br>Please specify advised age to perform predictive genetic testing	[ ]
3.6	***If 'Are children with MEN1 managed at your institution?' is equal to 'Yes, we see children for diagnosis, screening, surveillance, treatment and follow-up' answer this question:***<br>What is the generally recommended age at your institution to initiate clinical screening in children with MEN1 (i.e. visits to the clinic)	◯ <5 years<br>◯ 5-10 years<br>◯ 11-18 years<br>◯ >18 years<br>◯ Other<br>◯ Unknown/ unable to answer
3.7	***If 'What is the generally recommended age at your institution to initiate clinical screening in children with MEN1 (i.e. visits to the clinic)' is equal to 'Other' answer this question:***<br>Please specify advised age to initiate clinical screening	[ ]
3.8	***If 'What is the generally recommended age at your institution to initiate clinical screening in children with MEN1 (i.e. visits to the clinic)' is not equal to '>18 years' answer this question:***<br>For pediatric patients with MEN1 (0-19) without manifestations, what is the recommended frequency of clinic visits at your institution?	◯ Every six months<br>◯ Every year<br>◯ Every two years<br>◯ Other<br>◯ Unkown/unable to answer

3.9	***If 'For pediatric patients with MEN1 (0-19) without manifestations, what is the recommended frequency of clinic visits at your institution?' is equal to 'Other' answer this question:***<br>Please specify other frequency	__________
3.10	***If 'Are children with MEN1 managed at your institution?' is equal to 'Yes, we see children for diagnosis, screening, surveillance, treatment and follow-up' answer this question:***<br>What is the generally recommended age at your institution to initiate abdominal radiological screening in children with MEN1, if the child is otherwise asymptomatic?	○ <5 years<br>○ 5-10 years<br>○ 11-18 years<br>○ >18 years<br>○ Other<br>○ Unknown/ unable to answer
3.11	***If 'What is the generally recommended age at your institution to initiate abdominal radiological screening in children with MEN1, if the child is otherwise asymptomatic?' is equal to 'Other' answer this question:***<br>Please specify advised age to initiate abdominal radiological screening	__________
3.12	***If 'What is the generally recommended age at your institution to initiate abdominal radiological screening in children with MEN1, if the child is otherwise asymptomatic?' is not equal to '>18 years' answer this question:***<br>What is the preferred imaging modality used at your institution for abdominal screening in pediatric patients with MEN1? Please check all that apply	☐ CT<br>☐ MRI<br>☐ EUS<br>☐ 68-gallium-dotatate(toc/noc)-PET/CT<br>☐ Other<br>☐ Unknown/Unable to answer
3.13	***If 'What is the preferred imaging modality used at your institution for abdominal screening in pediatric patients with MEN1? Please check all that apply' is equal to 'Other' answer this question:***<br>Please specify preferred imaging modality	__________
3.14	***If 'What is the generally recommended age at your institution to initiate abdominal radiological screening in children with MEN1, if the child is otherwise asymptomatic?' is not equal to '>18 years' answer this question:***<br>If the initial abdominal radiological screening is negative, at which interval would repeat abdominal imaging be recommended, given absence of signs and symptoms?	○ 1 year<br>○ 2 years<br>○ 3 years<br>○ 5 years<br>○ Other<br>○ Unknown/unable to answer
3.15	***If 'If the initial abdominal radiological screening is negative, at which interval would repeat abdominal imaging be recommended, given absence of signs and symptoms?' is equal to 'Other' answer this question:***<br>Please specify repeat interval	__________

3.16 ***If 'Are children with MEN1 managed at your institution?' is equal to 'Yes, we see children for diagnosis, screening, surveillance, treatment and follow-up' answer this question:***
What is the generally recommended age at your institution to initiate thoracic radiological screening in children with MEN1, if the child is otherwise asymptomatic?

- ○ <5 years
- ○ 5-10 years
- ○ 11-18 years
- ○ >18 years
- ○ Other
- ○ Unknown/ unable to answer

3.17 ***If 'What is the generally recommended age at your institution to initiate thoracic radiological screening in children with MEN1, if the child is otherwise asymptomatic?' is equal to 'Other' answer this question:***
Please specify advised age to initiate thoracic radiological screening

---

3.18 ***If 'What is the generally recommended age at your institution to initiate thoracic radiological screening in children with MEN1, if the child is otherwise asymptomatic?' is not equal to '>18 years' answer this question:***
What is the preferred imaging modality used at your institution for thoracic radiological screening in pediatric patients? Please check all that apply.

- ☐ CT
- ☐ MRI
- ☐ 68-gallium-dotatate(toc/noc)-PET/CT
- ☐ Other
- ☐ Unknown/unable to answer

3.19 ***If 'What is the preferred imaging modality used at your institution for thoracic radiological screening in pediatric patients? Please check all that apply.' is equal to 'Other' answer this question:***
Please specify preferred imaging modality

3.20 ***If 'What is the generally recommended age at your institution to initiate thoracic radiological screening in children with MEN1, if the child is otherwise asymptomatic?' is not equal to '>18 years' answer this question:***
If the initial thoracic screening is negative, at which interval would repeat thoracic imaging be recommended, given absence of signs and symptoms?

- ○ 1 year
- ○ 2 years
- ○ 3 years
- ○ 5 years
- ○ Other
- ○ Unknown/unable to answer

3.21 ***If 'If the initial thoracic screening is negative, at which interval would repeat thoracic imaging be recommended, given absence of signs and symptoms?' is equal to 'Other' answer this question:***
Please specify repeat interval

The next three questions pertain to periodical biochemical screening in children. What is the standard set of biochemical tests performed in a child with MEN1 (without symptoms or manifestations)? Please check all that are included in the standard set at your institution.

3.23	***If 'Are children with MEN1 managed at your institution?' is equal to 'Yes, we see children for diagnosis, screening, surveillance, treatment and follow-up' answer this question:***<br>If the child is 5 years old	☐ None<br>☐ Calcium<br>☐ PTH<br>☐ Fasting Gastrin<br>☐ Fasting Insulin<br>☐ Fasting Glucose<br>☐ Fasting CgA<br>☐ Fasting Glucagon<br>☐ Fasting PPT<br>☐ Fasting VIP<br>☐ IGF-1<br>☐ Prolactin<br>☐ Other<br>☐ Unknown/ unable to answer
3.24	***If 'If the child is 5 years old' is equal to 'Other' answer this question:***<br>Please specify other biochemical tests at 5 years of age	__________
3.25	***If 'Are children with MEN1 managed at your institution?' is equal to 'Yes, we see children for diagnosis, screening, surveillance, treatment and follow-up' answer this question:***<br>If the child is 10 years old	☐ None<br>☐ Calcium<br>☐ PTH<br>☐ Fasting Gastrin<br>☐ Fasting Insulin<br>☐ Fasting Glucose<br>☐ Fasting CgA<br>☐ Fasting Glucagon<br>☐ Fasting PPT<br>☐ Fasting VIP<br>☐ IGF-1<br>☐ Prolactin<br>☐ Other<br>☐ Unknown/ unable to answer
3.26	***If 'If the child is 10 years old' is equal to 'Other' answer this question:***<br>Please specify other biochemical tests at 10 years of age	__________

3.27	***If 'Are children with MEN1 managed at your institution?' is equal to 'Yes, we see children for diagnosis, screening, surveillance, treatment and follow-up' answer this question:***<br>If the child is 16 years old	☐ None<br>☐ Calcium<br>☐ PTH<br>☐ Fasting Gastrin<br>☐ Fasting Insulin<br>☐ Fasting Glucose<br>☐ Fasting CgA<br>☐ Fasting Glucagon<br>☐ Fasting PPT<br>☐ Fasting VIP<br>☐ IGF-1<br>☐ Prolactin<br>☐ Other<br>☐ Unknown/ unable to answer
3.28	***If 'If the child is 16 years old' is equal to 'Other' answer this question:***<br>Please specify other biochemical tests at 16 years of age	______________

MEN1 - Clinical Practice Questionnaire - I - 4. Periodical Screening in Adults

Number	Question	Answers
	The following questions pertain to **periodical screening in ___ADULTS___ with MEN1**.	
4.2	***If 'Are adults with MEN1 managed at your institution?' is not equal to 'Yes, we see patients for diagnosis, screening, surveillance, treatment and follow-up' answer this question:***<br>Previous answer to: Are adults with MEN1 managed at your institution?	
	The following questions pertain to periodical screening in adults with MEN1. If you have indicated earlier that adults with MEN1 are not seen at your institution for screening, surveillance and follow-up, the questions will not be visible and you can now finish the questionnaire.	
	Biochemical Screening	
4.5	***If 'Are adults with MEN1 managed at your institution?' is equal to 'Yes, we see patients for diagnosis, screening, surveillance, treatment and follow-up' answer this question:***<br>For periodical screening, what is the standard set of biochemical tests performed in adults with MEN1 (without symptoms or manifestations)? Please check all that are included in the standard set at your institution.	☐ Calcium<br>☐ Ionized Calcium<br>☐ PTH<br>☐ Fasting Gastrin<br>☐ Fasting Insulin<br>☐ Fasting Glucose<br>☐ Fasting CgA<br>☐ Fasting Glucagon<br>☐ Fasting Pancreatic Polypeptide<br>☐ Fasting VIP<br>☐ IGF-1<br>☐ Prolactin<br>☐ LH/FSH<br>☐ Estradiol<br>☐ Testosterone<br>☐ TSH<br>☐ Free T4<br>☐ Cortisol<br>☐ Other<br>☐ Unknown/ Unable to answer
4.6	***If 'For periodical screening, what is the standard set of biochemical tests performed in adults with MEN1 (without symptoms or manifestations)? Please check all that are included in the standard set at your institution.' is equal to 'Other' answer this question:***<br>Please specify other biochemcial tests included in the standard set at your institution	
	Biochemical diagnosis of gastrinoma and Esophagogastroduodenoscopy (EGD)	

4.8	***If 'Are adults with MEN1 managed at your institution?' is equal to 'Yes, we see patients for diagnosis, screening, surveillance, treatment and follow-up' answer this question:***<br>Which biochemical modalities are generally used for gastrinoma diagnosis in patients with MEN1 at your institution? Please check all that apply.	☐ Fasting Serum Gastrin<br>☐ Secretin Test<br>☐ Gastric pH<br>☐ Basal acid output<br>☐ Calcium stimulation Test<br>☐ Other<br>☐ Unknown/ Unable to answer
4.9	***If 'Which biochemical modalities are generally used for gastrinoma diagnosis in patients with MEN1 at your institution? Please check all that apply.' is equal to 'Other' answer this question:***<br>Please specify other biochemical modalities used for gastrinoma diagnosis in MEN1	
4.10	***If 'Are adults with MEN1 managed at your institution?' is equal to 'Yes, we see patients for diagnosis, screening, surveillance, treatment and follow-up' answer this question:***<br>Is esophagogastroduodenoscopy (EGD) routinely performed in adults with MEN1 at your institution?	○ No<br>○ Yes, in all patients with MEN1<br>○ Yes, but only in patients with hypergastrinemia<br>○ Unknown/ unable to answer
4.11	***If 'Is esophagogastroduodenoscopy (EGD) routinely performed in adults with MEN1 at your institution?' is equal to 'Yes, in all patients with MEN1' answer this question:***<br>For what purpose do you use EGD in ___all adults with MEN1___? Please select all that apply.	☐ To screen for duodenal NETs<br>☐ To screen for gastric NETs<br>☐ To screen for peptic ulcer disease<br>☐ Other<br>☐ Unknown/Unable to answer
4.12	***If 'For what purpose do you use EGD in all adults with MEN1? Please select all that apply.' is equal to 'Other' answer this question:***<br>Please specify other purpose for EGD in adults with MEN1	
4.13	***If 'Is esophagogastroduodenoscopy (EGD) routinely performed in adults with MEN1 at your institution?' is equal to 'Yes, but only in patients with hypergastrinemia' answer this question:***<br>For what purpose do you use EGD in ___patients with MEN1 with hypergastrinemia___? Please select all that apply	☐ To screen for duodenal NETs<br>☐ Surveillance of duodenal NETs<br>☐ To screen for gastric NETs<br>☐ Surveillance of gastric NETs<br>☐ To monitor peptic ulcer disease<br>☐ To fine-tune PPI<br>☐ Other<br>☐ Unknown/ unable to answer
4.14	***If 'For what purpose do you use EGD in patients with MEN1 with hypergastrinemia? Please select all that apply' is equal to 'Other' answer this question:***<br>Please specify other purpose for EGD in adults with MEN1 with hypergastrinemia	
	EUS and EUS-guided FNA/FNB for pancreatic NETs	

4.16	***If 'Are adults with MEN1 managed at your institution?' is equal to 'Yes, we see patients for diagnosis, screening, surveillance, treatment and follow-up' answer this question:***<br>How is Endoscopic Ultrasound (EUS) used at your institution in the screening and surveillance of pancreatic NETs in patients with MEN1. Please check all that apply.	☐ EUS is not available at our institution<br>☐ EUS is not used in patients with MEN1<br>☐ EUS is routinely used for screening in patients without PanNETs<br>☐ EUS is routinely used for surveillance in patients with PanNETs<br>☐ EUS is used on specific indication<br>☐ Other<br>☐ Unknown/ unable to answer
4.17	***If 'How is Endoscopic Ultrasound (EUS) used at your institution in the screening and surveillance of pancreatic NETs in patients with MEN1. Please check all that apply.' is equal to 'EUS is used on specific indication' answer this question:***<br>Please specify for which specific indication(s) EUS would be used at your institution	
4.19	***If 'How is Endoscopic Ultrasound (EUS) used at your institution in the screening and surveillance of pancreatic NETs in patients with MEN1. Please check all that apply.' is equal to 'Other' answer this question:***<br>Please specify other use of EUS in MEN1 at your institution	
4.20	***If 'Are adults with MEN1 managed at your institution?' is equal to 'Yes, we see patients for diagnosis, screening, surveillance, treatment and follow-up' answer this question:***<br>Is EUS-guided Fine Needle Aspiration (FNA) or fine needle biopsy (FNB) ___routinely___ used in patients with MEN1 at your institution?	○ No, never done<br>○ Yes, generally done each time EUS is peformed<br>○ On specific indication only<br>○ Other<br>○ Unknown/ Unable to answer
4.21	***If 'Is EUS-guided Fine Needle Aspiration (FNA) or fine needle biopsy (FNB) routinely used in patients with MEN1 at your institution?' is equal to 'On specific indication only' answer this question:***<br>Please specify for which specific indication(s) FNA/FNB is used	
4.22	***If 'Is EUS-guided Fine Needle Aspiration (FNA) or fine needle biopsy (FNB) routinely used in patients with MEN1 at your institution?' is equal to 'Other' answer this question:***<br>Please specify what you mean by other	
	Somatostatin receptor PET-CT	

4.24	***If 'Are adults with MEN1 managed at your institution?' is equal to 'Yes, we see patients for diagnosis, screening, surveillance, treatment and follow-up' answer this question:***<br>How is 68-gallium-dotatate(toc/noc)-PET/CT used at your institution in patients with MEN1? Please check all that apply.	☐ Not used<br>☐ Regular use in screening of patients without NETs<br>☐ Regular use in surveillance of prevalent non-metastatic NETs<br>☐ Regular use in surveillance of metastatic NETs<br>☐ On specific indication only<br>☐ Unknown/ Unable to answer
4.25	***If 'How is 68-gallium-dotatate(toc/noc)-PET/CT used at your institution in patients with MEN1? Please check all that apply.' is equal to 'On specific indication only' answer this question:***<br>Please specify when 68-gallium-dotatate(toc/noc)-PET-CT is used at your institution in patients with MEN1	
4.26	***If 'How is 68-gallium-dotatate(toc/noc)-PET/CT used at your institution in patients with MEN1? Please check all that apply.' is equal to 'Regular use in surveillance of ___prevalent non-metastatic___ NETs' answer this question:***<br>If (gallium-dota-pet) is regularly used in surveillance of ___non-metastatic NETs___, with what frequency?	◯ Yearly<br>◯ Every two years<br>◯ Other<br>◯ Unknown/unable to answer
4.27	***If 'If (gallium-dota-pet) is regularly used in surveillance of non-metastatic NETs, with what frequency?' is equal to 'Other' answer this question:***<br>Please specify the frequency	
	GLP1 analogue PET-CT	
4.29	***If 'Are adults with MEN1 managed at your institution?' is equal to 'Yes, we see patients for diagnosis, screening, surveillance, treatment and follow-up' answer this question:***<br>Is Exendin (GLP1-analogue) -PET/CT used for patients with MEN1-related insulinoma at your institution?	◯ No, we do not have availability of Exendin PET-CT<br>◯ No, we have availability but do not use it<br>◯ No, we do not have availability, but we do refer to other centers for exendin-PET/CT if necessary<br>◯ Yes<br>◯ Unknown/ Unable to answer
	Frequency and modality of pancreatic surveillance imaging	
4.31	***If 'Are adults with MEN1 managed at your institution?' is equal to 'Yes, we see patients for diagnosis, screening, surveillance, treatment and follow-up' answer this question:***<br>What is the recommended surveillance frequency at your institution for small PanNETs where the decision is made not to intervene?	◯ Every six months<br>◯ Every year<br>◯ Every 2 years<br>◯ Initially every six months, if stable every year<br>◯ Initially every six months, if stable every year and perhaps longer interval if continuously stable<br>◯ Other<br>◯ Unknown/ Unable to answer

4.32	***If 'What is the recommended surveillance frequency at your institution for small PanNETs where the decision is made not to intervene?' is equal to 'Other' answer this question:***<br>Please specify other recommended surveillance frequency	
4.33	***If 'Are adults with MEN1 managed at your institution?' is equal to 'Yes, we see patients for diagnosis, screening, surveillance, treatment and follow-up' answer this question:***<br>What is/are the preferred imaging modality/modalities used for surveillance of PanNETs when the decision is made not to intervene? Please select all that apply	☐ CT<br>☐ MRI<br>☐ EUS<br>☐ 68-gallium-dotatate(toc/noc)-PET/CT<br>☐ Other<br>☐ Unknown/Unable to answer
4.34	***If 'What is/are the preferred imaging modality/modalities used for surveillance of PanNETs when the decision is made not to intervene? Please select all that apply' is equal to 'Other' answer this question:***<br>Please specify other preferred imaging modality	
	Frequency and modality of thoracic surveillance imaging	
4.36	***If 'Are adults with MEN1 managed at your institution?' is equal to 'Yes, we see patients for diagnosis, screening, surveillance, treatment and follow-up' answer this question:***<br>What is the preferred ___thoracic___ imaging modality for periodical screening in patients with MEN1 at your institution? Check all that apply.	☐ CT<br>☐ MRI<br>☐ 68-gallium-dotatate(toc/noc)-PET/CT<br>☐ Other<br>☐ Unknown/unable to answer
4.37	***If 'What is the preferred thoracic imaging modality for periodical screening in patients with MEN1 at your institution? Check all that apply.' is equal to 'Other' answer this question:***<br>Please specify other preferred thoracic imaging modality for periodical screening in MEN1	
4.38	***If 'Are adults with MEN1 managed at your institution?' is equal to 'Yes, we see patients for diagnosis, screening, surveillance, treatment and follow-up' answer this question:***<br>What is the recommended frequency for thoracic imaging in patients with MEN1 ___without thoracic NETs___ at your institution?	○ Every year<br>○ Every two years<br>○ Every three years<br>○ Every five years<br>○ Other<br>○ Unknown/ unable to answer
4.39	***If 'What is the recommended frequency for thoracic imaging in patients with MEN1 without thoracic NETs at your institution?' is equal to 'Other' answer this question:***<br>Please specify other recommended frequency for thoracic imaging	

4.40	***If 'Are adults with MEN1 managed at your institution?' is equal to 'Yes, we see patients for diagnosis, screening, surveillance, treatment and follow-up' answer this question:***<br>What is the recommended surveillance frequency at your institution for small lung NETs where the decision is made not to intervene?	◯ We never observe lung NETs in patients with MEN1<br>◯ Every six months<br>◯ Every year<br>◯ Initially at six months, yearly thereafter if stable<br>◯ Initially every six months, if stable every year, and perhaps longer interval if continuously stable<br>◯ Other<br>◯ Unknown/ unable to answer
4.41	***If 'What is the recommended surveillance frequency at your institution for small lung NETs where the decision is made not to intervene?' is equal to 'Other' answer this question:***<br>Please specify other surveillance frequency of small lung NETs	

If all questions have been answered to your satisfaction, please click "finish survery" in the lower right-hand corner of the screen to send in your survey.

On behalf of the ENETS MEN1 Task Force: Thank you for your participation!
